# Modern sea-level rise breaks 4,000-year stability in southeastern China

**DOI:** 10.1038/s41586-025-09600-z

**Published:** 2025-10-15

**Authors:** Yucheng Lin, Robert E. Kopp, Haixian Xiong, Fiona D. Hibbert, Zhuo Zheng, Fengling Yu, Praveen Kumar, Sönke Dangendorf, Hailin Yi, Yaze Zhang

**Affiliations:** 1 Department of Earth and Planetary Sciences, Rutgers University, Piscataway, NJ USA; 2 CSIRO Environment, Hobart, Tasmania Australia; 3 Rutgers Climate and Energy Institute, Rutgers University, New Brunswick, NJ USA; 4 School of Marine Sciences, Sun Yat-sen University, Zhuhai, China; 5 Southern Marine Science and Engineering Guangdong Laboratory (Zhuhai), Zhuhai, China; 6 Department of Environment and Geography, University of York, York, UK; 7 School of Earth Sciences and Engineering, Sun Yat-sen University, Zhuhai, China; 8 State Key Laboratory of Marine Environmental Science, Fujian Provincial Key Laboratory for Coastal Ecology and Environmental Studies, College of Ocean and Earth Sciences, Xiamen University, Xiamen, China; 9 Department of River-Coastal Science and Engineering, Tulane University, New Orleans, LA USA; 10 Department of Archaeology, Durham University, Durham, UK; 11 Guangzhou Institute of Geography, Guangdong Academy of Sciences, Guangzhou, China

**Keywords:** Climate-change impacts, Attribution, Climate and Earth system modelling

## Abstract

Quantifying physical mechanisms driving sea-level change—including global mean sea level (GMSL) and regional-to-local components (that is, sea-level budget)—is essential for reliable future projections and effective coastal management^1,2^. Although previous research has attempted to resolve China’s sea-level budget from the 1950s^3,4^, these studies capture short timescales and lack the long-term context necessary to fully assess modern sea-level rise in southeastern China^5^—one of the world’s most densely populated regions with immense socioeconomic importance^6^. Here we show that GMSL followed three distinct stages from 11,700 years before present (BP) to the modern day: (1) rapid early Holocene rise driven by the deglacial melt of land ice; (2) 4,000 years of stability from around 4200 BP to the mid-nineteenth century when regional processes dominated sea-level change; and (3) accelerating rise from the mid-nineteenth century. Our results arise from spatiotemporal hierarchical modelling of geological sea-level proxies and tide gauge data to produce site-specific sea-level budget estimates with uncertainty quantification. It is extremely likely (*P* ≥ 0.95) that the GMSL rise rate since 1900 (1.51 ± 0.16 mm year^−1^, 1*σ*) has exceeded any century over at least the past four millennia. Moreover, our analysis indicates that at least 94% of rapid modern urban subsidence is attributable to anthropogenic activities, with localized subsidence rates often exceeding GMSL rise. Such concurrent acceleration of global sea-level rise and rapid localized subsidence has not been observed in our Holocene geological record.

## Abstract (web summary)

Spatiotemporal hierarchical modelling of geological sea-level proxies and tide gauge data suggest that the modern global mean sea-level rise rate since 1900 has exceeded any century over at least the past four millennia, breaking the long-term stability observed in southeastern China.

## Sea-level budget background

Sea-level change, a direct indicator of climate change, poses substantial risks to coastal communities and will continue to do so in the coming century^2^. Accurate sea-level projections, which are crucial for managing these risks, necessitate a comprehensive understanding of the various sea-level change mechanisms operating across different spatiotemporal scales^7^. A typical method for validating our understanding of sea-level change involves comparing the sum of estimates for each physical process with the observed sea-level change, which is often referred to as a sea-level budget study^1,8^. Because of the limited availability of instrumental sea-level observations, with most satellite and tide gauge data spanning less than 30 and 60 years, respectively, most sea-level budget studies have focused on the twentieth to twenty-first century period^1,9^. Although these studies effectively explain sea-level budgets post-1900, a period representing a single mode of warming and sea-level rise within a geologically mild baseline, they may not capture the full range of long-term ocean–cryosphere dynamics, highlighting the need to assess recent changes within a broader geological context^5^.

The Holocene, the present interglacial period that began 11,700 years ago, provides a critical framework for evaluating sea-level budgets, as it encompasses several phases of global to local sea-level change^10,11^. These phases transition from a period of rapid GMSL rise during the early to middle Holocene (10–20 mm year^−1^, comparable with late twenty-first-century projections^2^) to a slowdown in the middle Holocene, with rates decreasing to less than 10 mm year^−1^ (refs. ^10,12^), followed by stabilization of GMSL during the pre-industrial late Holocene, with rates further declining to mostly  <0.3 mm year^−1^ (ref. ^13^). Although geological records provide the only long-term archive of sea-level evolution, their sparse coverage, indirect nature and inherent noise present substantial challenges to reconstructing Holocene sea-level budgets. Consequently, unlike modern sea-level budget assessments that compare the sum of reconstructed budget components (for example, ocean thermosteric expansion and land ice melting) to observed sea-level changes^9^, geological sea-level budgets often involve statistically decomposing reconstructed sea-level change from geological records^13^. This method examines various spatiotemporal scales recorded in data related to physical processes, such as long-term tectonic trends that are typically treated as temporally linear over the Holocene, and ocean dynamic signals that vary regionally on decadal to centennial timescales^8,11,14^.

Only a few previous studies have attempted to resolve geological sea-level budgets on the US Atlantic coast and Caribbean^8,11,15^, leaving long-term sea-level budgets largely unexplored outside North America. The coastline of southeastern China, one of the world’s most densely populated areas with more than 100 million residents, holds immense social and economic importance both domestically and internationally^6^. Understanding sea-level evolution along China’s coastline is therefore essential for assessing sea-level variability, quantifying human interactions with sea-level change and guiding future coastal infrastructure planning and coastal management^7,16^.

**Fig. 1:**
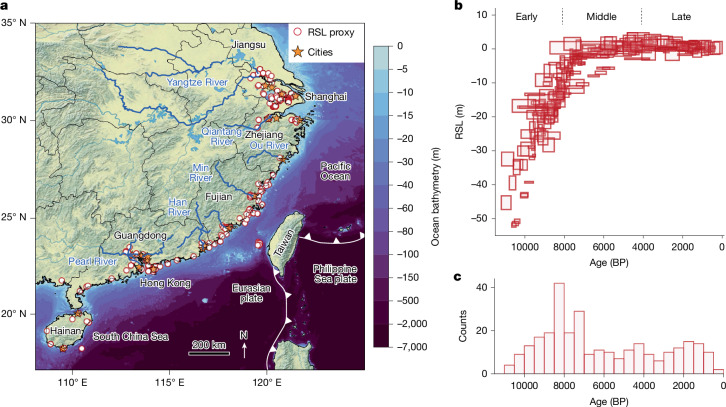
Spatiotemporal distribution of sea-level proxies and cities. **a**, Spatial distribution of sea-level proxies and main cities along the China coastline, annotated with relevant river systems, provincial boundaries and tectonic plate configurations. Cities were selected for modern sea-level budget analysis based on the concurrent availability of sea-level data and contemporary VLM observations, listed from north to south: Changzhou (Jiangsu), Wuxi (Jiangsu), Suzhou (Jiangsu), Shanghai, Hangzhou (Zhejiang), Shaoxing (Zhejiang), Ningbo (Zhejiang), Wenzhou (Zhejiang), Fuzhou (Fujian), Xiamen (Fujian), Chaozhou (Guangdong), Shantou (Guangdong), Guangzhou (Guangdong), Foshan (Guangdong), Dongguan (Guangdong), Shenzhen (Guangdong), Zhongshan (Guangdong), Hong Kong, Haikou (Hainan) and Sanya (Hainan). Ocean bathymetry data are sourced from the GEBCO database (http://www.gebco.net/) and background topography is from Natural Earth relief data (https://www.naturalearthdata.com/). **b**, RSL reconstructions across study sites for early (11700–8200 BP, referenced to 1950 CE), middle (8200–4200 BP) and late (4200 BP to present) Holocene intervals, *n* = 292. RSL and chronological data presented with 2*σ* uncertainty intervals. **c**, Temporal distribution histogram of sea-level proxies. This sea-level database was sourced from previous publications^22,30,34,51–56^.

**Extended Data Fig. 1:**
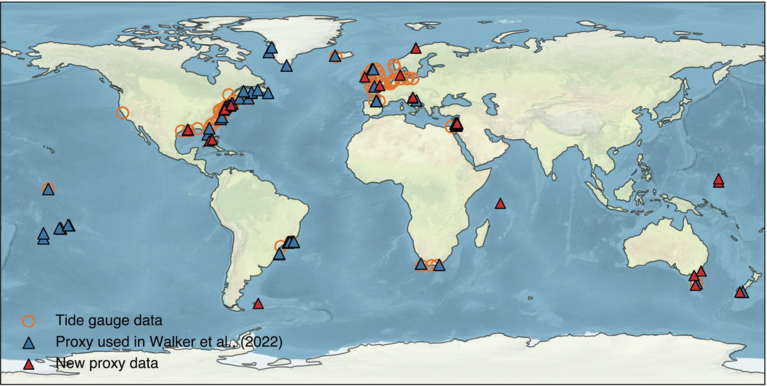
Spatial distribution of non-China sea-level data used to determine GMSL change. Triangles represent high-resolution sea-level proxy data, with blue indicating existing data from ref. ^14^ and red indicating newly incorporated proxy data. Orange circles mark locations with long-term tide gauge records from the Permanent Service for Mean Sea Level (https://psmsl.org/). Background topography is from Natural Earth Relief data (https://www.naturalearthdata.com/). This sea-level database was sourced from previous publications^14,57,68,70–79^.

We address this critical research gap through a statistical meta-analysis of global and China geological relative sea level (RSL) proxies, tide gauge data (Fig. 1 and Extended Data Fig. 1) and palaeoenvironmental records (for example, palaeoflood and palaeoshoreline sediments, precipitation proxies) to investigate Holocene sea-level changes in southeastern China. We use a spatiotemporal hierarchical modelling framework, integrating a physics-based glacial isostatic adjustment (GIA) model with Gaussian process (GP) priors to produce site-specific sea-level budget estimates with rigorous uncertainty quantification (see Methods). The reconstructed sea-level budget comprises five components: (1) a GMSL term capturing spatially uniform changes from barystatic signal and global mean thermosteric expansion; (2) a term linked to regional gravitational, rotational and deformational (GRD) effects caused by ice–ocean mass redistribution^17^; (3) a spatially varying, temporally linear signal capturing slow-changing mechanisms such as tectonics and long-term sedimentary processes^18^; (4) a regionally varying, temporally nonlinear signal driven by atmosphere/ocean dynamics and tidal range variations^8^; and (5) a locally varying, temporally nonlinear signal reflecting localized processes (for example, local-scale sediment compaction). More details of our reconstruction approach are described in Methods.

## Holocene sea-level budget

**Fig. 2:**
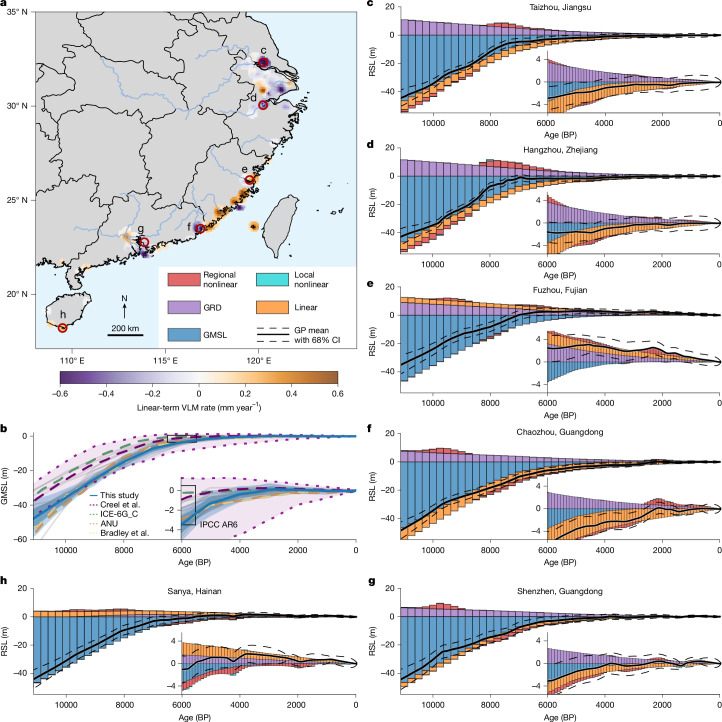
Spatiotemporal distribution of Holocene China sea level. **a**, Map showing expected linear-term (*l*(*x*, *t*)) contribution to VLM rate during the Holocene (positive indicates uplift and vice versa). Conditioning on the observations reduces the variance by at least 5% to the prior. Some of the main river channels are shown in blue. Note, the bottom-right legend applies for panels **c**–**h**. **b**, Reconstructed Holocene GMSL in comparison with previous studies (ANU^20^, Creel et al.^12^, ICE-6G_C (ref. ^19^), Bradley et al.^26^ and IPCC AR6 (ref. ^2^)). The blue lines indicate the mean from this study, the heavy and light blue shadings represent the 68% and 95% CIs from this study, respectively, and the light purple shading with dotted lines show the 90% CI from ref. ^12^. Grey lines represent further tested ice models (excluding ANU and ICE-6G_C) that inform prior GMSL distributions (see Methods). **c**–**h**, RSL and sea-level budgets evolution at six selected cities with locations shown in **a**. Sea-level budgets are separated into GMSL, GRD, linear, regional nonlinear and local nonlinear components (see Methods). Insets are zoomed-in views since 6000 BP. Previous GMSL reconstructions were compiled from refs. ^2,12,19,20,26^.

**Extended Data Fig. 2:**
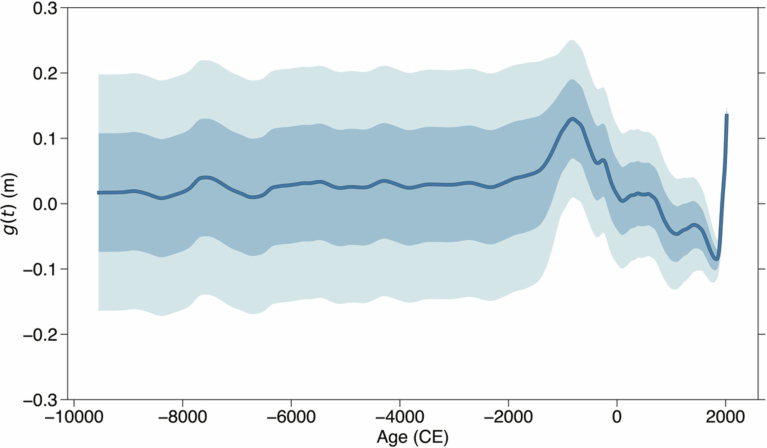
GMSL change related to spatially uniform covariance functions. This covariance function (*g*(*t*)) primarily reflects contributions from processes that are not explained by GIA_g_(*t*), such as centennial GMSL fluctuations (as GIA_g_(*t*) typically has a temporal resolution of 500–1,000 years), GMSL variation associated with mountain glaciers and global thermosteric expansion. Dark shading, 68% CI; light shading, 95% CI.

Collected from diverse geological and geomorphological settings, our sea-level database reveals notable spatiotemporal variability driven by several underlying physical processes (Fig. 1). Throughout the Holocene, GMSL rise—driven by continental ice melt from North America, Greenland, Scandinavia and Antarctica—emerged as the dominant driver of sea-level changes in China, as suggested by previous studies^12,19,20^ (Fig. 2c–h). Constrained exclusively by Chinese sea-level data before 3000 BP (0 BP = 1950 CE, reference year for radiocarbon dating; see Methods), our GMSL reconstruction is presented alongside a recent probabilistic Holocene GMSL estimate in ref. ^12^, calibrated to a global sea-level database. In the early Holocene (11700–8200 BP), driven by the final termination of North American and Scandinavian ice sheets, GMSL rose rapidly at an average rate of 10.7 ± 3.1 mm year^−1^ (1*σ*, applied consistently unless otherwise noted), aligning with the 7.2–12.3 mm year^−1^ range (68% credible interval, CI) reported ref. ^12^. By 6000 BP, the demise of the North American Ice Sheet led to a decline in the rate of GMSL rise to 2.8 ± 0.9 mm year^−1^ (0.3–2.9 mm year^−1^ in ref. ^12^), which further decreased to 0.4 ± 0.3 mm year^−1^ by 3000 BP (−0.3 to 0.7 mm year^−1^ in ref. ^12^), primarily because of meltwater from the Greenland and Antarctic ice sheets^10,21^. Subsequently, GMSL remained stable until the nineteenth century, a period marked by the onset of the Industrial Revolution and GMSL acceleration (more details in the section ‘Modern sea-level budget’). According to our reconstruction, global mean thermosteric expansion and mountain glacier melt contributed less than 0.2 m to GMSL variation (*P* ≥ 0.95; see Methods and Extended Data Fig. 2), in agreement with previous process-based reconstructions^12^.

Compared with Holocene GMSL reconstructions by Creel et al.^12^ and ICE-6G_C (ref. ^19^), our estimate indicates substantially lower GMSL during the early to middle Holocene (Fig. 2b). Although these ice models, calibrated to a worldwide sea-level database, align well with most of the North American and European sea-level data^15^, their RSL predictions are notably higher compared with observations from Asia^22^, Australia^23^, Egypt^24^ and the Arabian-Persian Gulf^25^ during the early to middle Holocene. By contrast, our reconstruction, which presents a lower GMSL during this period, aligns closely with ice histories proposed by Bradley et al.^26^ and ANU^20^, which were calibrated to a regional sea-level dataset from China and Southeast Asia, as well as a global dataset, respectively. However, early Holocene GMSL reconstruction remains an open research question with substantial uncertainties across all approaches, particularly about Antarctic Ice Sheet contributions, which often require extensive field-based ice extent surveys^10^. Our primary goal here is to provide appropriate boundary conditions for investigating regionally varying signals in China. This is supported by the ability of our model to reproduce independent RSL observations in South Asia and Australia (see Methods), its consistency with ANU^20^ and Bradley et al.^26^ and, particularly after 8000 BP, by also overlapping with the uncertainty ranges of Creel et al.^12^.

Beyond the GMSL component, four more sea-level budget components exhibit spatial variability, causing local RSL to deviate substantially from the GMSL curve (Fig. 2c–h). The GRD effects (simulated by the GIA model; see Methods), characterized by land uplift along coastlines and continental interiors and subsidence in offshore areas of China^26^, are spatially smooth across far-field regions (that is, far away from previous ice sheet margins; Fig. 2c–h). The primary variability arises from the physiography of the continental shelf, which dictates the accommodation space for ocean load intrusion and thereby influences the magnitude of solid Earth deformation. For example, the broad, flat shelf morphology near Jiangsu and Zhejiang provinces (Fig. 1) generates a GRD signal in the cities of Taizhou (Zhejiang) and Hangzhou (Zhejiang) that is 3–4 m higher than in Fuzhou and Shenzhen (Fig. 2c–g). By contrast, Sanya, located at the continental shelf margin with limited space for meltwater intrusion, experienced minimal GRD-induced RSL decrease (Fig. 2h).

The GRD effects, which include a slow-decaying viscous deformation component (see Methods), contrast with the near-instantaneous response of gravitational and rotational effects to ice sheet melting and theoretically suggests a continuous sea-level fall along China’s coastline during the middle to late Holocene when GMSL reached near the present-day level (the so-called mid-Holocene sea-level highstand^26^). However, the sea-level database reveals more complex patterns, attributable to vertical land motion (VLM). For instance, no mid-Holocene highstand is recorded in the main deltaic regions such as the Yangtze River Delta (YRD), Pearl River Delta (PRD) and Han River Delta (HRD)^22^. Furthermore, the timing of the highstand occurs much earlier along the Fujian and Taiwan coastlines, as early as 7500 BP (compared with 4000–6000 BP at Guangdong and Hainan^27^). These spatial variations reflect the unique geomorphological and geological contexts of each region, shaped by factors governing long-term VLM, including sediment compaction, sediment isostatic adjustment and tectonic activity^5,13,18^.

**Extended Data Fig. 3:**
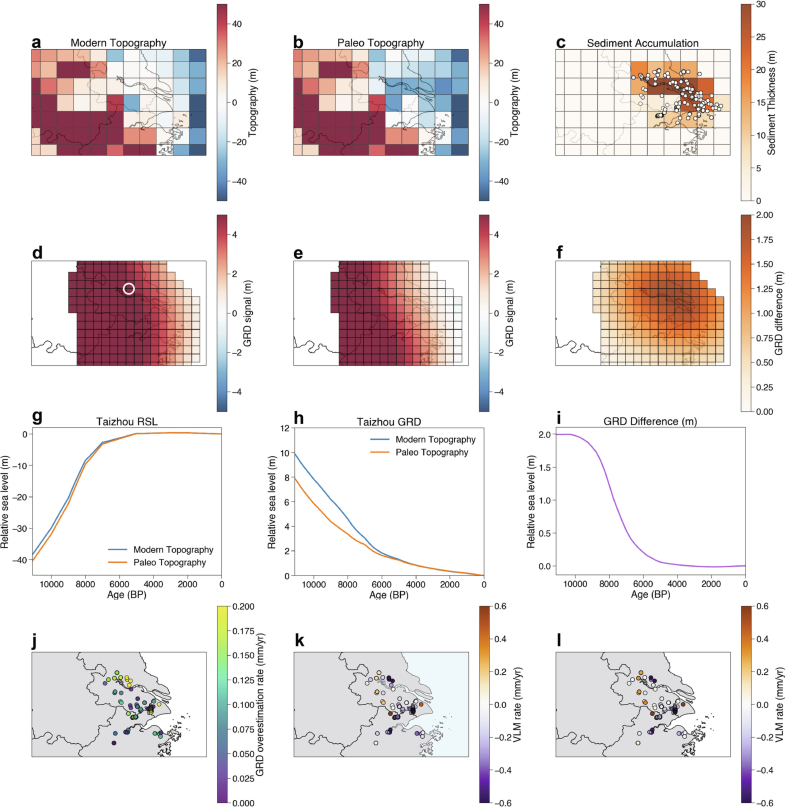
Impact of land–ocean mask misclassification on GRD calculations in the YRD. **a**, Modern topography. **b**,**c**, Reconstructed palaeotopography at 8500 BP after removing Holocene sediment thickness, with sediment coring locations shown as white dots, which is obtained from ref. ^29^. **d**,**e**, GRD signals at 8500 BP using modern and palaeotopography, respectively. **f**, Difference between modern and palaeotopography GRD signals. **g**, RSL predictions at Taizhou (location shown in **d**) using both topographies. **h**, GRD signals for Taizhou predictions. **i**, GRD signal difference at Taizhou. **j**, Linear rate of GRD overestimation at each sea-level data location. **k**,**l**, Linear term estimates before and after correcting for GRD overestimation.

Our model captures these long-term complexities through a temporally linear and locally varying kernel (see Methods), yielding millennial-average VLM rates during the Holocene that range from uplift at 1.00 ± 0.22 mm year^−1^ to subsidence at 1.37 ± 0.16 mm year^−1^ (see Fig. 2a). In depositional environments with substantial sediment input, such as deltaic plains, our reconstructions reveal spatially consistent subsidence signals, with rates of 0.59 ± 0.17 mm year^−1^ at Taizhou and 0.81 ± 0.17 mm year^−1^ at Chaozhou (HRD; Fig. 2c,f). Thicker sediment deposition in these regions is generally linked to greater sediment compaction and sediment isostatic adjustment, driving continuous local sea-level rise^18^. Borehole data from these main deltas indicate Holocene sediment deposition depths ranging from 10 to more than 70 m, with the greatest depths observed near Taizhou, Chaozhou and the offshore PRD^22,28,29^. These findings align with our long-term subsidence signals, confirming the ability of the model to identify regions in which there is extra subsidence owing to sedimentary processes. In such regions, high subsidence rates can surpass GRD-induced uplift, explaining the observed continuous rising sea-level trend (Fig. 2c,d,f). In megadelta systems such as the YRD, Holocene sediment discharge substantially altered the land–ocean boundary^29^, a process not captured in our GRD calculation. Although this may cause overestimate in reconstructed subsidence rates by up to  about 0.2 mm year^−1^, the overall subsidence pattern in the YRD remains robust (see Methods and Extended Data Fig. 3).

On the other hand, strong uplift signals are evident along the tectonically active southeastern coastline. This region lies along the active margin of the eastern Eurasian plate, adjacent to the Pacific and Philippine Sea plates. The collision and subduction of the Philippine and Pacific plates towards Taiwan and Fujian have resulted in regional and continuous uplift^30^ (Fig. 1). The highest uplift rate of 1.00 ± 0.22 mm year^−1^ can be found near the Fujian coastline, followed by a slightly lower uplift rates of up to 0.67 ± 0.17 mm year^−1^ and 0.51 ± 0.18 mm year^−1^ at Guangdong and Hainan, respectively. This overall latitudinal gradient in tectonically induced uplift rates across southeast China agrees well with estimated tectonic uplift rates derived from Marine Isotope Stage 5 marine terraces^31^. A combined uplift signal caused by tectonic and GRD effects hence explains the abnormally high mid-Holocene sea-level highstand in eastern Fujian (Fig. 2e). This finding is further supported by palaeoenvironmental reconstructions, which reveal that the Fuzhou Basin served as a marine bay environment between 7500 and 7000 BP, characterized by the dominance of brackish and brackish-marine diatoms, indicative of high RSL^27,32^.

Owing to the complex and localized geological and geomorphological conditions along China’s coastline, the linear term captures highly localized patterns. For example, the eastern Fuzhou coastline exhibits a strong uplift signal, whereas the western side shows slight subsidence adjacent to the Fuzhou Basin (Fig. 2a). This subsidence is probably driven by northeast-trending and northwest-trending faults, which have been linked to localized subsidence in the Fuzhou Basin^30,33^, as well as sedimentary processes driven by 5–40 m Holocene sediment deposition^32^. Similar variability is observed near the PRD and HRD in Guangdong, in which localized tectonic activity together with sedimentary processes probably explain the rapid transition from uplift to subsidence signals predicted by our model^34^.

**Fig. 3:**
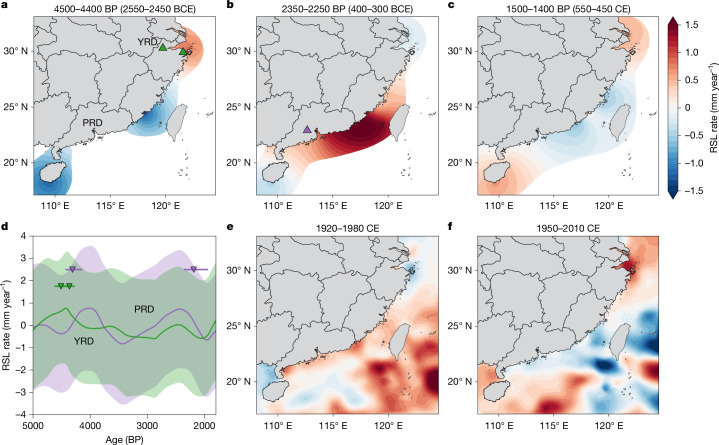
Regional nonlinear term contribution to spatiotemporal sea-level changes. **a**, RSL change rate associated with the regional nonlinear term (*r*(*x*, *t*)) between 4500 and 4400 BP (2550–2450 BCE). Conditioning on the observations reduces the variance by at least 15% to the prior. **b**,**c**, Same as **a** but for the periods between 2350 and 2250 BP (400–300 BCE) and 1500 and 1400 BP (550–450 CE), respectively. **d**, Detrended mean RSL change rates with 68% CI near Ningbo in the YRD (green) and Zhaoqing in the PRD (purple). The triangles represent notable palaeoflood events inferred from sediment stratigraphy^39,41,49^ (error bar indicates 95% age CI), with their locations marked in **a** and **b**. **e**,**f**, RSL change rates owing to ocean dynamic sea-level change, detrended to exclude climate-change signals and highlight climate variability, for the periods 1920–1980 CE (**e**) and 1950–2010 CE (**f**), smoothed with a 20-year moving average filter (source data from ref. ^9^).

Beyond the long-term linear trend, our regional nonlinear term reveals multicentennial-scale RSL fluctuations. The high uncertainty in early to middle Holocene sea-level data, combined with the overlapping influence of several physical processes (for example, tidal range variations, rapid sedimentation changes during fluvial-to-marine transitions and sediment alteration of land–ocean boundary; see Methods), complicates its interpretation for this period. Therefore, our analysis focuses on the past 5,000 years, a period when coastal environments stabilized and tidal ranges approximated present-day conditions^29^ (see Methods). The most distinctive spatial characteristic of the regional nonlinear term is a dipole-like pattern between eastern China (near the YRD) and southern China (Fujian and Guangdong) across several periods (Fig. 3). For instance, between 4500 and 4400 BP, this term contributed to a sea-level rise of up to 1.02 ± 0.91 mm year^−1^ near the YRD, while driving a sea-level fall of up to 1.63 ± 0.88 mm year^−1^ along the Fujian–Guangdong coastline—a similar pattern is also observed between 1500 and 1400 BP (Fig. 3a,c). Conversely, between 2350 and 2250 BP, an opposite pattern emerged, with a sea-level fall of up to 0.37 ± 0.78 mm year^−1^ near the YRD and a rise of up to 1.84 ± 0.79 mm year^−1^ in southern regions (Fig. 3b). These centennial-scale sea-level oscillations were also observed by high-resolution coral microatoll-based sea-level reconstructions from the South China Sea, South Asia and Australia during the middle to late Holocene^35–37^.

Several palaeoclimate proxies and dynamic modelling studies link regionally varying RSL changes to the internal climate variability (ICV), driven by complex interactions within and between climate-system components and affects sea level by altering ocean dynamics^38^. In China, key ICV drivers—the El Niño–Southern Oscillation and Pacific Decadal Oscillation—typically create asynchronous precipitation and RSL contrasts between eastern and southern regions by altering wind patterns, ocean temperatures, the monsoon rain belt and the Kuroshio Current^4,39^. A recent modern sea-level budget reconstruction^9^ shows that these effects have produced a strong regional contrast in sea-level changes across eastern and southern China during 1920–1980 and 1950–2010 (Fig. 3e–f), closely matching the dipole pattern identified in our Holocene reconstruction (Fig. 3a–c).

The link between ICV and the regional nonlinear term is further supported by several palaeoenvironmental records. Palaeoflood deposits serve as ideal indicators, reflecting the combined influence of precipitation, river discharge and regional ocean dynamic changes, particularly in estuarine environments in which most palaeo sea-level data were formed. Reconstructions in the YRD and PRD reveal that notable flood events in the PRD (southern China) often coincide with reduced precipitation in central-eastern China and vice versa^39,40^, closely mirroring the dipole pattern observed in our RSL reconstruction. Stratigraphic analyses identify two decimetre-thick flood deposits in the lower YRD at 4510 BP (4610–4410 BP) and 4370 BP (4450–4290 BP)^41^, coinciding with reconstructed regional sea-level rise (Fig. 3a,d) during a notably wet period in central-eastern China (inferred from stalagmite data^40,42^). Using an analytical model, river discharge anomalies caused by transitions between dry and wet conditions in the Qiantang River (YRD, Fig. 1) could explain tens of centimetres of ocean dynamic sea-level rise, partially accounting for the observed regional sea-level rise (see Methods). Similarly, flood deposit analysis suggests that the PRD experienced two once-in-a-millennium floods at 4310 BP (4390–4230 BP) and 2190 BP (2265–2115 BP)^39^, both probably coinciding with periods of regional sea-level rise (Fig. 3d). Furthermore, pollen and aeolian dune analysis indicate a rapid regressive phase from marine estuary environments to wetlands and shallow marshes after  around 3000 BP near the Fuzhou Basin^27^, which aligns closely with the regionally falling RSL trend in our reconstruction (Fig. 2e).

Last, the local nonlinear term, primarily reflecting site-specific RSL variability such as hydroclimate variation or seismic events, generally contributes less than 0.3 m of RSL change. For instance, ancient human activities, such as those associated with several Neolithic and Bronze Age cultures, have been shown to notably alter local hydrological systems^27,29,42^. However, owing to substantial noise in RSL proxies and the difficulty in modelling abrupt events (for example, seismic) with GP, distinguishing meaningful local signals from the present China RSL database remains challenging.

## Modern sea-level budget

**Fig. 4:**
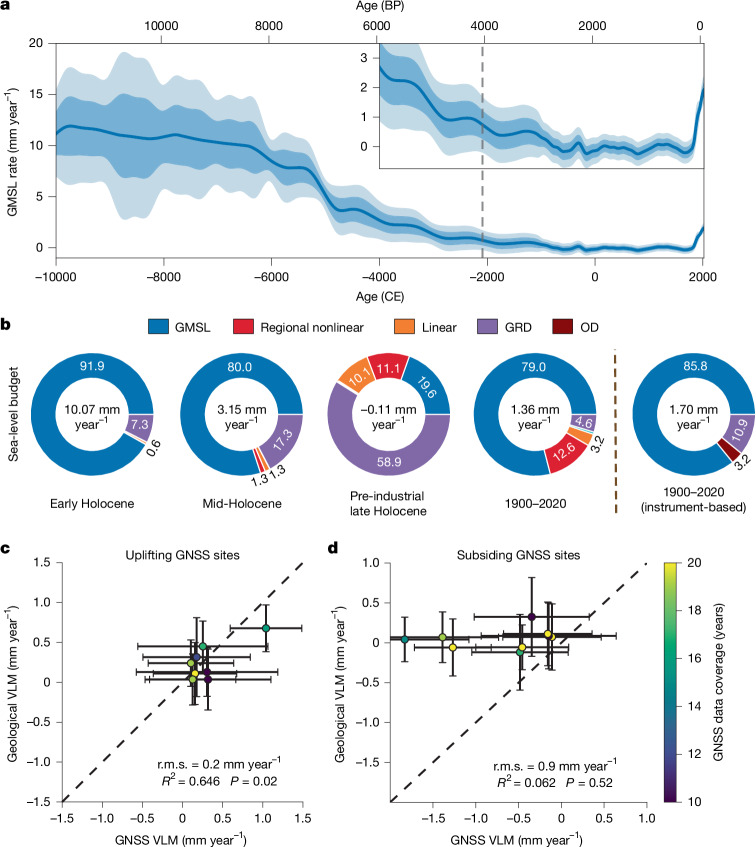
Modern China sea-level budget against its geological baseline. **a**, Reconstructed GMSL change rate during the Holocene, with the inset showing a detailed view since 4000 BCE. The dashed line marks the last geological point with a 5% probability of centennial GMSL rising faster than post-1900 rates. Dark shading represents the 68% CI and light shading represents the 95% CI. **b**, Averaged sea-level budget across 20 main coastal cities (see Fig. 1a) in southeastern China over different periods, with RSL change rates shown in the centre and percentage contributions from each component listed. Geological estimates (left four components) are derived from mean model outputs, whereas instrument-based estimates are sourced from ref. ^9^, which do not incorporate local VLM estimates. OD, ocean dynamic effects. The local nonlinear term (cyan) is indiscernible, as it accounts for less than 1% variability in all periods. **c**, Comparison of geological contributions to VLM (linear kernel + GIA-induced VLM) with modern VLM from GNSS measurements at uplift sites (obtained from the Nevada Geodetic Laboratory at the University of Nevada; see Methods). **d**, Same as **c** but for subsidence sites. Error bars represent 1*σ* reconstruction uncertainty for geological VLM and one standard error for GNSS measurements, with colours indicating GNSS data coverage periods. *R*^2^, coefficient of determination weighted by reconstruction uncertainty; *P*, statistical significance test value, calculated from weighted Pearson correlation test. Note that all three metrics here were calculated by directly comparing geological VLM with GNSS data without any further coefficients.

By using detailed understanding of the geological sea-level budget, we further assess the modern sea-level evolution for southeastern China against its geological baseline. A prominent feature of the modern sea-level budget is the accelerated GMSL rise driven by persistent atmospheric and oceanic warming related to anthropogenic forcing, which has intensified glacier and ice sheet melt, as well as ocean thermosteric expansion^43^. Our reconstructions indicate that, during the pre-industrial late Holocene (2250 BCE–1800 CE), the GMSL rise rate remained stable, with an average rate of 0.12 ± 0.29 mm year^−1^ (Fig. 4a). By 1800 CE, the probability of GMSL rise over the following decade was as likely as not (40.7%). This stability was abruptly disrupted in the nineteenth century, with the GMSL rise rate accelerating from 0.10 ± 0.18 mm year^−1^ in the first half to 0.76 ± 0.23 mm year^−1^ in the second half, aligning well with the estimated time of emergence of modern sea-level rise between 1825 and 1873 CE in ref. ^14^. Consequently, the probability of decadal GMSL rise surged to 94.1% by 1860 CE and, by 1880 CE, it became virtually certain that GMSL would continue to rise in all subsequent decades (*P* ≥ 0.99).

The twentieth and twenty-first centuries have experienced a sustained acceleration in the GMSL rise rate, leading to an average rate of 1.51 ± 0.16 mm year^−1^ between 1900 and 2020 CE. It is extremely likely (*P* ≥ 0.95) that this rate surpasses any century in the previous 40 centuries. Such rapid GMSL rise breaks the 4,000-year stability that persisted from  about 4200 BP to the mid-nineteenth century and has reshaped China’s modern sea-level budget compared with the late Holocene. To evaluate sea-level budget evolution across periods within the Holocene, we analysed the percentage contribution of each component for 20 main coastal cities in southeastern China (Fig. 1), averaging these results to derive a comprehensive view of the sea-level budget across these coastal cities (Fig. 4b; detailed city-level budgets are available in the Supplementary Information). A defining feature of the post-1900 sea-level budget is the overwhelming influence of the GMSL signal, which has increased from 19.6 ± 0.2% in the pre-industrial Holocene to 79.0 ± 1.0%, making it the primary driver of RSL changes. By contrast, GRD and regional nonlinear components, which dominated RSL changes in the late Holocene, have become relatively minor contributors since 1900 CE. This finding generally aligns with modern instrumental data analyses^9^, which attribute 85.8% of post-1900 China RSL changes to GMSL rise, with 10.9% from GRD effects and 3.2% from ocean dynamic effects. Collectively, these shifts highlight a systematic transition in the sea-level budget towards GMSL dominance, akin to early and middle Holocene conditions (Fig. 4b).

Apart from accelerating GMSL, anthropogenic-induced subsidence in coastal cities has emerged as a key feature of the sea-level budget over the past century, posing a substantial threat to coastal cities across the world^44,45^. Although modern instrumental techniques, such as the Global Navigation Satellite System (GNSS) and Interferometric Synthetic Aperture Radar (InSAR), provide precise VLM estimates for recent decades, these measurements capture the combined effects of both natural and anthropogenic VLM^46^. Our geological reconstructions can serve as a critical complementary resource, enabling a comparison between the natural and anthropogenic-induced VLM.

**Extended Data Fig. 4:**
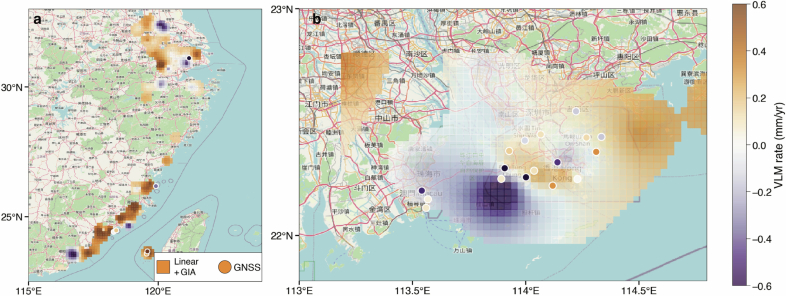
Reconstructed natural VLM rate and GNSS measurement of VLM. **a**, Comparison of natural VLM rate with GNSS data in eastern and southeastern China. Background here is based on OpenStreetMap (www.openstreetmap.org), highlighting the areas of urbanization. **b**, Same as **a** but near Hong Kong.

**Extended Data Fig. 5:**
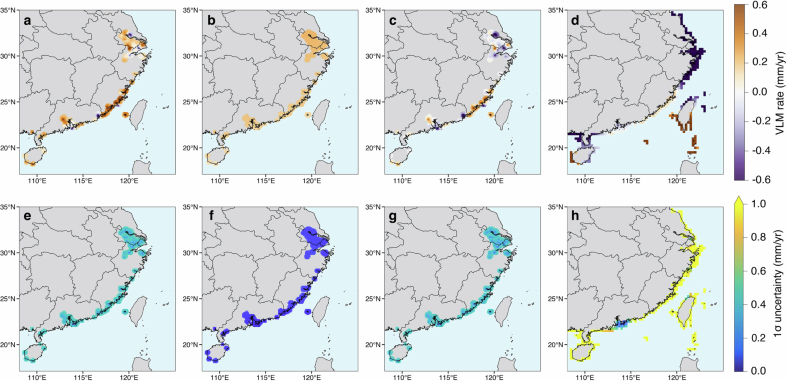
Reconstructed natural VLM rates and a modern VLM product. **a**,**e** Mean (**a**) and 1*σ* uncertainty (**e**) of the natural/geological VLM rate (a combined signal of GIA and the linear component). **b**,**f**, Mean (**b**) and 1*σ* uncertainty (**f**) of the GIA-induced VLM rate. **c**,**g**, Mean (**c**) and 1*σ* uncertainty (**g**) of the VLM rate associated with the linear component. **d**,**h**, Mean (**d**) and 1*σ* uncertainty (**h**) of the modern VLM rate from ref. ^47^, which integrates data from tide gauges, satellite altimetry and GNSS measurements.

We first validated our geological VLM estimates, comprising a combination of linear term and GIA-induced VLM (excluding GIA-induced geoid variation), against GNSS data and a recent global VLM product based on GNSS, tide gauge and satellite altimetry^47^ (see more details in Methods). Because anthropogenic activities primarily drive unidirectional subsidence (for example, groundwater extraction and infrastructure constructions), whereas uplift signals mainly reflect natural variability^45^, we compared our natural VLM rates to uplifting GNSS stations (with >0.1 mm year^−1^ rate)^48^. Among the eight uplifting GNSS stations (five from Hong Kong, three from the Fujian–Taiwan coastline; see Extended Data Fig. 4), our natural VLM estimates explain 64.6% of the total variance (*P* = 0.02; root mean square difference (r.m.s.) = 0.2 mm year^−1^)—a substantial proportion given that our reconstructions capture millennial-scale VLM variations, whereas GNSS observations span only 7–28 years (Fig. 4c). Moreover, the spatial VLM patterns derived from our linear term (by comparison, GIA signals contribute minimally to spatial variability; Extended Data Fig. 5) closely align with those reported in ref. ^47^, both showing widespread subsidence in the YRD and PRD regions and uplift along the Fujian coastline (Extended Data Fig. 5). This consistency underscores the reliability of our reconstructions in quantifying natural VLM.

When comparing natural subsidence to modern subsidence observations, a strong difference emerges against uplift signals, mostly found in less urbanized areas (Extended Data Fig. 4). Our geological VLM estimates account for only 6.2% of the variance (*P* = 0.52; r.m.s. = 0.9 mm year^−1^) at the nine subsiding GNSS stations (with  <−0.1 mm year^−1^ rate; seven from Hong Kong, one from the Fujian–Taiwan coastline and one from Shanghai), highlighting the minimal contribution of natural geological processes to the observed subsidence in the modern era (Fig. 4d and Extended Data Fig. 4). Instead, anthropogenic-induced subsidence is widely recognized as the dominant driver of rapid subsidence^45^. Unlike geological VLM, which typically progresses steadily over long timescales, anthropogenic VLM—driven by activities such as urban construction, groundwater extraction and land reclamation—is mostly characterized by high-frequency variability that can exceed natural rates by orders of magnitude, yet its impacts could be substantially mitigated through effective adaptive management strategies (for example, enhancing groundwater regulation)^46^.

Using a recent InSAR-based China VLM reconstruction that extends GNSS point measurements to city-level spatial coverage at 40-m resolution (2015–2022 CE, a period marked by extensive urban development in China^44^), we evaluated the geological and anthropogenic contributions to modern subsidence across 20 main coastal cities (see Methods). Our analysis shows that GRD effects related to Holocene deglaciation produced uplift rates of 0.15–0.23 mm year^−1^ in southeastern China, exceeding the subsidence signal of the linear term in most locations (Extended Data Fig. 5). As a result, natural subsidence is only expected in deltaic environments such as Hangzhou, Chaozhou and Shantou (Table 1). By contrast, InSAR measurements revealed widespread urban subsidence in nearly all cities except Suzhou and Dongguan. Assuming that InSAR VLM measurements represent a combined signal of natural and anthropogenic factors, our reconstructions isolate anthropogenic-induced subsidence, as shown in Table 1. With geological VLM largely manifested as uplift across southeastern China, subtracting this component from InSAR measurements reveals even greater anthropogenic subsidence levels, reflected by the  >100% anthropogenic ratios (city-median anthropogenic VLM divided by InSAR VLM measurements; Table 1). Across all subsiding cities, anthropogenic subsidence accounts for at least 94% of the total subsidence (17 out of 19 cities exceed 98%), further highlighting the dominant role of anthropogenic factors in modern subsidence signals, as opposed to the geological factors that primarily influence uplift.

**Table 1:** VLM estimates for selected coastal cities in China

City (province)	Geological VLM (mm year^−1^)	InSAR VLM (mm year^−1^)	Anthropogenic VLM (mm year^−1^)	Anthropogenic ratio (%)
Changzhou (Jiangsu)	0.2 (−0.9, 1.2)	−0.1 (−18.4, 10.3)	−0.3 (−17.5, 9.1)	264
Wuxi (Jiangsu)	0.1 (−0.8, 1.2)	−0.7 (−16.0, 7.2)	−0.8 (−15.2, 6.1)	119
Suzhou (Jiangsu)	0.1 (−1.0, 1.1)	2.5 (−11.0, 8.7)	2.5 (−10.0, 7.6)	98
Shanghai	0.2 (−1.1, 1.3)	−1.6 (−15.4, 6.3)	−1.8 (−14.3, 5.0)	113
Hangzhou (Zhejiang)	−**0.1** (−**1.1,** **1.2**)	−4.2 (−18.2, 4.2)	−4.1 (−17.1, 3.0)	98
Shaoxing (Zhejiang)	0.1 (−1.0, 1.2)	−**5.0** (−**20.1,** **2.7**)	−**5.1** (−**19.1,** **1.5**)	101
Ningbo (Zhejiang)	0.1 (−0.9, 1.1)	−3.8 (−24.6, 7.5)	−3.9 (−23.7, 6.4)	102
Wenzhou (Zhejiang)	0.2 (−0.8, 1.3)	−3.1 (−36.7, 10.0)	−3.3 (−35.9, 8.7)	107
Fuzhou (Fujian)	0.3 (−0.8, 1.4)	−**5.0** (−**30.4,** **7.8**)	−**5.3** (−**29.6,** **6.4**)	108
Xiamen (Fujian)	0.4 (−0.7, 1.6)	−1.3 (−15.8, 6.9)	−1.7 (−15.1, 5.3)	131
Chaozhou (Guangdong)	−**0.3** (−**1.6,** **1.1**)	−**5.6** (−**23.4,** **4.3**)	−**5.3** (−**21.8,** **3.2**)	94
Shantou (Guangdong)	−**0.1** (−**1.4,** **1.2**)	−4.8 (−24.3, 4.9)	−4.7 (−22.9, 3.7)	98
Guangzhou (Guangdong)	0.2 (−0.9, 1.2)	−1.6 (−17.1, 4.3)	−1.8 (−16.2, 3.1)	109
Foshan (Guangdong)	0.4 (−0.8, 1.4)	−2.5 (−15.5, 3.6)	−2.9 (−14.7, 2.2)	115
Dongguan (Guangdong)	0.1 (−0.9, 1.1)	0.2 (−10.3, 9.3)	0.1 (−9.4, 8.2)	64
Shenzhen (Guangdong)	0.1 (−1.0, 1.2)	−0.3 (−11.1, 5.2)	−0.4 (−10.1, 4.0)	121
Zhongshan (Guangdong)	0.0 (−1.1, 1.2)	−1.2 (−30.8, 5.1)	−1.2 (−29.7, 3.9)	103
Hong Kong	0.1 (−1.5, 1.2)	−1.4 (−14.1, 7.8)	−1.5 (−12.6, 6.6)	104
Haikou (Hainan)	0.1 (−0.9, 1.2)	−1.5 (−14.4, 5.3)	−1.6 (−13.5, 4.1)	110
Sanya (Hainan)	0.3 (−0.7, 1.3)	−1.5 (−19.7, 13.6)	−1.8 (−19.0, 12.3)	121

Cities were chosen on the basis of the availability of InSAR and geological sea-level data and are listed from north to south (see city locations in Fig. 1). Positive values indicate uplift and negative values represent subsidence. The geological VLM column represents the combination of the linear and GIA terms (see each estimate in Extended Data Table 1). For VLM rate columns, the primary value shown is the city spatial median and the values in brackets indicate 5th–95th percentile range, capturing both spatial variability and reconstruction uncertainty (3 mm year^−1^ for InSAR measurement^44^ and geological VLM uncertainty derived from our hierarchical model). Anthropogenic VLM represents the difference between InSAR and geological VLM. Anthropogenic ratio indicates the percentage contribution of mean anthropogenic VLM to InSAR VLM measurements. In each VLM rate column, the three cities with the highest subsidence rates are highlighted in bold. All InSAR measurements correspond to the period 2015–2022 CE, sourced from ref. ^44^.

The highest InSAR subsidence rates were observed in Chaozhou (5.6 ± 3.0 mm year^−1^, citywide median), Fuzhou (5.0 mm year^−1^), Shaoxing (5.0 mm year^−1^), Shantou (4.8 mm year^−1^) and Hangzhou (4.2 mm year^−1^). These locations correspond closely to areas in which our linear term indicates subsidence (Fig. 2a). Although Fuzhou’s median linear term generally indicates uplift, largely because of a strong signal along the eastern coast, the western basin shows subsidence rates of up to 0.3 ± 0.3 mm year^−1^ (Fig. 2a). As described in the section ‘Holocene sea-level budget’, the linear term primarily reflects local geological conditions, notably Holocene sediment thickness, that can influence modern VLM. Urban activities such as road construction, underground development and building projects can exacerbate natural sediment compaction and consolidation when imposed on thick, loose Holocene deposits, making the subsiding areas identified by our linear term (Fig. 2a) particularly vulnerable to rapid anthropogenic subsidence^46^. Incorporating anthropogenic VLM into the sea-level budget reveals a further departure against the geological baseline. Unlike geological sea-level budget variations dominated by GMSL or regional processes (Fig. 4b), it is virtually certain (*P* ≥ 0.995) that at least 5% of every city area measured by InSAR, except Dongguan, in which *P* = 0.98, exhibited local subsidence rates exceeding GMSL rise (4.06 ± 0.35 mm year^−1^ (ref. ^9^)) between 2015 and 2022.

Collectively, southeastern China’s densely populated coastal regions now face systematic sea-level challenges from global to local scales. Globally, GMSL is rising at the fastest rate in at least four millennia, with several lines of evidence indicating sustained acceleration driven by ocean warming and increasing land ice loss^2^. Regionally, both geological and instrumental reconstructions reveal that ICV-related variability has produced multidecadal sea-level fluctuations that greatly amplify regional sea-level rise and have contributed to notable flooding events^39,49^, although this uncertainty remains largely irreducible owing to its chaotic nature^38,50^. Locally, extensive urbanization has triggered rapid subsidence that can exceed GMSL rise by orders of magnitude, with the highest population densities concentrated above depositional environments highly vulnerable to anthropogenic subsidence (for example, the YRD and PRD)^44^. These converging factors underscore the need for city-specific adaptive management strategies informed by local sea-level budgets to mitigate exposure risks along China’s densely populated coastline^7,16^.

## Conclusions

We present the first, to our knowledge, process-related reconstruction of southeastern China’s sea-level budget throughout the Holocene. Validated against several palaeoenvironmental proxies and modern instrumental data, our findings enhance the understanding of China’s sea-level budget within a broader geological context. Although GMSL rise driven by deglacial ice mass loss was the dominant contributor to China sea-level changes during the Holocene, by the pre-industrial late Holocene, regional and local sea-level variability—probably driven by tectonic activity, sedimentary processes and ICV—became the primary drivers of sea-level change in several locations following GMSL stabilization. This stability was abruptly disrupted in the nineteenth century, as the GMSL rise rate accelerated from 0.10 ± 0.18 mm year^−1^ during 1800–1850 to 1.51 ± 0.16 mm year^−1^ between 1900 and 2020, marking the fastest rate observed over any century in the past four millennia.

Long-term natural VLM, shaped by diverse geological and geomorphological settings, plays a key role in driving spatial sea-level variability during the Holocene. With extensive urbanization in China, although natural processes remain the primary drivers of observed land uplift, human activities have emerged as the dominant source of subsidence in most coastal metropolitan areas. This shift is particularly pronounced in deltaic and basin environments, in which human-induced subsidence often intensifies already substantial natural subsidence signals. The combined impact of localized rapid urban subsidence and accelerated GMSL rise marks a notable departure from China’s geological sea-level budget. Without effective city-level adaptive risk management strategies, the compound sea-level rise from global to local scales can pose critical challenges to the sustainability and resilience of China’s densely populated coastal communities.

## Methods

### China RSL database

We compiled an up-to-date southeastern China RSL database, containing sea-level index points (SLIPs, data that define the discrete position of past sea level in space and time) from published literature comprising various types of geological proxy, such as diatoms, beachrock and mangroves^22,30,34,51–56^. The database was then standardized following the methodology in ref. ^57^. Each database entry consists of reconstructed RSL, RSL reconstruction uncertainty, sample age, age measurement uncertainty and a binary index indicating basal or intercalated sediment sequence (that is, whether subject to potential sediment compaction uncertainty). We did not apply any corrections to RSL change caused by tectonic or sediment compaction because we treated them as our target signals to infer from the hierarchical model. All SLIPs used in this study were radiocarbon dated: we recalibrated the conventional radiocarbon age using the IntCal20 calibration curve^58^ for terrestrial samples and Marine20 (ref. ^59^) for all marine samples. For marine samples, we apply appropriate, updated (that is, calculated using Marine20) local marine reservoir corrections (Δ*R*; http://calib.org/marine).

### Spatiotemporal statistical analysis

To estimate past trend and rates of RSL change and associated uncertainties, we used PaleoSTeHM^60^ to construct a spatiotemporal hierarchical model^61^ that consists of three hierarchical levels: (1) the data level, which describes how spatiotemporal RSL data were recorded with several uncertainty sources; (2) the process level, which models the latent process of spatiotemporal RSL change; (3) a parameter level that defines prior distributions for hyperparameters used in levels (1) and (2). For computational efficiency, we used an empirical Bayesian analysis approach; in other words, the hyperparameters used to define prior distributions are point values optimized from maximum likelihood estimation^62^.

Following the notation in ref. ^13^, at the data level, reconstructed RSL (*y*_*i*_) is modelled as:

1 $${y}_{i}=f({x}_{i},{t}_{i})+{{\epsilon }}_{i}^{y}+{y}_{0}({x}_{i})+\omega ({x}_{i},{t}_{i})+{y}_{i}\gamma $$

2 $${t}_{i}={\widehat{t}}_{i}+{{\epsilon }}_{i}^{t}$$

3 $${y}_{0}(x)\approx {\mathcal{GP}}\{0,{\sigma }_{0}^{2}\delta (x,{x}^{{\prime} })\}$$

4 $$\omega (x,t)\approx {\mathcal{GP}}\{0,{\sigma }_{\omega }^{2}\delta (x,{x}^{{\prime} })\delta (t,{t}^{{\prime} })\}$$

5 $$\gamma \approx {\mathcal{GP}}\{0,y\beta {\sigma }_{\gamma }^{2}\delta (x,{x}^{{\prime} })\delta (t,{t}^{{\prime} })\}$$

in which *x*_*i*_ is the noise-free spatial location of the *i*th observation, *t*_*i*_ is its true age, ${\widehat{t}}_{i}$ is the mean observational age and ${{\epsilon }}_{i}^{t}$ and ${{\epsilon }}_{i}^{y}$ are uncertainties in the age measurement and RSL reconstruction, respectively, which we assume to be independently and normally distributed. The notation ${\mathcal{GP}}(\mu ,k(x,{x}^{{\prime} }))$ indicates a GP prior with mean *μ* and covariance function *k*(*x*, *x*′). *y*_0_(*x*) is a site-specific datum offset to ensure that the RSL reconstructions are directly comparable with each other, *δ* is the Kronecker delta function, *ω*(*x*, *t*) is supplemental white noise and *γ* is set to allow uncertainty related to sediment compaction, in which *β* is a binary vector specifying that either each record is basal (*β* = 0) or intercalated sediment (*β* = 1) sequence. *γ* is multiplied by *y*, so a deeper sample is prone to have larger compaction uncertainty, which is a simplified formulation and may not fully capture autocompaction processes^63,64^.

We use a noisy-input GP method^65^ to translate geochronological uncertainties into corresponding vertical uncertainty using first-order Taylor series approximation:

6 $$f({x}_{i},{t}_{i})\approx f({x}_{i},{\widehat{t}}_{i})+{{\epsilon }}_{i}^{t}\frac{\partial f({x}_{i},{\widehat{t}}_{i})}{\partial t}$$

At the process level, we model the latent RSL field *f*(*x*, *t*) as a GP:

7 $$f(x,t)\approx {\mathcal{GP}}({h}_{{\rm{GIA}}}(x,t| \Theta ),\Sigma (x,t))$$

in which *h*_GIA_ serves as the mean function for the GP (that is, explicit basis function in ref. ^62^) described by linear combinations of a GIA model ensemble, which predicts RSL change at any location and time based on given physical parameters Θ = [*θ*_1_, *θ*_2_,…, *θ*_*J*_] (*J* = 360). More details about GIA model parameters are given in the GIA section below. We assume that *h*_GIA_ follows a multivariate normal distribution:

8 $${h}_{{\rm{GIA}}}(x,t| \Theta ) \sim {\mathcal{N}}({\mu }_{{\rm{GIA}}}(x,t| \Theta ),{\Sigma }_{{\rm{GIA}}}(x,t| \Theta ))$$

in which *μ*_GIA_(*x*, *t*|Θ) is a weighted mean function of the GIA model ensemble:

9 $${\mu }_{{\rm{GIA}}}(x,t)=\mathop{\sum }\limits_{j=1}^{J}{\alpha }_{j}{\rm{GIA}}(x,t| {\theta }_{j})$$

GIA(*x*, *t*|*θ*_*j*_) stands for GIA model prediction at location *x* and time *t*, given a specific set of physical model parameters *θ*_*j*_. *α*_*j*_ is the weighting factor for the corresponding GIA model, which is calculated by:

10 $${\alpha }_{j}=\frac{\exp (-0.5{\chi }_{j}^{2})}{a}$$

11 $${\chi }_{j}=\frac{1}{n}\sqrt{\mathop{\sum }\limits_{i=1}^{n}\left[\frac{{({y}_{i}-{\rm{GIA}}({x}_{i},{t}^{* }| {\theta }_{j}))}^{2}}{{({{\epsilon }}_{i}^{y})}^{2}}+\frac{{({\widehat{t}}_{i}-{t}^{* })}^{2}}{{({{\epsilon }}_{i}^{t})}^{2}}\right]}$$

in which *χ*_*j*_ represents a unitless metric to measure data-model misfit by calculating the minimum distance between each RSL data and GIA model and t* indicates the age when GIA model prediction shows the minimum distance relative to the observation^66^. *a* is a normalizing constant to ensure that the sum of all *α* values is 1 and *n* is the number of RSL data in our database. Similarly, Σ_GIA_(*x*, *t*|Θ) is the sample covariance matrix of the GIA model ensemble, weighted by *α*. The prior and posterior of the GP mean function can be found in Extended Data Table 2.

Making use of a GIA model ensemble conditioned on observational data, we decompose GIA-related contributions into GMSL change (GIA_g_(*t*); that is, barystatic signal) and associated GRD effects (GIA_GRD_(*x*, *t*)):

12 $${\rm{GIA}}(x,t)={{\rm{GIA}}}_{{\rm{g}}}(t)+{{\rm{GIA}}}_{{\rm{GRD}}}(x,t)$$

Here GIA_g_(*t*) is calculated following the methodology in ref. ^67^, incorporating the spatiotemporally variable RSL impact on calculation of ice volume above flotation and temporally evolving global ocean surface area. The GIA_GRD_(*x*, *t*) component is then derived as the difference between the local GIA model prediction and GIA_g_(*t*).

Because a mean function was set for GP prior, the GP covariance function Σ(*x*, *t*) (see equation (7)) is essentially modelling the residual between observation and mean function (that is, processes that cannot be captured by the GIA model). Putting them together, the process-level model can be written as:

13 $$\begin{array}{c}f(x,t)={{\rm{GIA}}}_{{\rm{g}}}(t)+g(t)+{{\rm{GIA}}}_{{\rm{GRD}}}(x,t)+m(x)(t-{t}_{0})+r(x,t)\\ \,+l(x,t)\end{array}$$

in which *t*_0_ is a reference time point (defined as 0 BP or 1950 CE, the reference year for radiocarbon dating). There are four more components with GP priors incorporated: (1) *g*(*t*), a common term across all sites, primarily reflecting contributions from processes that are not explained by GIA_g_(*t*), for example, centennial GMSL fluctuations (as GIA_g_(*t*) typically has a temporal resolution of 500–1,000 years), GMSL variation associated with mountain glaciers and global thermosteric expansion; (2) *m*(*x*)(*t* − *t*_0_), a spatially varying (can be either local or regional, depending on data preference), temporally linear field, which indicates slow-changing processes over the Holocene, such as tectonics, long-term sediment compaction and sediment isostatic adjustment^18,63^; (3) *r*(*x*, *t*), a regional-varying and temporally nonlinear term that reflects atmosphere/ocean dynamics-induced sea-level change and regional tidal range variation^8^; (4) *l*(*x*, *t*), a locally varying, temporally nonlinear signal that indicates highly localized processes such as small-scale sediment compaction, local hydroclimate variation and seismic events.

To further account for short-term and long-term nonlinear sea-level variability, we separate covariance functions (that is, kernels) (1), (3) and (4) into a fast (<300 years temporal length scale) and a slow (>300 years temporal length scale) component, so that equation (13) becomes:

14 $$f(x,t)={g}_{{\rm{f}}}(t)+{g}_{{\rm{s}}}(t)+{{\rm{GIA}}}_{{\rm{g}}}(t)+{{\rm{GIA}}}_{{\rm{GRD}}}(x,t)+m(x)(t-{t}_{0})+{r}_{{\rm{f}}}(x,t)+{r}_{{\rm{s}}}(x,t)+{l}_{{\rm{f}}}(x,t)+{l}_{{\rm{s}}}(x,t)$$

Therefore, the final process function contains nine components, represented by seven covariance functions, including fast and slow spatially uniform terms *g*_f_(*t*) and *g*_s_(*t*); a temporally linear term *m*(*x*)(*t* − *t*_0_); fast and slow temporally nonlinear and spatially varying terms *r*_f_(*x*, *t*) and *r*_s_(*x*, *t*); fast and slow temporally nonlinear and locally varying terms *l*_f_(*x*, *t*) and *l*_s_(*x*, *t*); and two GIA components mentioned above. Because *g*(*t*) and GIA_g_(*t*) both reflect a globally uniform signal, we interpret them together as the final GMSL change signal. Hyperparameter optimization results can be found in Extended Data Table 3.

**Extended Data Fig. 6:**
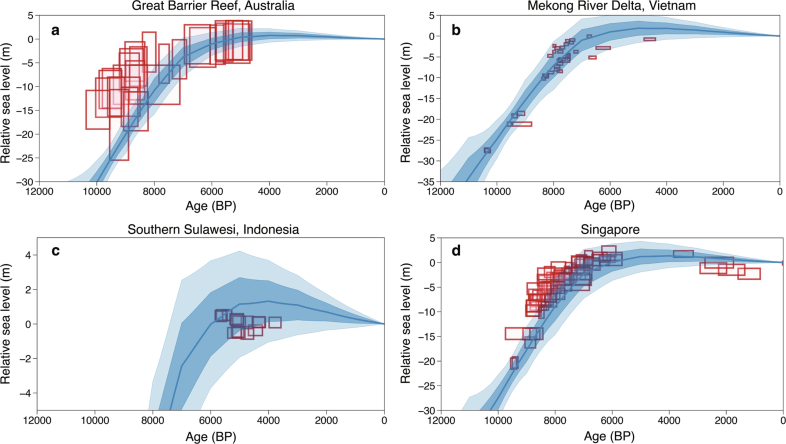
Posterior GIA model predictions of RSL at various locations outside China. **a**, Great Barrier Reef, Australia^57^. **b**, Mekong River Delta, Vietnam^68^. **c**, South Sulawesi, Indonesia^68^. **d**, Singapore^70^. Note that these predictions are weighted only by different ice models, without applying weights to the Earth model, which may vary regionally. All RSL and age data were represented by 2*σ* uncertainty boxes. Dark shading, 68% CI; light shading, 95% CI.

To ensure that our GMSL estimate captures the global signal rather than an average for China, we first tested GIA model predictions (GIA_g_(*t*) + GIA_GRD_(*x*, *t*)) against RSL data from various locations outside China, independent of our GIA ensemble calibration. The results indicate that a China-constrained GIA model prediction generally aligns well with RSL data from Australia, Singapore, the Mekong River Delta and South Sulawesi^57,68–70^ (Extended Data Fig. 6), suggesting that it serves as a good approximation of latent GMSL variation. To incorporate centennial-scale GMSL variability into our hierarchical model, we include a high-resolution GMSL reconstruction during the past three millennia. This reconstruction was developed on the basis of high-resolution SLIPs, many with sub-decadal and sub-decimetre uncertainty, with a broader set of tide gauges. This global GMSL reconstruction was generated independently of China sea-level analyses mentioned above, following the methodology in ref. ^14^, which integrates high-resolution instrumental observations with geological proxies in a consistent framework. We made some modifications, including further data from regions such as the Falkland Islands^71,72^, New Zealand^73^, Australia^74^, Pohnpei and Kosrae^75^, Israel^76^, Croatia^77^, Ireland^78^ and Florida^79^ (see Extended Data Fig. 1), and recalibrated the hyperparameters of the model accordingly (Extended Data Table 3). Furthermore, for post-twentieth-century GMSL estimates, we used the recent reconstruction in ref. ^9^ instead of that in ref. ^80^.

**Extended Data Fig. 7:**
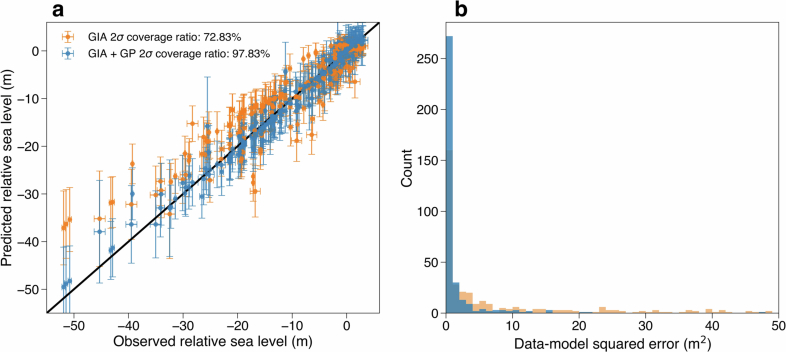
Tenfold cross-validation results. **a**, RSL observations versus predictions by our prior GIA model and hybrid GIA and GP model (GIA + GP). Horizontal and vertical error bars indicate ±2*σ* observational sea-level uncertainty and model prediction uncertainty, respectively. **b**, Histogram of weighted squared error between RSL data and model predictions, weighted by data uncertainty.

This hybrid model greatly enhances performance compared with earlier approaches that rely only on GIA models to describe observed Holocene sea-level changes^26^. Model validation through a tenfold cross-validation test demonstrates that incorporating the GP model reduces the mean squared prediction error from 13.9 m^2^ to 5.18 m^2^ and increases the percentage of observations falling within ±2*σ* of the model predictions from 72.83% to 97.83% (Extended Data Fig. 7). Furthermore, the inclusion of the GIA model ensemble not only captures the non-Gaussian dynamics of sea-level change but also provides more physically interpretable decomposition results, especially for the early Holocene (see also the purely statistical model^60^). Building on this physically informed foundation, the GP components can therefore capture further, physically meaningful variability—such as centennial-scale GMSL fluctuations and regional climate variability—that become increasingly influential in the late Holocene and modern periods.

### Mathematical formulation of the spatiotemporal covariance function

Each covariance function (that is, kernel) in equation (14) is constructed using the following common GP kernels:

15 $${K}_{{\rm{M}}}^{1/2}(a;\sigma ,\tau )={\sigma }^{2}\exp \left(-\frac{| a-{a}^{{\prime} }| }{\tau }\right)$$

16 $${K}_{{\rm{M}}}^{3/2}(a;\sigma ,\tau )={\sigma }^{2}\left(1+\sqrt{3}\times \frac{| a-{a}^{{\prime} }| }{\tau }\right)\exp \left(-\frac{\sqrt{3}| a-{a}^{{\prime} }| }{\tau }\right)$$

17 $${K}_{{\rm{Linear}}}(a;\sigma )={\sigma }^{2}(a\cdot {a}^{{\prime} })$$

in which |*a* − *a*′| can either indicate distance in time (*t*) or angular distance in space (*x*). ${K}_{{\rm{M}}}^{1/2}$ and ${K}_{{\rm{M}}}^{3/2}$ represent Matérn kernels with smoothness parameters 1/2 and 3/2; the former is non-differentiable and the latter is once differentiable. *σ* and *τ* are two kernel hyperparameters defining the amplitude and temporal/spatial length scales of that kernel. On the basis of these expressions, seven terms in our GP kernel can be written as:

18 $${g}_{{\rm{f}}}(t)={K}_{{\rm{M}}}^{3/2}(t;{\sigma }_{{{\rm{g}}}_{{\rm{f}}}},{\tau }_{{{\rm{g}}}_{{\rm{f}}}}^{t})$$

19 $${g}_{{\rm{s}}}(t)={K}_{{\rm{M}}}^{3/2}(t;{\sigma }_{{g}_{{\rm{s}}}},{\tau }_{{g}_{{\rm{s}}}}^{t})$$

20 $$m(x)(t-{t}_{0})={K}_{{\rm{M}}}^{1/2}(x;1,{\tau }_{m}^{x})\cdot {K}_{{\rm{Linear}}}(t;{\sigma }_{m})$$

21 $${r}_{{\rm{f}}}(x,t)={K}_{{\rm{M}}}^{1/2}(x;{\sigma }_{{r}_{{\rm{f}}}},{\tau }_{r}^{x})\cdot {K}_{{\rm{M}}}^{3/2}(t;1,{\tau }_{{r}_{{\rm{f}}}}^{t})$$

22 $${r}_{{\rm{s}}}(x,t)={K}_{{\rm{M}}}^{1/2}(x;{\sigma }_{{r}_{{\rm{s}}}},{\tau }_{r}^{x})\cdot {K}_{{\rm{M}}}^{3/2}(t;1,{\tau }_{{r}_{{\rm{s}}}}^{t})$$

23 $${l}_{{\rm{f}}}(x,t)={K}_{{\rm{M}}}^{1/2}(x;{\sigma }_{{l}_{{\rm{f}}}},{\tau }_{l}^{x})\cdot {K}_{{\rm{M}}}^{3/2}(t;1,{\tau }_{{l}_{{\rm{f}}}}^{t})$$

24 $${l}_{{\rm{s}}}(x,t)={K}_{{\rm{M}}}^{1/2}(x;{\sigma }_{{l}_{{\rm{s}}}},{\tau }_{l}^{x})\cdot {K}_{{\rm{M}}}^{3/2}(t;1,{\tau }_{{l}_{{\rm{s}}}}^{t})$$

### GIA modelling

RSL change signals associated with ice–water mass exchange-induced GMSL variations and GRD effects were computed using a GIA model grounded in gravitationally self-consistent theory that accounts for migrating shorelines and Earth rotational feedback^17,81,82^. Over the timescales of interest, the deformational effects exhibit viscoelastic behaviour, allowing RSL change to be separated into components representing the instantaneous response of the solid Earth and sea surface to meltwater influx and the continuing adjustments owing to past surface load changes.

The GIA model requires two critical physical inputs: solid Earth rheology and global ice history. In this study, the Earth is represented as a spherically symmetric, radially stratified (that is, 1D), self-gravitating Maxwell body comprising an elastic lithosphere and a mantle stratified into an upper mantle (extending to 670 km) and a lower mantle (extending from 670 km to the core–mantle boundary). The elastic and density structure is based on the preliminary reference Earth model^83^. Present-day topography is based on ETOPO2 (ref. ^84^). To account for uncertainties in Earth rheology, we tested lithospheric thicknesses of 71 and 96 km, upper mantle viscosities ranging from 0.05 to 0.80 × 10^21^ Pa s and lower mantle viscosities of 1 to 3 × 10^21^ Pa s, constrained by a previous GIA study on sea-level changes in China^26^.

**Extended Data Fig. 8:**
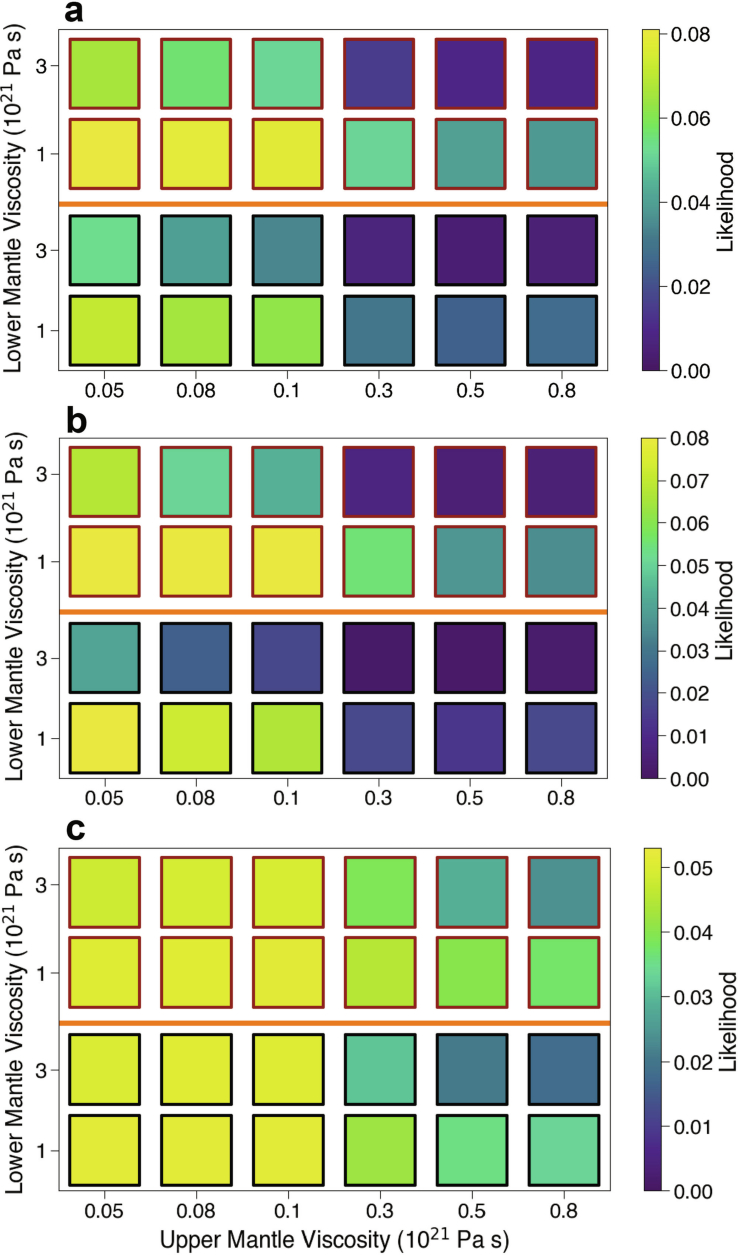
GIA Earth model calibration results. Each figure is divided into upper and lower parts by an orange line, with the upper and lower parts indicating lithospheric thickness parameters of 96 km and 71 km, respectively. **a**, Calibration results for all China sea-level data. **b**, Calibration results for northern sea-level data between 25 and 35° N. **c**, Calibration results for southern sea-level data between 15 and 25° N.

In total, 15 ice models were tested in this study, including two commonly used models, ANU^20^ and ICE-6G_C (ref. ^19^), supplemented by 13 more ice models from ref. ^85^ to bridge the sampling gap between ANU and ICE-6G_C. These extra models were generated by sampling the variability between various published ice sheet reconstructions^85^. In total, 360 ice–Earth model combinations have been computed with spherical harmonic truncation of degree and order 256 (that is, 70–80-km spatial resolution). We do not consider Earth models with laterally varying Earth rheology in this study because: (1) seismic tomography indicates minimal lateral variation in lithospheric thickness and upper mantle viscosity in southeastern China^86^, with RSL insensitive to slight lower mantle variations owing to China’s far-field location^20^; (2) calibrating our GIA model ensemble to southern (15–25° N) and northern (25–35° N) data subsets favours similar Earth parameters (Extended Data Fig. 8); (3) 1D GIA models enable comprehensive sampling of ice and Earth parameter uncertainties, the primary uncertainty source for Holocene China sea-level change, whereas the computational burden of 3D models limit such sampling. These model calibration results can be found in Extended Data Fig. 8.

Although this GIA model captures shoreline migration from ice–ocean mass exchange, it omits sediment-driven landscape evolution, which substantially reshapes the land–ocean boundary in megadelta systems such as the YRD during the Holocene. For instance, during early Holocene postglacial transgression, the elevation of Taizhou (Fig. 2c) was much lower than today, requiring tens of metres of middle to late Holocene sediment deposition to reach land elevation^29^. Thus, Taizhou should be classified as ocean until sediment accumulation elevated it. As the GRD signal typically involves coastal and continental uplift and offshore subsidence, land–ocean misclassification in GIA models using modern topography overestimates GRD calculations (whereas GMSL remains unchanged), requiring compensatory overestimation of the linear subsidence term to fit the observations.

Although developing and validating a time-evolving topography in the GIA model is beyond the scope of this study, we conducted a test to quantify an upper bound of this GRD overestimate. Using a Holocene sediment age–depth database^87^, we reconstructed palaeo YRD topography at 8500 BP (when most YRD sea-level data formed) using a similar GP formulation as our sea-level model (but with zero mean function without the fast-changing terms). Rerunning the GIA model with palaeotopography and comparing it with the original model using modern topography quantifies how the assumption of modern topography overestimates GRD, with the true process lying between these two scenarios. For this experiment, we used an Earth model with a 96-km lithosphere, 0.01 × 10^21^ Pa s upper mantle and 1 × 10^21^ Pa s lower mantle viscosity (best-fitting Earth model for China sea-level data) paired with a best-fitting ice model (model 1219 in ref. ^85^).

The palaeotopography experiment results show that the land–ocean mask was located further inland during the early Holocene when middle to late Holocene sediment deposits are removed (Extended Data Fig. 3c). This landward mask shift suggests an overall GRD overestimate in the original GIA model assuming modern topography during the early Holocene, particularly near the Yangtze River mouth (Extended Data Fig. 3f). Following the large marine transgression during the middle and late Holocene, this GRD overestimate rapidly decreased to near zero between 6000 and 5000 BP as regional GRD in the YRD is governed by broader-scale uplift signals related to continental shelf inundation, which produce nearly identical signals in both experiments. We quantified this GRD overestimate for each sea-level data point based on location and age to assess its potential impact on our linear term. Removing this potential GRD overestimate from the linear term reconstruction indicates that, although the land–ocean mask change leads to slight overestimation of subsidence rates, the overall subsidence pattern in the YRD remains robust (Extended Data Fig. 3l).

### Palaeo tidal variation impact on sea-level reconstruction

Palaeo sea-level reconstructions rely on a quantifiable relationship between a specific sea-level indicator and the contemporaneous reference water level and indicative range^88^. This relationship is commonly constructed on the basis of modern field-based survey relative to modern tidal datum, assuming that there is no change in tidal range through time^88^. However, several tidal modelling analyses have suggested that tidal regimes are subjected to great variation through time owing to sea-level change and landscape evolution^53,89,90^. As a result, assuming temporally uniform reference water level and tidal range can result in systematic bias in sea-level reconstructions and their associated uncertainties.

Systematically quantifying temporally varying tidal regime impact on sea-level reconstruction requires an accurate estimate on sea-level change and landscape evolution, owing to sediment transport in the past. This requires extensive field surveys and modelling efforts and is at present beyond the scope of this study. However, here we use a global postglacial tidal modelling study^89^ to demonstrate the potential impact of tidal variation. This study uses a truly global barotropic ocean tide model that considers the non-local effect of self-attraction and loading and can produce model tide with 65–70% of agreement compared with tide gauge measurements. Although this model only uses one postglacial sea-level change history, produced by the ICE-6G_C ice model with the VM5a Earth model and no consideration of landscape evolution, its results can still provide some meaningful information on the potential magnitude of sea-level reconstruction errors owing to tidal variation.

On the basis of this model, we calculate the biases in sea-level reconstruction and its uncertainty for each data point, based on its reference water level and indicative range. For example, the reference water level indicative range of lower tidal flat sediment with dominantly brackish water benthic diatoms with some planktonic types is quantified as the middle between mean tide level (MTL) and mean lower low water (MLLW) and within the range of MTL and MLLW. On the basis of model output from ref. ^89^, the present-day reference level and reference level and indicative range for a lower tidal flat sediment from Hong Kong can be expressed as:

25 $${\rm{Reference}}\,{\rm{level}}=[0+(-0.8)]/2=-0.4\,({\rm{m}})$$

26 $${\rm{Indicative}}\,{\rm{range}}\,{\rm{uncertainty}}=0\,{\rm{to}}-\,0.8\,({\rm{m}})$$

in which 0 and −0.8 m are the model-produced present-day MTL and MLLW. Assuming that the indicative range stands for 4*σ* reconstruction uncertainty, the uncertainty here is 0.2 m. If the sample was dated to 9000 BP, we calculate the palaeo indicative range using the same model, which may give:

27 $${\rm{Palaeo}}\,{\rm{reference}}\,{\rm{level}}=[-0.1+(-1.2)]/2=-0.65\,({\rm{m}})$$

28 $${\rm{Palaeo}}\,{\rm{indicative}}\,{\rm{range}}=-0.1\,{\rm{to}}-\,1.3\,({\rm{m}})$$

in which −0.1 and −1.2 m are the model-produced MTL and MLLW at 9000 BP. Here the reconstructed uncertainty should be 0.3 m. The biases for sea-level reconstruction and its uncertainty can then be calculated as:

29 $${\rm{Reference}}\,{\rm{level}}\,{\rm{bias}}=-0.65-(-0.40)=-0.25\,({\rm{m}})$$

30 $${\rm{Indicative}}\,{\rm{range}}\,{\rm{uncertainty}}\,{\rm{bias}}=0.3-0.2=0.1\,({\rm{m}})$$

**Extended Data Fig. 9:**
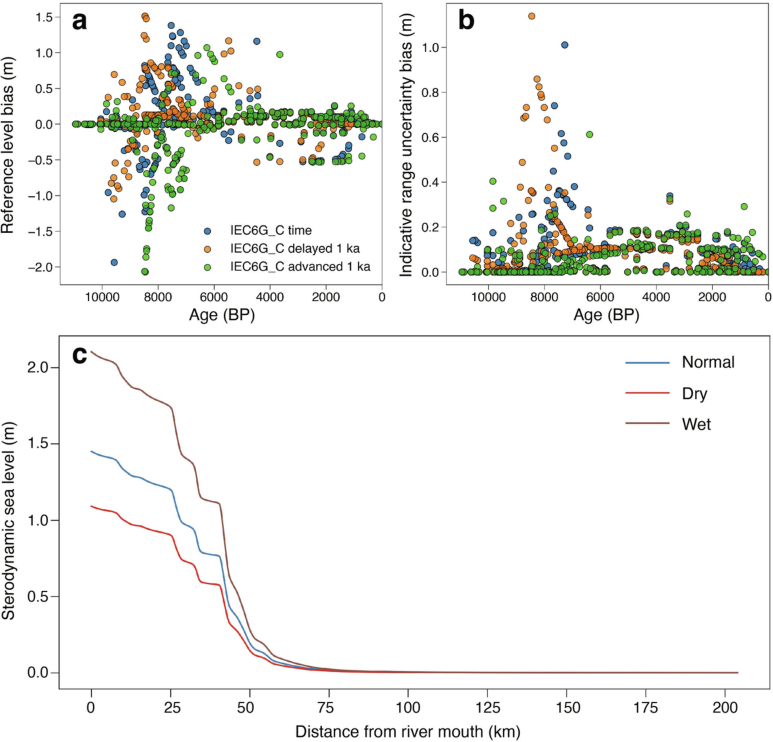
Impacts of tidal variation and sterodynamic signals on sea level. **a**, Impact of tidal variation on reference level bias in sea-level reconstruction. **b**, Impact of tidal variation on bias in indicative range uncertainty. **c**, Sterodynamic sea-level change owing to Qiantang River discharge anomalies under different weather conditions.

Overlooking tidal variations can introduce an absolute bias of 0.25 m in sea-level reconstructions, along with an extra 0.1 m of unaccounted uncertainty. Using the tidal model from ref. ^89^, we calculate all sea-level reconstruction biases and uncertainties. Because this tidal model is based on the ICE-6G_C ice history, we also assessed the impact of ice history uncertainty by performing similar calculations with tidal model time series that were either delayed or advanced by 1,000 years. The results, shown in Extended Data Fig. 9a,b, indicate that the potential influence of tidal range variation on sea-level proxy interpretation decreases substantially after about 6,000–5,000 BP, when regional sea level stabilized.

### Analytical model for river discharge impact on sea level

To calculate river discharge anomaly impact on local sterodynamic sea-level change, we applied an analytical model from ref. ^91^. Specifically, we applied the theory developed for Buenos Aires, in which local river channels are relatively shallow, narrow and fresh, similar to Qiantang River mouth near Hangzhou. Following the notation in ref. ^91^, the sterodynamic sea-level change can be written as:

31 $$\langle \zeta \rangle =\frac{{C}_{{\rm{d}}}Uq}{g}{\int }_{x}^{\infty }\frac{1}{{H}^{2}W}{\rm{d}}{x}^{{\prime} }$$

in which *ζ* represents sterodynamic sea level, *C*_d_ is the drag coefficient, *U* is a reference velocity scale, *q* is river streamflow and *g* is the acceleration owing to gravity. *H* and *W* are the river depth and width profiles, respectively. We use the default value for *C*_d_*U* from the original study (0.001 m s^−1^), whereas *H* and *W* for the Qiantang River were sourced from ref. ^92^.

On the basis of local river discharge data from 1995–2020 provided in ref. ^93^, the average discharge for wet, normal and dry years at the Qiantang River is 1,450, 1,004 and 752 m^3^ s^−1^, respectively. Using these values, the resulting sterodynamic sea-level changes related to river discharge at various distances from the river mouth are shown in Extended Data Fig. 9c.

### Modern instrumental-based sea-level budgets

To compare our reconstruction with instrument-data-based modern estimates, we used a recent analysis^9^ that describes post-1900 sea-level budgets. This analysis integrates Kalman smoother-calibrated process-based models with reduced spatial optimal interpolation of satellite altimetry to reconstruct global sea-level budgets, a method that has been used to explain sea-level budget in several regions^94,95^. For the northwestern Pacific Basin, this reconstruction achieved a 0.94 correlation coefficient with satellite altimetry data since 1993 to 2021, suggesting robust reconstruction performance.

The sea-level budget reconstruction accounts for GMSL, modern GRD (effects from recent mass exchange between the ocean, glaciers, ice sheets and water impoundment in artificial reservoirs), as well as ocean dynamic contributing to RSL change, accounting for inverse barometer effects. The ocean dynamic signal is derived by subtracting the global mean thermosteric expansion from the sterodynamic signal. For each city, we calculate each sea-level budgets component using a regional average value (that is, weighted averaging the three closest oceanic grid cells relative to the city centre). To better align the annual resolution sea-level budgets in ref. ^9^ with our geological reconstruction, we apply a 20-year moving average filter for all signals.

### Modern VLM observations

We use modern VLM rate measurements from the GNSS and InSAR. The GNSS-derived VLM rates were sourced from the Nevada Geodetic Laboratory at the University of Nevada (https://geodesy.unr.edu (ref. ^48^)), aligned to the IGS14 reference frame^96^. The VLM trends and uncertainties provided by the Nevada Geodetic Laboratory are based on the Median Interannual Difference Adjusted for Skewness (MIDAS), a robust trend estimator for accurate GNSS velocity^97^, which helps minimize the influence of seasonal variations and other temporal anomalies (for example, hydrological cycles and seismic events) that might affect trend estimation. In total, 82.6% of collected GNSS sites have a duration of at least 10 years and 69.6% have a duration of at least 15 years.

The InSAR-derived VLM rates from 2015 to 2022 CE were obtained from a national-scale assessment of land subsidence in China’s main cities^44^. This study used GNSS-calibrated Sentinel-1 data to systematically quantify land subsidence across 82 main Chinese cities. For each city, their VLM trends are presented as the median, along with the 5th and 95th percentiles, reflecting the spatial variability in VLM within each urban area. To generate geological VLM estimates from our model, we first select all coastal cities conditioning on the geological observations, which reduces the variance by at least 5% to the prior in our model. We then generated a spatial VLM map for each city within a 50-km radius and computed the same quantities as in ref. ^44^, incorporating spatiotemporal hierarchical modelling uncertainty.

## Online content

Any methods, additional references, Nature Portfolio reporting summaries, source data, extended data, supplementary information, acknowledgements, peer review information; details of author contributions and competing interests; and statements of data and code availability are available at 10.1038/s41586-025-09600-z.

## Supplementary information

Supplementary Materials Supplementary Information Fig. 1. Sea-level budget at 20 main coastal cities. For each row, the city and province names are provided as the title, along with RSL change rates for the specified period shown in the centre, and percentage contributions from each component are listed. Geological estimates (left four components) are derived from mean model outputs, whereas instrument-based estimates are sourced from ref. ^9^, which do not incorporate local VLM estimates. OD, ocean dynamic effects.

Peer Review file

## Figures and Tables

**Extended Data Table 1:**
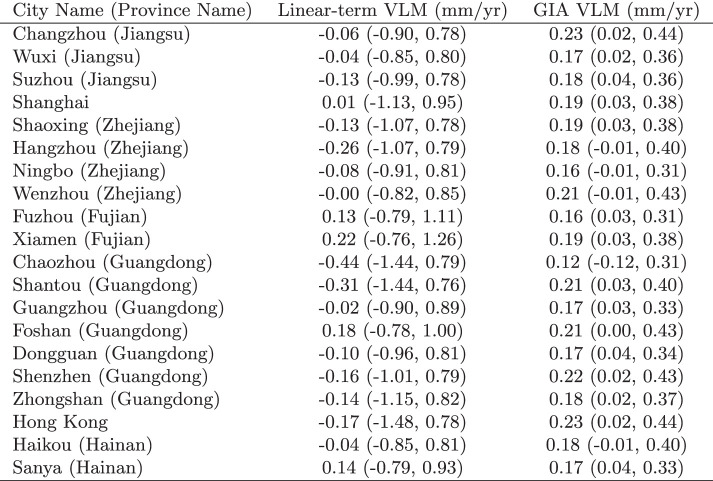
Natural VLM estimates for selected coastal cities in China

Linear-term and GIA are two natural factors contributing to VLM. Cities are listed from north to south. Positive values represent uplift and negative values indicate subsidence. The linear-term VLM and GIA VLM columns show median values alongside the 5th and 95th spatial percentiles, accounting for uncertainties in both GIA and linear estimates.

**Extended Data Table 2:**
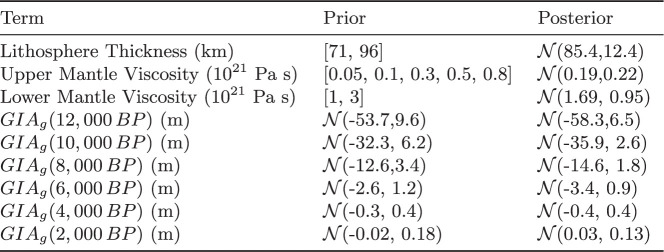
Prior and posterior GIA model parameters

We assume a discrete uniform prior for each Earth model parameter (denoted by square brackets). Ice model parameters and posterior distributions are assumed to be normally distributed. Note that the GIA_g_(*t*) here only incorporate barystatic sea-level change caused by the posterior ice model (GIA_g_(*t*)), in which *t* has unit years BP. ${\mathcal{N}}$ indicates a normal distribution.

**Extended Data Table 3:**
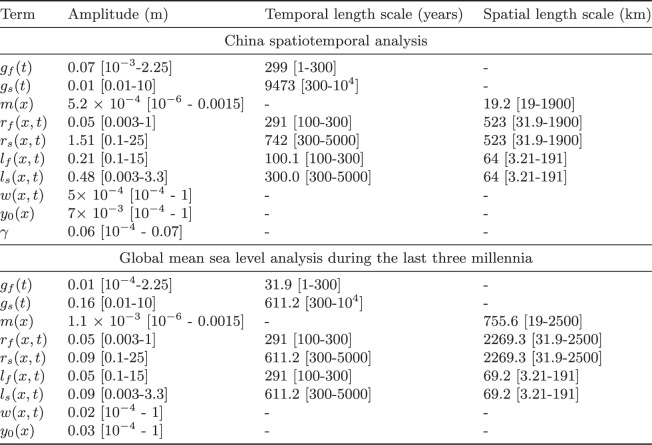
Hyperparameters for spatiotemporal statistical models

The first section shows optimized hyperparameters for the China spatiotemporal analysis. The second section presents recalibrated hyperparameters for the GMSL analysis during the past three millennia from ref. ^14^. Prior distributions for all hyperparameters are assumed to be uniformly distributed within the values in square brackets.

## Data Availability

Modern GNSS data are obtained from the Nevada Geodetic Laboratory at the University of Nevada (https://geodesy.unr.edu), the modern sea-level budget estimate in ref. ^9^ is available at 10.5281/zenodo.10621070 (ref. ^98^) and the modern VLM estimate from ref. ^47^ is available at https://zenodo.org/records/8308347 (ref. ^99^). Tide gauge data are obtained from the Permanent Service for Mean Sea Level (https://psmsl.org/). Ocean bathymetry, background topography and geographic features used for map generation in Fig. 1 and Extended Data Fig. 1 are obtained from the GEBCO database (http://www.gebco.net/) and Natural Earth Relief (https://naturalearthdata.com/), respectively. ETOPO2 can be accessed at https://www.ncei.noaa.gov/access/metadata/landing-page/bin/iso?id=gov.noaa.ngdc.mgg.dem:301. Maps in Figs. 1–3 and Extended Data Figs. 1 and 3–5 were created using cartopy (https://scitools.org.uk/cartopy)^100^. Extended Data Fig. 4 was generated on the basis of OpenStreetMap (https://www.openstreetmap.org/), which is available under Open Database License. The database used in this study, along with hierarchical modelling results—mean and uncertainty estimates of each sea-level budget component—are available at https://github.com/yc-lin-geo/China_rsl, which is archived on Zenodo with the identifier 10.5281/zenodo.16392408 (ref. ^101^).

## Code availability

PaleoSTeHM, the software used for spatiotemporal hierarchical modelling, along with several Jupyter Notebook-based tutorials for spatiotemporal hierarchical modelling, are available at https://github.com/radical-collaboration/PaleoSTeHM, which is archived on Zenodo with the identifier 10.5281/zenodo.15382745 (ref. ^102^).
