# Supplementary material for: Modern sea-level rise breaks 4,000-year stability in southeastern China
Source: Nature. 2025 Oct 15;646(8086):856–64. doi: 10.1038/s41586-025-09600-z (PMC12545208; doi:10.1038/s41586-025-09600-z)
Supplement: Supplementary file 1 — Supplementary Information Fig. 1. Sea-level budget at 20 main coastal cities. For each row, the city and province names are provided as the title, along with RSL change rates for the specified period shown in the centre, and percentage contributions from each component are listed. Geological estimates (left four components) are derived from mean model outputs, whereas instrument-based estimates are sourced from ref. 9, which do not incorporate local VLM estimates. OD, ocean dynamic effects. [file 41586_2025_9600_MOESM1_ESM.pdf]

nature portfolio

https://doi.org/10.1038/s41586-025-09600-z

Supplementary information

Modern sea-level rise breaks 4,000-year stability in southeastern China

In the format provided by the authors and unedited

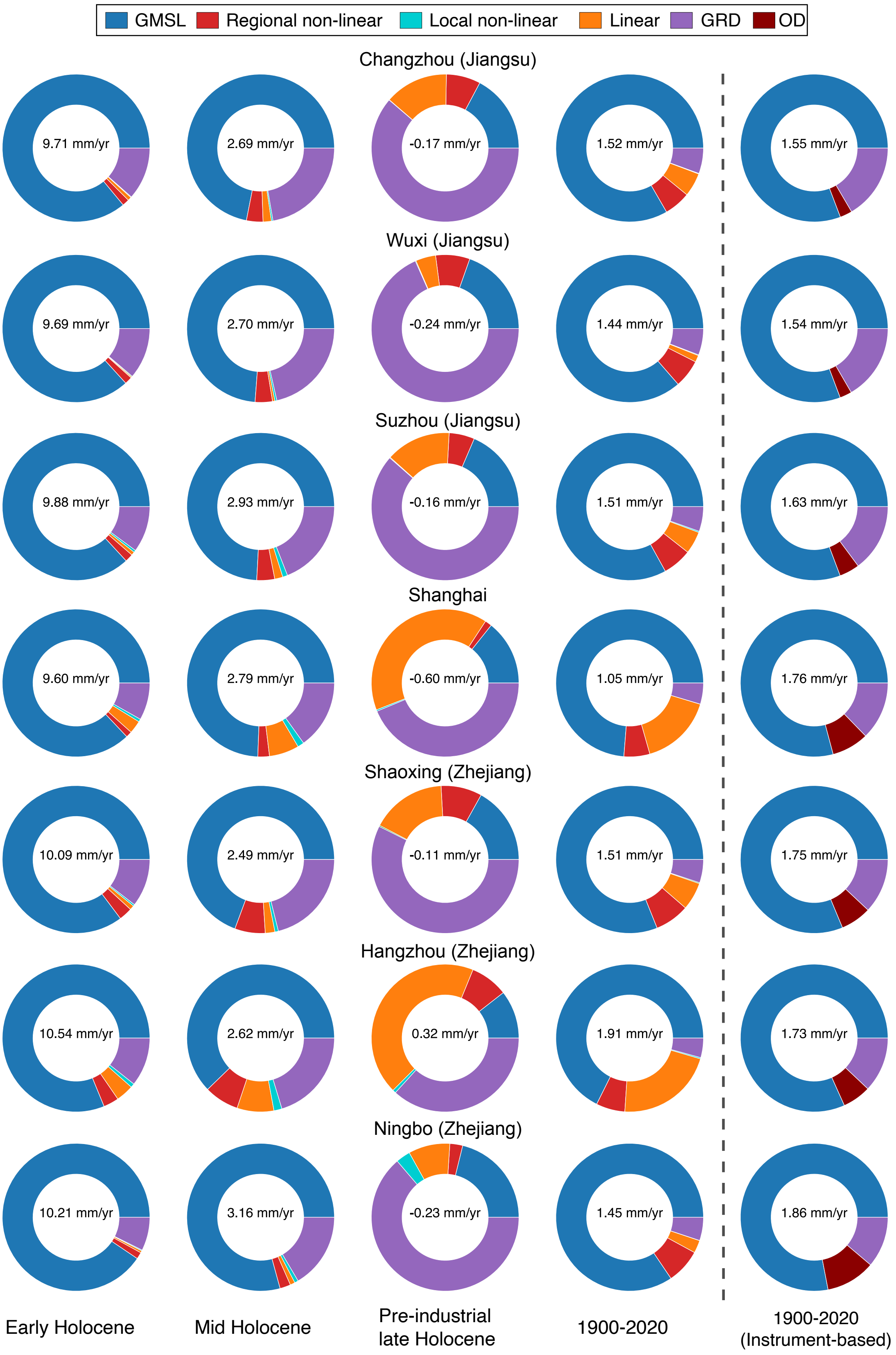
GMSL
Regional non-linear
Local non-linear
Linear
GRD
OD
Changzhou (Jiangsu)
9.71 mm/yr
2.69 mm/yr
-0.17 mm/yr
1.52 mm/yr
1.55 mm/yr
Wuxi (Jiangsu)
9.69 mm/yr
2.70 mm/yr
-0.24 mm/yr
1.44 mm/yr
1.54 mm/yr
Suzhou (Jiangsu)
9.88 mm/yr
2.93 mm/yr
-0.16 mm/yr
1.51 mm/yr
1.63 mm/yr
Shanghai
9.60 mm/yr
2.79 mm/yr
-0.60 mm/yr
1.05 mm/yr
1.76 mm/yr
Shaoxing (Zhejiang)
10.09 mm/yr
2.49 mm/yr
-0.11 mm/yr
1.51 mm/yr
1.75 mm/yr
Hangzhou (Zhejiang)
10.54 mm/yr
2.62 mm/yr
0.32 mm/yr
1.91 mm/yr
1.73 mm/yr
Ningbo (Zhejiang)
10.21 mm/yr
3.16 mm/yr
-0.23 mm/yr
1.45 mm/yr
1.86 mm/yr
Early Holocene
Mid Holocene
Pre-industrial late Holocene
1900-2020
1900-2020 (Instrument-based)

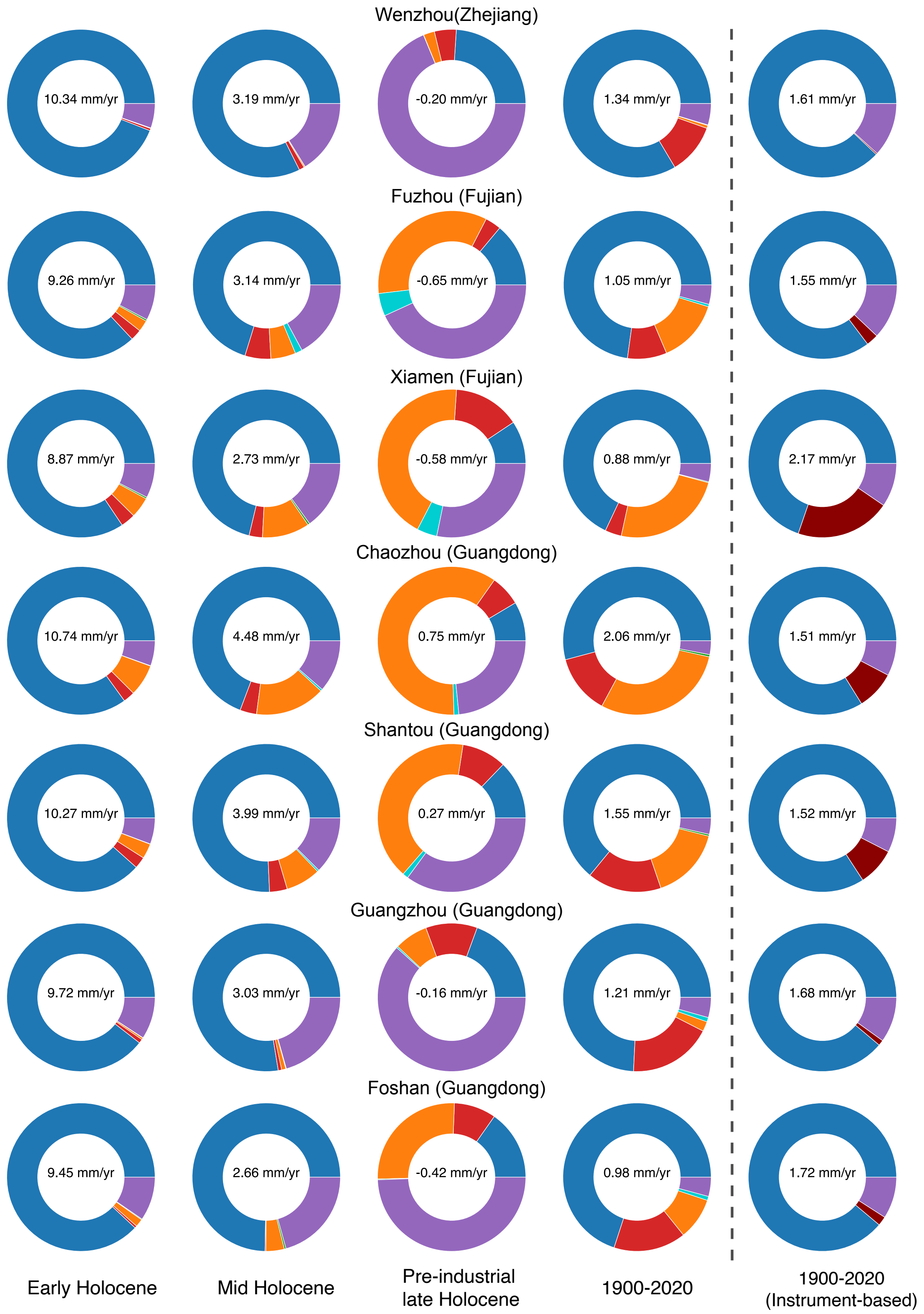
Wenzhou(Zhejiang)
10.34 mm/yr
3.19 mm/yr
-0.20 mm/yr
1.34 mm/yr
1.61 mm/yr
Fuzhou (Fujian)
9.26 mm/yr
3.14 mm/yr
-0.65 mm/yr
1.05 mm/yr
1.55 mm/yr
Xiamen (Fujian)
8.87 mm/yr
2.73 mm/yr
-0.58 mm/yr
0.88 mm/yr
2.17 mm/yr
Chaozhou (Guangdong)
10.74 mm/yr
4.48 mm/yr
0.75 mm/yr
2.06 mm/yr
1.51 mm/yr
Shantou (Guangdong)
10.27 mm/yr
3.99 mm/yr
0.27 mm/yr
1.55 mm/yr
1.52 mm/yr
Guangzhou (Guangdong)
9.72 mm/yr
3.03 mm/yr
-0.16 mm/yr
1.21 mm/yr
1.68 mm/yr
Foshan (Guangdong)
9.45 mm/yr
2.66 mm/yr
-0.42 mm/yr
0.98 mm/yr
1.72 mm/yr
Early Holocene
Mid Holocene
Pre-industrial late Holocene
1900-2020
1900-2020 (Instrument-based)

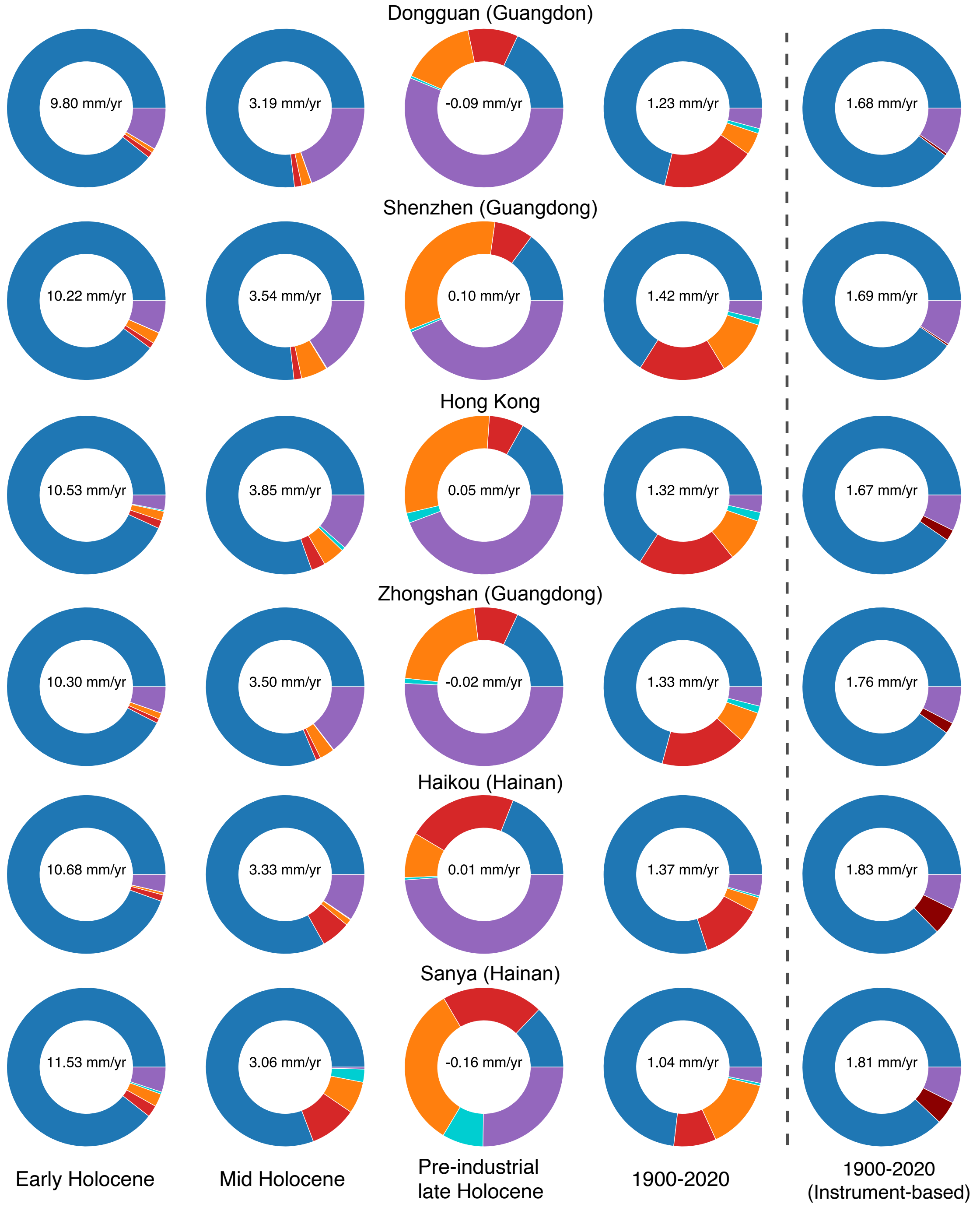

Supplementary Information Fig. 1: Sea-level budget at 20 major coastal cities. For each row, the city and province names are provided as title along with relative sea-level change rates for the specified period shown in the center, and percentage contributions from each component are listed. Geological estimates (left four components) are derived from mean model outputs, while instrument-based estimates are sourced from Dangendorf et al. (2024), which do not incorporate local VLM estimates. OD = ocean dynamic effects.

Supplementary Reference

Dangendorf, S., Sun, Q., Wahl, T., Thompson, P., Mitrovica, J.X. and Hamlington, B., 2024. Probabilistic reconstruction of sea-level changes and their causes since 1900. *Earth System Science Data Discussions*, *2024*, pp.1-37.
