# Supplementary material for: Modern sea-level rise breaks 4,000-year stability in southeastern China
Source: Nature. 2025 Oct 15;646(8086):856–64. doi: 10.1038/s41586-025-09600-z (PMC12545208; doi:10.1038/s41586-025-09600-z)
Supplement: Supplementary file 2 — Peer Review file [file 41586_2025_9600_MOESM2_ESM.pdf]

nature portfolio

Peer Review File

Modern sea-level rise breaks 4000-year stability in southeastern China

Corresponding Author: Dr Yucheng Lin

This file contains all reviewer reports in order by version, followed by all author rebuttals in order by version.

Version 0:

Reviewer comments:

Referee #1

(Remarks to the Author)
This manuscript used a spatiotemporal hierarchical model to reconstruct process-related Holocene sea-level budget across different spatiotemporal scales in southeastern China, and carried further analysis on the data. It concluded that the GMSL rise rate since 1900 CE has very likely exceeded any century over the past 4000 years, and at least 94% of the rapid modern urban subsidence is attributable to anthropogenic activities. Which is a very interesting work. My concerns are:

1.The core conclusion of this manuscript is that the GMSL rise rate since 1900 CE has exceeded any century over the past 4000 years. This conclusion is likely to arouse the interest and attention of the public, but needs to be very careful and must be based on very reliable evidence. In particular, the evidence for sea level variation over the last 4000 years should be very convincing. I noticed, however, that the past sea level data were mainly from some published geological sea level indices, such as diatom, beachrock and mangrove, which all have very big uncertainties, with uncertainty often up to 2 meters, in indicating past sea level positions. And the recent sea level data is mainly based on instrumental records, which is in millimeter-level resolution and is much much more accurate than the late-Holocene data. In this case, the sea level data between the geological-past and the instrumental post-1900 is not comparable in that high precision.

2.The Holocene sea level data used in this manuscript is mainly from literature, and many of their data was from quite old publications. For example, the review paper of Zong 2004 features sea level data collected from very old publications. Given the relatively undeveloped dating technology in the past, the previous C14 data had huge uncertainties or errors, including chronological errors/uncertainties of up to 200 years. Based on such chronological data, it is hard to obtain a reliable high-resolution (for example 100 years or less resolution), high-precision (for example millimeter- or even centimeter-scale) sea level profiles of the past. But the time-resolution of the post-1900 sea level data is up to annually or even monthly, so the sea level data between the geological centenary-scale sea level and the instrumental annual-to monthly-scale sea level are not comparable. Although the authors used some methods to correct their data for the purpose of comparability, the premise to do so must be that the raw data is reliable. Given the importance of this work, I strongly suggest the authors to re-do fieldwork (including the elevation measurements) and dating work so that they can obtain comparable data for this analysis.

3.The main conclusion of this manuscript is mainly from the data of the last 4000 years, I don’t think its necessary to include the contents regarding pre-4000 years.

Referee #2

(Remarks to the Author)
I have just finished reading the manuscript by Yucheng Lin and co-authors about the ‘Profound modern sea-level evolution against geological backdrop in southeastern China’. The authors present a thorough evaluation of sea-level changes in the region on multiple timescales, and taking into account several different processes. They conclude that early and mid-Holocene sea-level in SE China was largely controlled by deglacial land ice melt-induced GMSL, after which location-dependent regional processes became more important in the late Holocene. Over the past two centuries, accelerating GMSL rise became the dominant driver of sea-level change, locally enhanced by anthropogenic subsidence.

The manuscript is well written, and provides a comprehensive analysis of an extremely relevant topic. I am not very familiar with the technical specifics of both the hierarchical sea-level reconstructive model and the GIA models, so hopefully some other reviewers are. From what I can judge, the approach is very reasonable, and takes into account more processes (local, regional, linear, non-linear) than previous work. Several of the findings are not particularly surprising - like the importance of ice-melt induced GMSL in early and mid-Holocene, and the severity of anthropogenic subsidence - but I appreciate that the different spatiotemporal scales are translated into a common framework, and I do see the originality in that.

To me the most surprising findings are the non-linear regional processes (Figure 3), showing dipoles in relative sea-level rates. If anything, one suggestion I would have is to discuss these processes in the modern-day setting or future projections. Can we use these findings to understand where internal climate variability might enhance or counteract GMSL-rise in the future? What climatic cycles drive the periodicity in the signal? Are those cycles affected by predicted/ongoing climate change?

This is just a relatively minor remark for an otherwise impressive piece of work. To me there are no obvious flaws, and I enjoyed reading this manuscript. Below some minor language suggestions that I noted while reading.

Minor comments:

Line 23: findings
Line 38: to do so
Line 48: incorrect grammar, replace either ‘while’ or ‘which’
Line 52: The Holocene
Line 56: Maybe mention that it’s not only a slowdown of sea-level rise, there is sea-level fall as well in many locations in Mid- and Late Holocene.
Line 61: complicated sentence, had to read a few times to understand
Line 87: only uplift or also subsidence?
Line 98: that consists
Line 114: an additional
Figure 1: what’s the scale for the topography?
Line 126: component
Line 163: the Greenland
Line 193: I suppose these are cities within those provinces? Maybe specify
Figure 2: Why is a not before b in this figure?
Figure 2b: It’s difficult to understand the difference between blue and purple shading (partly because of my colorblindness I suppose.. but still). And maybe mention that Mean, 68% and 95% CI is from This Study? It’s clear from the caption, but would me more clear to also see that in the figure itself. And what are the light grey lines?
Figure 2a: It’s a bit confusing that the legend for figures c-h is put here in Figure 2a, especially since the color scheme partially overlaps with the VLM-rate. And again, possibly because I’m colorblind, but I find it difficult to distinguish between the Regional non-linear and Local non-linear colors.
Line 226: the Fujian coastline
Line 260: the South China Sea
Figure 3: maybe mark PRD and YRD on the map(s) as well
Line 290: the Fuzhou Basin
Line 298: the current
Line 341: use against or with, but not both
Line 356: remove an ‘in’
Line 431: diatoms, beachrock and mangroves
Line 433: consists of
Line 445: that consists of three hierarchical levels
Line 451: RSL is modelled as
Line 483: sun = sum
Line 485: the GP mean function
Line 491: The GIAGRD component
Line 494: that cannot be captured by the GIA model
Line 507: tide gauges
Line 545: For the northwestern Pacific Basin, this reconstruction achieved
Figure S5: but for specific cities
Figure S7: Google Maps
Line 902: result
Line 906: an accuracy estimate
Line 907: requires
Line 913: no consideration of landscape evolution
Line 918: remove one ‘of’

Referee #3

(Remarks to the Author)
Dear Authors
I have now read and reviewed the manuscript and supplementary materials to my capacity.

SUMMARY, ORIGINALITY, SIGNIFICANCE, DATA, METHODOLOGY
This work is a 'sea-level budget study' of which the analysis newly extends to geological timescales of the last millennia, to be more complete in validating attribution insights regarding global mean sea-level (GMSL) rise since the 20th cy, making good use of geological relative sea-level (RSL) data sets from SE China, and bridging this with modern datasets (measurements, satellite data). Abstract and introduction paragraphs accentuate this by mentioning a post-1900 altered situation (modern times with Anthropocene sea-level rise), following up the situation of more or less stable GMSL of the last 4 ka (late Holocene times). The RSL data stretches further back, to 11 ka, when post-glacial GMSL was still significant, as were spatially diverse geophysical responses to it. The post-1900 coverage includes statements on coastal agglomeration urban subsidence and need for tailored adaptive strategies per location, fed with RSL and GMSL budget-analysis provided constituent insights. Indeed, this part of the global coastal ribbon is heavily populated and various drivers to land subsidence and water level rise combine and exacerbate adaptation challenges. Also urbanization is associated with waves of geotechnical investigation that in the long-run tend to increase geological data available and densify the amounts of sea-level data. There is thus economic-societal demand and stimulus and timeliness for integrative, budget constituent clarifying studies for the SE Chinese coastal ribbon, which this paper answers.

To execute the work, the authors make use of their 'spatiotemporal hierarchical modeling framework', developed in recent years (Lin et al. 2024; Walker et al. 2022). The application in this study is one of the first using this framework in full over Holocene time scales, the text includes a formal section describing the sea-level budget decomposition (2.1), the supplementary materials include the modelling code. Regarding the latter: I downloaded the packages, inspected data files and documentation, including the required software libraries etc., practical issues on my side prevented me to install the actual python/notebook package in time for returning this review, but I will email the editor once I have managed to do so. For the geological data: the data included are the extracted fields used in the metadata analysis and a cross-referencing to the source publications. The data files did not copy over and disclose the full geological data provided in the source publications, and the remarks on compaction or subsidence source in there: it is not reprocessed, uniformly screened geological data. This is defendable, as the methodology as input needs 'raw RSL' (label used in the China_RSL_data.xlsx supplement) anyway, because the subsequent analysis wants to attribute constituent terms by statistical processing starting from a raw total.

The presentation of the respective processes considered in the Gaussian Process attribution, as well as the presentation of Results, begin with discussing forcings dominating the early and middle Holocene. The causal explanations for this period, rely strongly on the statistically optimized selection of GIAg(t) + GIAGRD(x, t) terms. When and where correct, the other terms in the Gaussian Process function then specify further budget contributions not captured in GMSL+GIA. When and where the GMSL+GIA optimization contains geophysical-modelling inherited bias (especially for early and middle Holocene, GIA modelling is susceptible for tradeoff effects), or data-density inherited bias (unclear if the smaller density of data for the early millennia is compensated for in the numeric selection of GIA results), the other gaussian 'kernels' will compensate that bias, by over-specifying. On this part of the paper, I have some critical remarks (see below).

The paper contains a meaningful discussion of climatic, centennial variable GMSL variations (g(t) term) during the late Holocene (results: lines 245-261; causation: lines 262-272). I think (owing to explicit constraining of timescales between 'kernels' of the Gaussian Process operationalization) that these results for the late Holocene will be independent from my concerns of GIAGRD and millennial-VLM attributions above. I have smaller remarks on this section only. The text and an inset in Fig 2A for the late Holocene, cover the more data-dense and stronger constrained part of the GMSL fitting and here I find the argumentation for credibility sufficient. I regard (as do the authors, I reckon) the main point of the paper to be to compare late Holocene to post-1900 (which abstract and introduction home into, and which is played out strongly with Figure 3 and 4).
The paper covers a great stretch of SE Chinese coast, with the Yangtze delta region in the north (Shanghai), and a gently uplifting coastal section to the south, dented with further substantial estuaries/deltas, including the Pearl river delta (Hong Kong), which is to be commended. The paper ends with a condensed confrontation and discussion (Lines 355-405) of vertical land movement (VLM) and the anthropogenically enhanced rates of that in deltaic regions being larger than the GMSL, importantly quantifying both these numbers. There is merit in combining first the topic of Holocene RSL and then that of actual sea-level rise and land subsidence, but the paper also changes topic a bit in this last section. It is a strong application example, but perhaps it takes too much space. I think Table 1 is not perse needed in main text. I wonder if the closure of the paper would not be stronger by e.g. comparing and contrasting attribution performance and antropogenic subsidence evaluations for Pearl delta, stretch in between, Yangtze Delta: brought more topical/less generic than the current final discussion paragraphs.
By the nature of the analysis: Yes, the use of statistics is appropriate, and includes formal uncertainty treatments. The text and figures display the uncertainties. The discussion of the results makes relatively little use of these uncertainties, to underpin interpretations/argumentations.
I regard conclusions robust, valid and reliable for the late Holocene and modern times, but have some concerns regarding the tradeoffs between GMSL, GIAg GIAGRD and VLM for the early and middle Holocene. See below for the major remark including suggested improvements. See below for further minor remarks.
References: no remarks.
Clarity and Content: overall fine. Minor queries.

Major remark: early and middle Holocene attribution performance
I feel the text on early and middle Holocene, and with that on GMSL, GIAg and GIAGRD respective attributions need more nuance: i.e. better textual appreciation of statistical, modeling and physical reality tradeoffs embedded in our schematizations, concepts and data collection. This is important for realism claims on the statistically-structured attribution for the early to middle Holocene (method allows good effort, but results not yet validated), as it does for the late Holocene and the modern time frames (method+data allows for very good effort, results indeed validated).
A) Detailed coverage of the early to middle Holocene results starts at line 169-175. This discusses the span of early-middle Holocene GMSL: relatively less (e.g. Creel et al. 2024 and Peltier et al. 2015: some 40 odd meters) or relatively more (Bradley et al. 2016 and Lambeck et al. 2014: some 50 odd meters; Fig. 2b). The latter position is in-line with the statistical attribution outcome of this study (also shown in Fig 2b), although it is noted that resolving the GMSL span is not the scope of the study. If there is an upper and a lower estimate, and a new study presents a third result that is close to one and less close to the other, on itself confirmation of causal correctness of the GMSL fitting. I feel more causal argument should be added to this part of the text, and just the current statement in line 182-184 'underscores its reliability for delineating GMSL trend for China' is not enough (and challengeable, as conceptually there cannot be a derived 'GMSL trend for China' that is in mismatch with that for the rest of the globe).
B) The data density for the middle to late Holocene is considerably larger than that for the early to middle Holocene (understandably). In the statistical fitting and cross-validation test of the Gaussian Process model (inspected through Figure S4a in the supplementary material), I suspect that it optimizes performance for younger time, especially in the process described in line 128-133. It appears that the 'other GPs' compensate for underfitting of the GIA GP(s) in this domain. I wonder if some weighted optimizing could have been considered that would change the relative importance of GIA and other processes in the early to middle Holocene in Fig. S4a.
C) The text and Fig 2b are used first to discuss the early-middle Holocene (GMSL result unlike Creel et al. 2024), and then the inset to discuss the late Holocene (GMSL result similar to Creel et al. 2024; GMSL high stand just above 0). For the early and middle Holocene, the statistically attributed GMSL result better matches the relatively larger GMSL span. It could be, that a larger long-term superregional subsidence signal (not well captured in global GIA modelling because of uniform rheology choices in e.g. the Peltier modelling) is misattributed to be a part of GMSL signal. I note that the Lambeck et al. 2014 study, explicitly dropped potential datapoints from e.g. the Yangtze delta (fairly dominant in early-middle Holocene domain of your dataset) as for their analysis at the time, the hard to specify extra subsidence in play in the megadelta setting. As I understand it, in case your GMSL would have been higher positioned (because… different rheology preferred, different numeric scheme preferred), this would be a tradeoff between GIAg(t) (selected to plot in Fig. 2b; a bit smaller) and GIAGRD(x,t) (a bit larger where subsiding, a bit smaller too, where uplifting), and/or the r(x,t) term modeled later on in the analysis could gain further importance in the early and middle Holocene (i.e. bigger piece of the pie in Figure 3 time series charts). Because the budget attribution chain starts with the GMSL inference from GIA modelling fitting on raw RSL data, it appears it does not get that chance. It could be, that the GIAGRD(x,t) term (continental levering, and complications in regions receiving big volumes of sediment in addition to hydrostatical loading) in physical reality is not as smooth (your statement in line 188-189) as sampled from GIA models (that have coarse spatial grids, and no consideration of crustal make up differences and exact locations of flexure) + there is potential bias in GIAGRD values surrounding megadeltas because of the land-sea masking in GIA models being based on modern topo/bathymetry (not corrected for Holocene burial thickness).
D) The results for the Yangtze megadelta, I suspect to be affected by my early-middle Holocene bias concerns. The Results section for the Yangtze delta summarizes: GIAGRD predicts uplift, RSL data points lay deep, local to regional subsidence term (VLM) is to be regarded large, and of opposite sign as GIAGRD (Taizhou, Fig. 2c), but it may be that the GIAGRD-uplift prediction is misplaced in the GIA model, as it masks land and sea with the modern delta topography. But with some reconsideration of bathymetry at the time of postglacial transgression, GIAGRD at Taizhou could be near-zero, and the predicted subsidence term smaller. GIA-modelling groups are working on that in next generation GIA modelling, but GIA runs over bathymetries undone of Holocene megadeltaic sedimentation are not yet available. The authors used current generation GIA modelling, that have 'bias' or 'artefact' imprints in megadelta areas. This is not a method flaw, but a method-input caveat, that I think needs to be more openly addressed in the presentation of results and discussion.

Minor remarks (part redactional, part scientific)
Line 52: Holocene --> The Holocene

Line 55: early to mid Holocene --> early to middle Holocene
Similarly: Line 56, 119 (Fig. 1; Caption), 170, 175, 189, 199, 201, 232, 246, 330, 359 (Fig. 4)
(mid Holocene is short speak, not official period naming, middle Holocene is better)
(Nature tends not to capitalize early/middle/late in front of geological period names)

Line 70-72: I agree with the statement, but wonder: would the recent North Sea study of Hijma et al. (2025, Nature) categorize as a long-term sea-level budget study outside North America + Caribbean? It has aspects of it, although it focuses on early and middle Holocene macro constituents and is not explicit on the further constituents in play in late Holocene and post-1900.

Line 146-147 gives a general statement, where the authors say that by specifying the GIA terms from the output of geophysical modelling, physical interpretation credit transfers to the statistical method. This is somewhat true, but I would stress that this only goes for the physics considered in the models. I see this statement as rhetoric, not full scientific justification. What is kept out of GIA models for reasons, for good parts is also physical. GIA modelling focuses on physical processes dominant in late glacial, early and middle Holocene, that level out or become background in the late Holocene (where your main use case is). In fact, you specified further processes to capture the physical reality of the Earth better, to add to the GIA-modelling selected physical processes, that become dominant in late Holocene and modern times. So it is for

the inclusion of those processes that I would like to see arguments.

Line 151-153: the sentence is written/can be read as if it is a surprise outcome, but this was the expectation/established insight: soon as you cover the full Holocene, of course melting of remnant ice-sheets of Last Glacial is your prime driver. I suggest to rewrite, incorporating the word ‘as expected’.

Line 160: ‘ By 6000 BP, the demise of the North American and Scandinavian Ice Sheets’ --> remove ‘ and Scandinavian’ (those were gone by 10000 BP already, 6000 BP relates to North America (minus Greenland) predominantly, if not solely).

Line 198: ‘ suggests a continuous sea-level fall […] during the mid- to late-Holocene’ --> ‘suggests a continuous sea-level fall […] during the late Holocene’ . The exact onset/turn over moment from rise to fall: perhaps 5.5 or 5 ka at the very end of the middle Holocene, but onset timing is trivial here. Your sentence is about the down going trend in the late Holocene primarily.

Line 201: ‘However, the sea-level database reveals more complex patterns.’ --> ‘ However, the sea-level database reveals more complex patterns, attributable to land movement’ (redactionally, I advice to give reader the cause beforehand, then exemplify). Also: for the big delta’s, this is an established expectation, see my note on Lambeck et al. (2014) mentioning it as a reason for exclusion there).

Line 209-215: for clarity repeat at the begin of this results, that the model attributes this in statistically-optimized tradeoff with the GIAGRD(x,t) term.

Line 219-218: perhaps rephrase: “The GP model thus is capable to identify the areas where there is extra subsidence due to tectonics and sedimentary loading.’

Line 274: “paleo-megafloods” --> “geologically-identified sizeable palaeoflood events”. I recommend to avoid periodically calling recurring, very large floods ‘megafloods’. Reserve that term for really crazy floods owing to glacial (mega)lake , (mega)outburst). I think in paleoflood science, there is some inflation in the use of the prefix ‘mega’. I do agree with the notion of ‘dipole pattern’ and the summary of the literature on this.

Line 280-281: double check if you are citing radiocarbon years BP or calibrated radiocarbon years BP in this particular instance.

Line 295-297: You cite three neolithic references (probably from neolithic inhabitance in the drainage basin of the rivers), but not the papers of Wang et al. 2012 and Wang et al. 2018, that you drew the sea-level data from in the supplement in the deltaic/coastal regions proper. I suggest to add these here, and use it in a way connecting back to the data explicitly used to start this. Furthermore: this Neolithic period was essentially before 5000 years ago. The period from about 5000 years ago, that you were discussing above, would match the Bronze Age (and younger archaeological periods) (the transition of Neolithic to Bronze Age is around 5000 years ago).

Line 297-299: The sentence reads: “However, due to significant noise in RSL proxies and the difficulty of modeling abrupt events (e.g., seismic) with GP, distinguishing meaningful local signals from current China RSL database remains challenging.”. I think this statement can be bend into something stronger: the whole premise of attributing and sea-level budgeting is that the full sum of terms is to be attributed, and each term as precise as possible. In principle, no noise should remain (the practical formulation of Equation 2 does not mention it; other more theoretical formulations of spatiotemporal attribution do: there is then a closing term that is a noise term – as you do in Eq. 3/Eq. 6 with the omega term). Can’t you extract from the analysis results, how much unexplained noise remains, and what greater part is model-explained? It appears you do so with the Eq.8-10 in method section, but the aspect of uncertainty in attribution did not make the Results and Discussion section.

Line 220-222: surpass the GIAGRD uplift --> this remark triggered me. For the large delta plains that are sinking, it may well be that ‘because GIA modelling uses modern day land-sea boundary for the land-sea masking’ it considers deltaic areas land through out the Holocene, and misplaces the continental-levering hinge location. It may be that the GIAGRD term is off (hinge position: what is small uplift in coastward most cells could even be small subsidence), and that the subsidence term compensates. This then leads to overattributing subsidence to tectonic and sedimentary loading causes. Especially for the Yangtze delta I would be cautious. For the other deltas, because of the topography surrounding their inner reaches, the potential for GIAGRD mis-prediction and VLM overcompensation may be less worrisome.

Line 318-319: “China’s modern sea-level budget compared to earlier Holocene stages.”
--> “China’s modern sea-level budget compared to that of the late Holocene.” I recommend to mainly contrast late Holocene to post-1900. Rates of GMSL change in the early to middle Holocene are comparable to post-1900, only late Holocene is different.

Line 340-354: For the VLM-quantified uplifting areas, I agree that GNSS helps validating.
For the VLM-quantified subsiding areas, I suspect overestimation of the subsidence rates (because of GIAGRD and tradeoff issues), and perhaps misattribution of the GNSS station rates as fully natural instead of part natural/part anthropogenic. The argument ‘the consistency underscores’ does not hold when the results are ‘right for the wrong reasons’.

Line 408-410: for the late Holocene and modern times, I agree that there has been validated. For the early and middle

Holocene, I see the results mainly as documenting GIA-modelling performance against RSL-data, and as a spin up to allow careful analysis of the Late Holocene. The budget attributions proposed here (specifically for GIAg, GIAGRD and VLM, starting from GMSL), are regionally optimized but remain yet to be validated i.m.o.

Line 456, 462: "γ is multiplied by y so a deeper sample is prone to have larger compaction uncertainty (Engelhart et al., 2015)."
There may be a technical misspecification of this property: It is not perse so that deepest intercalated samples are the strongest vertically displaced: samples in the middle of the Holocene column, have the largest potential lowering owing to 'autocompaction' processes. Towards the base the compaction is stronger, but the vertical lowering of a sample is not solely the compaction of the sample itself, but also of all material between incompressible substrate and sample.

Line 442-515: My overall compliments on the readability of technical section 4.2.

Line 516-537: You may want to mention the land-sea mask used and typical spatial resolution (tessellation, harmonics) of the GIA models.

(Remarks on code availability)
I have downloaded and reviewed the data files. They are clear.
I have downloaded but at time of submission not the reviewed code,
and for logistic reasons at my side cannot do so before passing of review deadline.

There may be follow up, in communication with the editor. From the contents of the repository, I expect to be able to install and open and inspect the notebooks, but more as an occassional code user than as a regular scientific coder myself.

Referee #4

(Remarks to the Author)
Summary
-In the manuscript titled " Profound modern sea-level evolution against geological backdrop in southeastern China", Lin et al. compile and document the sea-level budget for southeastern China since the Holocene. This is achieved through the compilation of a Holocene sea level indicator database, use of a spatiotemporal hierarchical model that combines several modeling approaches from previously published sources and glacial isostatic adjustment. Results were then placed in a modern context through calculating the modern sea-level budget and associated contributions to vertical land motion. The authors found that, up until the Pre-Industrial, sea-level rise in southeastern China was dominated by global mean sea level rise. Following a period of stability, sea level rise accelerated more than ten-fold by the 19th century, primarily driven by anthropogenic subsidence. This paper and accompanying model will compliment existing Holocene-to-modern regional sea level analyses as well as provide useful frameworks for highlighting modern sea-level rise related hazards. The work is of interest to the the broader sea level community and I recommend publication after minor revisions.
Main Comments (Manuscript)
-This manuscript takes a comprehensive approach in order to disentangle contributors to China's holocene and modern relative sea-level rise. However this same comprehensive nature, often times becomes pedantic, leading to a rather cumbersome reading experience. My suggestion would be to streamline the results and discussion section, keeping the explanation of methods to an absolute minimum in order to focus on results and implications therein. Furthermore, the reference to coastal hazards related to sea level rise—the reason this study is important—is limited and often reads as an after-thought. I believe after streamlining the "Results and Discussion" section and addressing occurrences of needlessly long sentences, this manuscript is ready for publication.

(Remarks on code availability)
-The code is easily accessed and installed thanks to a clearly written README file. The code repository consists of a main python based code that employs the PaleoSTeHM framework to calculate RSL along China's southeastern coast. Also included are 5 Juypter Notbooks, which are all easy to follow and reproduce the figures included in the manuscript.

Referee #5

(Remarks to the Author)
Dear Editor,

I have read with interest manuscript "Profound modern sea-level evolution against geological backdrop in southeastern China" by Lin et al., submitted to Nature. The paper reports evidence for recent, significant modification in the Holocene geologic regime of sea-level change in China, which points to an acceleration that is exacerbated by anthropogenic activities. The work is based on a robust statistical methodology and it is well presented. Although the results appear to be significant, it is difficult to put this work in the perspective of previous studies, since its novelty (if any) is not sufficiently highlighted, in my opinion. Furthermore, some of the sea-level "components" considered in this work suffer from significant limitations. GIA is modeled through traditional 1D models, which could be OK also in view of the significant uncertainties existing, but this would al least require some discussion. Also, the set of models chosen to construct the GIA ensemble appears to be quite ad-hoc.
Overall, this is a good work and promises to be one of high impact, especially if the two above points are addressed appropriately.

Summary of the key results: see paragraph above.
Originality and significance: Unsure about originality, see paragraph above.
Data & methodology: Data of high quality, the approach is valid but some aspects are not considered, see paragraph above. The presentation is of high level.
Appropriate use of statistics and treatment of uncertainties: fully appropriate.
Conclusions: robust, valid, reliable. See paragraph above.
Suggested improvements: Experiments with more sophisticated GIA models if possible.
References: Appropriate credit is given to previous work.
Clarity and context: The abstract/summary is lucid and appropriate. I am not sure that the introduction is enlightening the novelty of this work.

Version 2:

Reviewer comments:

Referee #1

(Remarks to the Author)
In my previous review, I raised three major concerns about this manuscript. The authors responded to my comments about using the method of citing references.
1.In my first concern, I mentioned that the sea level data between the geological-past and the instrumental post-1900 are not comparable in high resolution scale, because many sea level indices used in this work have very big uncertainties in indicating past sea level positions, with some up to 2 meters, but the recent instrumental sea level data is in millimeter-level resolution which are much more accurate. The authors emphasize that their method has been used to analyze instrumental together with geological data, and can transfer tidal gauge data from monthly/yearly to decadal resolution. While transferring data from monthly/yearly to decadal or centennial resolution is understandable, transfering the meter-scale resolution of the past sea level to millimeter-level resolution of the recent cannot be accurate. Since the core conclusion is that the GMSL rise rate since 1900 CE has exceeded any centuary over the past 4000 years, more comparable data from the geological-past will be needed.
2.My second concern is that, many C14 ages used in this manuscript have too big uncertainties (some up to 200 years) and therefore it is hard to get a reliable centennial-resolution age profile of the last 4000 years. The authors admit that 48/88% of their data exhibit 1 sigma chronological uncertainty of <100/200 years. Although the authors emphasize their data quality is better than the published data by Hijma et al. (Nature, 2025, 652-657, Global sea-level rise in the early Holocene revealed from North Sea peats), but hijma et al’s paper is about the early Holocene sea level, and that topic does not need a very high resolution of age profile. For comparing with recent instrumental records which have very accurate ages, the ages for the past should be more precise. I am glad to know that the authors are proposing a new project to carry on additional fieldwork to improved their data’s resolution, and I believe which will be more convincing.
3. My third suggestion is to remove the parts discussing about data earlier than 4000 years, since the main conclusion is mainly from the data of the last 4000 years. (Your reason?) If the authors insist to keep this part, I respect the authors’ idea.

(Remarks on code availability)

Referee #3

(Remarks to the Author)
I have now reviewed your revised manuscript, as well as the letter addressing reviewer comments.

The revised manuscript has original sections 1 and 2.1 substantially edited (sea-level budget decomposition equations moved to Methods section) and this has improved the manuscript. I regard the attention given to (i) full-holocene sea-level budget, (ii) late holocene sea-level budget and geographic differences therein, (iii) modern time accelerated sea-level rise and subsidence now well balanced: clarity of results is much improved.

The quality of data (via amending the supplement) and the presentation of data and results (via the revision) is improved: the text communicates the findings well and addresses aspects in a logical order. I no longer uphold my previous concerns whether the modern day subsidence analysis was fitting to include: the balance found in the revised manuscript allows to keep it in, and I think that these are indeed meaningful statements to make for the chain of Chinese coastal (mega)cities, adding to paper impact.

My previous technical concerns regarding trade-offs between GMSL and GIA_GRD terms were well addressed in the letter, and through the textual revisions it triggered. The same goes for the remark on the decomposed subsidence signal in the big deltas, the land-ocean mask, and further minor comments. I am satisfied with the response. I have no remaining or new technical concerns.

A minor remark concerns lines 538-541 and 547 and 554 (revised manuscript, Methods subsection 5.4) and the word

'overestimate' in there, implemented in response to my previous review. Because of the sign difference between uplift and subsidence, it is unclear what is meant exactly with 'overestimate'. Does one overestimate subsidence when one predicts uplift instead of subsidence?? I think you mean to say that using the modern land mask (main run) and using a paleo land mask (alternate run described in line 542-551) produce two bounding results for the GRD values, that sought for truth should be within. The 'GRD overestimate' that is discussed from line 554 onwards: it is not fully explicit, if that concerns the number produced in the main run (dubbed 'overestimate' as it is compared to a paleo-land-mask run) or the land movement according to the paleo-land mask run. Line 561 say it is an overestimation of the subsidence rates (not inland GRD uplift rate), which then adds to my confusion. The main text lines 157-161 and the response in the letter addressing comments is more clear than this part of the revised text. I think with some editing of section 5.4 this can be resolved/clarified. I am very happy that this caveat of doing Holocene sea-level budget analysis for large deltas, is explicit part of your treatment of the topic: I think it does matter for urban geology usage of the findings of the paper, and further natural vs. human-induced subsidence attribution in mega deltas.

Regarding the title "Profound modern sea-level evolution against geological backdrop in southeastern China": you might want to make it e.g. ".. modern sea-level _and_land_movement_ evolution against geological backdrop ..." - since you analyse the latter extensively.

+++ ADDENDUM

The editor requested to append an advice regarding remaining points raised by reviewer1.
This considers fundamental differences in temporal and vertical resolution and accuracy between instrumental era and Holocene geological data. I agree with reviewer1 and authors that this requires specific handling (e.g. transforming instrumental data to decadal means) and that the benefits of combining these is emphasized in the paper. Unlike reviewer1, I think this is appropriately handled and addressed sufficiently explicit in the study. Some specific reflection:

- It is true that the geological input data is from various background (compiled from a few decades of dispersed regional studies) and has variable age-depth accuracies, but the statistical setup (multiple ‘kernels’ in the phrasing of the authors) addresses that there is ‘noise’/‘error’/’signal to be attributed to data collection nature’ (‘local factors’).

- I do not share the reviewer1 concerns regarding detection of the present GMSL acceleration (exceeding the Late Holocene background rate), as the acceleration does not occur at the break from geological to instrumental data, but gradually within the instrumental data covered period. The call of reviewer1 to have geological data for the pre-instrumental age as accurate as instrumental data, I reckon is impossible to meet. I don’t think it is needed to make the claims the paper does either. In the end, the paper provides a budget analysis for China’s _relative_ sea-level record, synthesizing Holocene geological data (the word ‘backdrop’ in the paper title), so that analysis of instrumental data -- at their original high temporal and vertical resolution -- is supplied by meaningful, reproducible natural rates. This thus lifts up geological data and overcomes resolution caveats, for engineering and other societal use. The scientific goal is not to achieve the same resolution in the past as we have in the present.

- I found the treatment of variation in sea-level in the last 4000 years quite developed. These trends (statistically unlocked by kernels of centennial time length and few 100 km spatial range) were statistically detected, despite the lower age-depth accuracy.

- I agree that with sea-level data and its statistical processing alone (geologically observation in isolation), there are different trade offs imaginable: with the current numbers of individual observations, there is still risk of misreading sampling bias as geological signals. But the statements on the last 4000 year were not solely the geological sea-level that defines the state of research: for the last 4000 years, the authors cross-validate their inference referencing extensive hydro-climatological literature and providing mechanistic calculus providing causal supportive explanation. There is similar trade-off care in the treatment of natural subsidence and the validation of that against instrumental era data (which in critical areas records human accelerated subsidence).

(Remarks on code availability)
no comments

Referee #5

(Remarks to the Author)
Dear Editor
I am satisfied with the authors' response.
Best
G

Open Access This Peer Review File is licensed under a Creative Commons Attribution 4.0 International License, which permits use, sharing, adaptation, distribution and reproduction in any medium or format, as long as you give appropriate credit to the original author(s) and the source, provide a link to the Creative Commons license, and indicate if changes were made.
In cases where reviewers are anonymous, credit should be given to 'Anonymous Referee' and the source.
The images or other third party material in this Peer Review File are included in the article’s Creative Commons license, unless indicated otherwise in a credit line to the material. If material is not included in the article’s Creative Commons license and your intended use is not permitted by statutory regulation or exceeds the permitted use, you will need to obtain permission directly from the copyright holder.
To view a copy of this license, visit https://creativecommons.org/licenses/by/4.0/

Referees' comments:

Referee #1 (Remarks to the Author):

This manuscript used a spatiotemporal hierarchical model to reconstruct process-related Holocene sea-level budget across different spatiotemporal scales in southeastern China, and carried further analysis on the data. It concluded that the GMSL rise rate since 1900 CE has very likely exceeded any century over the past 4000 years, and at least 94% of the rapid modern urban subsidence is attributable to anthropogenic activities. Which is a very interesting work. My concerns are:

We thank the reviewer for acknowledging the merit of this study. Our response to each remark can be found below, highlighted in blue.

1.The core conclusion of this manuscript is that the GMSL rise rate since 1900 CE has exceeded any century over the past 4000 years. This conclusion is likely to arouse the interest and attention of the public, but needs to be very careful and must be based on very reliable evidence. In particular, the evidence for sea level variation over the last 4000 years should be very convincing. I noticed, however, that the past sea level data were mainly from some published geological sea level indices, such as diatom, beachrock and mangrove, which all have very big uncertainties, with uncertainty often up to 2 meters, in indicating past sea level positions. And the recent sea level data is mainly based on instrumental records, which is in millimeter-level resolution and is much much more accurate than the late-Holocene data. In this case, the sea level data between the geological-past and the instrumental post-1900 is not comparable in that high precision.

We thank the reviewer for raising this important point regarding the integration of sea-level data with different precisions. The statistical framework used in this study is an established method that has been applied to analyze modern instrumental together with geological data for multiple regions (e.g., Kopp et al., 2016; Kemp et al., 2018; Walker et al., 2022) with several procedures for handling this precision disparity. Specifically, this method transferred tide gauge data from monthly/yearly to decadal resolution to make it more comparable with high-resolution sea-level proxy data during the last 3000 years (many with sub-decadal and sub-decimeter uncertainty). These decadal to centennial signals are then explicitly captured by our fast-changing covariance function, which is separated from slow-changing geological processes (more uncertain due to sparse and noisy nature of geological proxies as the reviewer suggested). Therefore, our framework is capable of fusing data with different precisions into a common statistical framework while appropriately accounting for their respective uncertainties. A sentence has been added to the manuscript highlighting that instrumental and geological data have been integrated into a consistent framework (tracked change file lines 869-871). Our response to the reviewer's concerns about data quality is addressed in the next remark.

Kopp, R. E., et al. "Temperature-driven global sea-level variability in the Common Era." *Proceedings of the National Academy of Sciences* 113.11 (2016): E1434-E1441.

Kemp, A. C., et al. "Relative sea-level change in Newfoundland, Canada during the past~ 3000 years." *Quaternary Science Reviews* 201 (2018): 89-110.

Walker, Jennifer S., et al. "Timing of emergence of modern rates of sea-level rise by 1863." *Nature communications* 13.1 (2022): 966.

2.The Holocene sea level data used in this manuscript is mainly from literature, and many of their data was from quite old publications. For example, the review paper of Zong 2004 features sea level data collected from very old publications. Given the relatively undeveloped dating technology in the past, the previous C14 data had huge uncertainties or errors, including chronological errors/uncertainties of up to 200 years. Based on such chronological data, it is hard to obtain a reliable high-resolution (for example 100 years or less resolution), high-precision (for example millimeter- or even centimeter-scale) sea level profiles of the past. But the time-resolution of the post-1900 sea level data is up to annually or even monthly, so the sea level data between the geological centenary-scale sea level and the instrumental annual-to monthly-scale sea level are not comparable. Although the authors used some methods to correct their data for the purpose of comparability, the premise to do so must be that the raw data is reliable. Given the importance of this work, I strongly suggest the authors to re-do fieldwork (including the elevation measurements) and dating work so that they can obtain comparable data for this analysis.

We thank the reviewer for highlighting this potential issue regarding our sea-level data. As discussed in the previous remark, our framework can fuse data with different precisions into a common statistical framework. Therefore, our centennial global mean sea-level reconstruction is based on a high-resolution Common Era database since 3000 BP where ~98% of data has one sigma uncertainty <100 years, enabling us to reconstruct high-resolution signals.

We acknowledge that some data reported in Zong (2004) were collected from older publications; however, we want to emphasize that samples with large uncertainties are actually minimal in our database. For example, 48/88% of our data exhibit 1 sigma chronological uncertainty of <100/200 years, which is superior to a recent Nature Holocene sea-level paper by Hijma et al. (2025), where their major focus was developing high-resolution geological sea-level data, yet only 38/80% of their data achieved this resolution. Similarly, 76% of our sea-level data exhibit 1 sigma vertical uncertainty <0.5 m (which represents the overall mean uncertainty of a large Holocene sea-level database compiled in Khan et al., 2019), while data with large uncertainties (1 sigma uncertainty >1 m) account for only <10% of our database.

A standardization procedure following Hibbert et al. (2018) has been conducted to our database, which included elevation measurement uncertainty according to different types of coring equipment and recalibrated all C14 ages using the most up-to-date radiocarbon calibration curve while ensuring all sea-level indicative ranges are robust. Furthermore, our statistical framework explicitly propagates these data uncertainties into our statistical inference to ensure rigorous uncertainty quantification and probability reporting in our results.

We agree that additional fieldwork to reconstruct past sea-level change in China would be highly valuable. Indeed, some research projects are currently either proposed or underway, led by scientists within our author team, to better reconstruct sea-level change with improved spatiotemporal resolution across multiple Chinese regions. We also want to highlight that, given the Bayesian nature of our framework, our results provide not only mean RSL trends but also detailed spatiotemporal uncertainty maps for each sea-level budget component, which will provide valuable guidance for future fieldwork projects. We have added a sentence in the data availability section highlighting that all uncertainty estimates are available and archived in the Zenodo database and included uncertainty visualization in our sea-level budget Jupyter Notebook for better result communication.

Hijma, M. P., et al. "Global sea-level rise in the early Holocene revealed from North Sea peats." Nature 639.8055 (2025): 652-657.

Hibbert, F. D., et al. "A database of biological and geomorphological sea-level markers from the Last Glacial Maximum to present." Scientific data 5.1 (2018): 1-25.

Khan, N. S., et al. "Inception of a global atlas of sea levels since the Last Glacial Maximum." Quaternary Science Reviews 220 (2019): 359-371.

3.The main conclusion of this manuscript is mainly from the data of the last 4000 years, I don’t think its necessary to include the contents regarding pre-4000 years.

We agree with the reviewer that some of our major conclusions focus on the last 4000 years when GMSL and paleoenvironmental conditions became more stable for reconstruction. However, we believe including pre-4000-year data serves several important purposes. First, it provides essential context by demonstrating that modern sea-level budget changes are profoundly different from the last 4000 years of relatively stable GMSL and are instead similar to the middle Holocene when GMSL was the dominant driver. Second, the longer sea-level record helps us extract local geological and geomorphological controls on natural vertical land motion, which are critical for understanding baseline conditions before anthropogenic impacts and critical for informing future projections. Finally, this broader temporal perspective allows readers to better understand the magnitude of recent changes, particularly the substantial contribution of local anthropogenic-induced urban subsidence, within a comprehensive Holocene framework.

Referee #2 (Remarks to the Author):

I have just finished reading the manuscript by Yucheng Lin and co-authors about the 'Profound modern sea-level evolution against geological backdrop in southeastern China'. The authors present a thorough evaluation of sea-level changes in the region on multiple timescales, and taking into account several different processes. They conclude that early and mid-Holocene sea-level in SE China was largely controlled by deglacial land ice melt-induced GMSL, after which location-dependent regional processes became more important in the late Holocene. Over the past two centuries, accelerating GMSL rise became the dominant driver of sea-level change, locally enhanced by anthropogenic subsidence.

The manuscript is well written, and provides a comprehensive analysis of an extremely relevant topic. I am not very familiar with the technical specifics of both the hierarchical sea-level reconstructive model and the GIA models, so hopefully some other reviewers are. From what I can judge, the approach is very reasonable, and takes into account more processes (local, regional, linear, non-linear) than previous work. Several of the findings are not particularly surprising - like the importance of ice-melt induced GMSL in early and mid-Holocene, and the severity of anthropogenic subsidence - but I appreciate that the different spatiotemporal scales are translated into a common framework, and I do see the originality in that.

We appreciate the reviewer for recognizing the merit of this study. Our response for each remark can be found below, highlighted in blue.

To me the most surprising findings are the non-linear regional processes (Figure 3), showing dipoles in relative sea-level rates. If anything, one suggestion I would have is to discuss these processes in the modern-day setting or future projections. Can we use these findings to understand where internal climate variability might enhance or counteract GMSL-rise in the future? What climatic cycles drive the periodicity in the signal? Are those cycles affected by predicted/ongoing climate change?

We thank the reviewer for raising this important point. The key message we learned from our current analysis is that multi-decadal dipolar patterns, most likely related to internal climate variability, were observed in both the geological past and the modern era, and they have amplified regional sea-level rise and contributed to some significant flooding events that were geologically defined as once-in-a-millennium events. This robust evidence suggests that future sea-level projections should take such regional variability into consideration.

However, because internal climate variability results from complex and unpredictable interactions within and between climate system components—and considering that China's coastline is jointly influenced by ENSO, Pacific Decadal Oscillation, and the East Asian Monsoon—confidently identifying specific climatic cycles and periodicities driving these signals requires sophisticated analysis using ocean, climate, and coastal models (e.g., Dangendorf et al., 2023). While such comprehensive modeling represents a valuable avenue for future research, it is currently beyond the scope of our study.

Another perspective is that most current literature suggests that, even if we can fully understand the mechanisms that drive the observed regional pattern, it is almost impossible to reduce its uncertainty in future projections largely due to the chaotic nature of Earth systems (e.g., Becker et al., 2023 and Hu et al., 2018). We have now rewritten our final paragraph in the discussion section to present this challenge together with global mean sea-level rise and local urban subsidence in modern day and projecting settings. Collectively, we conclude that China is now facing a systematic challenge from global to local scales (tracked change file lines 432-445) and coastal management plan should be well informed local sea-level budgets.

Dangendorf, Sönke, et al. "Acceleration of US Southeast and Gulf coast sea-level rise amplified by internal climate variability." *Nature Communications* 14.1 (2023): 1935.

Becker, Melanie, M. Karpytchev, and A. Hu. "Increased exposure of coastal cities to sea-level rise due to internal climate variability." *Nature Climate Change* 13.4 (2023): 367-374.

Hu, Aixue, and Susan C. Bates. "Internal climate variability and projected future regional steric and dynamic sea level rise." *Nature communications* 9.1 (2018): 1068.

This is just a relatively minor remark for an otherwise impressive piece of work. To me there are no obvious flaws, and I enjoyed reading this manuscript. Below some minor language suggestions that I noted while reading.

Minor comments:

Line 23: findings

Adjusted.

Line 38: to do so

Revised.

Line 48: incorrect grammar, replace either ‘while’ or ‘which’

Amended as suggested.

Line 52: The Holocene

Updated as requested.

Line 56: Maybe mention that it’s not only a slowdown of sea-level rise, there is sea-level fall as well in many locations in Mid- and Late Holocene.

We thank the review for raising this valid point, we have change ‘sea-level rise’ to ‘GMSL rise’, which is more accurate here.

Line 61: complicated sentence, had to read a few times to understand

We thank the review for pointing this out. We have rewritten this sentence to make it more accessible (tracked change pdf lines 62-66)

Line 87: only uplift or also subsidence?

Here we want to state that, geological processes are the primary driver only for uplifting signal, while they also contribute to the subsidence signal, they are relatively minor compared to anthropogenic drivers.

Line 98: that consists

Adjusted.

Line 114: an additional

Modified as suggested.

Figure 1: what's the scale for the topography?

We used 'Natural Earth Relief' map for the land, which uses shaded relief to better visualize terrain (i.e., better visualize the geomorphological features like plain versus mountain areas), not a precise topographic scale. Its grayscale and color tints depict elevation changes for aesthetic clarity, not exact measurements.

Line 126: component

Amended.

Line 163: the Greenland

Changed as advised.

Line 193: I suppose these are cities within those provinces? Maybe specify

We have now included in province name in this sentence for clarity.

Figure 2: Why is a not before b in this figure?

We positioned subfigure (a), the spatial map, as the largest component of Figure 2 to clearly display detailed spatial information of reconstructed VLM and provide geographical context for the other subplots. We use (a) for this map to guide readers to begin with the spatial overview before examining the detailed analyses in subsequent panels.

Figure 2b: It's difficult to understand the difference between blue and purple shading (partly because of my colorblindness I suppose.. but still). And maybe mention that Mean, 68% and 95% CI is from This Study? It's clear from the caption, but would me more clear to also see that in the figure itself. And what are the light grey lines?

We thank the reviewer for raising this helpful point, we agree that making sure colors accessible for most readers is an important aspect. We have now made the purple color darker, which hopefully can make it more distinguishable from the blue lines. We have modified the legend as suggested. The definition of grey lines (additional 13 tested ice models) has now been added as well. We also changed the color in Figure 2b to different colors compared to c-h (except blue for GMSL, which indeed is the same for blue in c-h). Following this change, we have changed other figures and code to maintain the consistency of colors.

Figure 2a: It's a bit confusing that the legend for figures c-h is put here in Figure 2a, especially since the color scheme partially overlaps with the VLM-rate. And again, possibly because I'm colorblind, but I find it difficult to distinguish between the Regional non-linear and Local non-linear colors.

We thank the reviewer for this nice suggestion, we did not notice that we were using red and green together, which is not a good choice. We have now modified local non-linear to cyan, which should be a better choice. The legend location is made because Figure 2a has a large blank space to displace that legend, we have now added a clarification of that in caption.

Line 226: the Fujian coastline

Amended.

Line 260: the South China Sea

Adjusted.

Figure 3: maybe mark PRD and YRD on the map(s) as well

We have now marked PRD and YRD in Figure 3a as well.

Line 290: the Fuzhou Basin

Changed.

Line 298: the current

Added.

Line 341: use against or with, but not both

We have deleted 'with' in this sentence.

Line 356: remove an 'in'

Adjusted.

Line 431: diatoms, beachrock and mangroves

Updated.

Line 433: consists of

Refined.

Line 445: that consists of three hierarchical levels

Revised.

Line 451: RSL is modelled as

Updated.

Line 483: sun = sum

Corrected.

Line 485: the GP mean function

Added.

Line 491: The GIAGRD component

Revised.

Line 494: that cannot be captured by the GIA model

Refined.

Line 507: tide gauges

Amended.

Line 545: For the northwestern Pacific Basin, this reconstruction achieved

Changed.

Figure S5: but for specific cities

Updated.
Figure S7: Google Maps

Adjusted.
Line 902: result

Corrected.
Line 906: an accuracy estimate

Added.
Line 907: requires

Amended.
Line 913: no consideration of landscape evolution

Changed.
Line 918: remove one ‘of’
Adjusted.

Referee #3 (Remarks to the Author):

Dear Authors
I have now read and reviewed the manuscript and supplementary materials to my capacity.

SUMMARY, ORIGINALITY, SIGNIFICANCE, DATA, METHODOLOGY
This work is a ‘sea-level budget study’ of which the analysis newly extends to geological timescales of the last millennia, to be more complete in validating attribution insights regarding global mean sea-level (GMSL) rise since the 20th cy, making good use of geological relative sea-level (RSL) data sets from SE China, and bridging this with modern datasets (measurements, satellite data). Abstract and introduction paragraphs accentuate this by mentioning a post-1900 altered situation (modern times with Anthropocene sea-level rise), following up the situation of more or less stable GMSL of the last 4 ka (late Holocene times). The RSL data stretches further back, to 11 ka, when post-glacial GMSL was still significant, as were spatially diverse geophysical responses to it. The post-1900 coverage includes statements on coastal agglomeration urban subsidence and need for tailored adaptive strategies per location, fed with RSL and GMSL budget-analysis provided constituent insights. Indeed, this part of the global coastal ribbon is heavily populated and various drivers to land subsidence and water level rise combine and exacerbate adaptation challenges. Also urbanization is associated with waves of geotechnical investigation that in the long-run tend to increase geological data available and densify the amounts of sea-level data. There is thus economic-societal demand and stimulus and timeliness for integrative, budget constituent clarifying studies for the SE Chinese coastal ribbon, which this paper answers.

We thank the reviewer for recognizing the importance of this study. We also greatly appreciate the reviewer’s detailed comments and constructive feedback that significantly improve the quality of this paper. We have addressed all remarks in detail below, as highlighted in blue.

To execute the work, the authors make use of their ‘spatiotemporal hierarchical modeling framework’, developed in recent years (Lin et al. 2024; Walker et al. 2022). The application in this study is one of the first using this framework in full over Holocene time scales, the text includes a formal section describing the sea-level budget decomposition (2.1), the supplementary materials include the modelling code. Regarding the latter: I downloaded the packages, inspected data files and documentation, including the required software libraries etc., practical issues on my side prevented me to install the actual python/notebook package in time for returning this review, but I will email the editor once I have managed to do so. For the geological data: the data included are the extracted fields used in the metadata analysis and a cross-referencing to the source publications. The data files did not copy over and disclose the full geological data provided in the source publications, and the remarks on compaction or subsidence source in there: it is not reprocessed, uniformly screened geological data. This is defendable, as the methodology as input needs ‘raw RSL’ (label used in the China_RSL_data.xlsx supplement) anyway, because the subsequent analysis wants to attribute constituent terms by statistical processing starting from a raw total.

We thank the reviewer these helpful suggestions. We have now added all source paper references into our data file to solve cross-referencing problem. Data format in our last version follows previous sea-level budget studies (Walker et al., 2021; Kopp et al., 2016), with all data standardized following Hibbert et al. (2018). As reviewer noted, we use 'raw RSL' as input because our model aims to spatiotemporally attribute compaction

and tectonic processes rather than rely on site-specific corrections from individual studies. We have now included the 'original RSL' reported in original publication and the associated corrections for sediment compaction and tectonic as well.

Walker, J. S., et al. "Common Era sea-level budgets along the US Atlantic coast." Nature Communications 12.1 (2021): 1841.

Kopp, R. E., et al. "Temperature-driven global sea-level variability in the Common Era." Proceedings of the National Academy of Sciences 113.11 (2016): E1434-E1441.

Hibbert, F. D., et al. "A database of biological and geomorphological sea-level markers from the Last Glacial Maximum to present." Scientific data 5.1 (2018): 1-25.

The presentation of the respective processes considered in the Gaussian Process attribution, as well as the presentation of Results, begin with discussing forcings dominating the early and middle Holocene. The causal explanations for this period, rely strongly on the statistically optimized selection of GIAg(t) + GIAGRD(x, t) terms. When and where correct, the other terms in the Gaussian Process function then specify further budget contributions not captured in GMSL+GIA. When and where the GMSL+GIA optimization contains geophysical-modelling inherited bias (especially for early and middle Holocene, GIA modelling is susceptible for tradeoff effects), or data-density inherited bias (unclear if the smaller density of data for the early millennia is compensated for in the numeric selection of GIA results), the other gaussian 'kernels' will compensate that bias, by over-specifying. On this part of the paper, I have some critical remarks (see below).

We appreciate the reviewer to notice this question and our response can be found under major remarks below.

The paper contains a meaningful discussion of climatic, centennial variable GMSL variations (g(t) term) during the late Holocene (results: lines 245-261; causation: lines 262-272). I think (owing to explicit constraining of timescales between 'kernels' of the Gaussian Process operationalization) that these results for the late Holocene will be independent from my concerns of GIAGRD and millennial-VLM attributions above. I have smaller remarks on this section only. The text and an inset in Fig 2A for the late Holocene, cover the more data-dense and stronger constrained part of the GMSL fitting and here I find the argumentation for credibility sufficient. I regard (as do the authors, I reckon) the main point of the paper to be to compare late Holocene to post-1900 (which abstract and introduction home into, and which is played out strongly with Figure 3 and 4).

We thank the reviewer for acknowledging our efforts on climatic impact on sea-level change and centennial GMSL variation.

The paper covers a great stretch of SE Chinese coast, with the Yangtze delta region in the north (Shanghai), and a gently uplifting coastal section to the south, dented with further substantial estuaries/deltas, including the Pearl river delta (Hong Kong), which is to be commended. The paper ends with a condensed confrontation and discussion (Lines 355-405) of vertical land movement (VLM) and the anthropogenically enhanced rates of that in deltaic regions being larger than the GMSL, importantly quantifying both these numbers. There is merit in combining first the topic of Holocene RSL and then that of actual sea-level rise and land subsidence, but the paper also changes topic a bit in this last section. It is a strong application example, but perhaps it takes too much space. I think Table 1 is not perse needed in main text. I wonder if the closure of the paper would not be stronger by e.g. comparing and contrasting attribution performance and antropogenic subsidence

evaluations for Pearl delta, stretch in between, Yangtze Delta: brought more topical/less generic than the current final discussion paragraphs.

We thank the reviewer for this helpful suggestion. While providing attribution performance comparisons across different deltaic regions would strengthen our analysis, the InSAR-based VLM data by Ao et al., (2024) are currently available only as point estimates for each city (with mean, 5th and 95th spatial percentiles), which precludes systematic spatial comparison.

Nevertheless, comparing point estimates between our reconstructed natural VLM and InSAR-derived total VLM reveals first-order agreement for cities with the highest rates of natural subsidence and modern InSAR subsidence (particularly Chaozhou, Shantou, and Hangzhou, marked in bold in Table 1). This suggests that underlying geological and geomorphological features influence modern subsidence patterns, providing a promising direction for future research to better separate natural and anthropogenic contributions across different deltaic settings. We therefore recommend placing Table 1 in the main text, as it provides quantitative values directly supporting our key finding regarding the distinction between natural and anthropogenic VLM rates across different cities, which is central to our conclusions.

We have also rewritten the final paragraph of the modern sea-level budget section. Drawing on the major findings from analyzing both geological and instrumental data, we conclude that southeastern China now faces systematic challenges from sea-level rise from global to local scales, which should be considered in risk management planning—a key message this paper aims to convey (see track change pdf lines 432-445).

Ao, Z., et al. "A national-scale assessment of land subsidence in China’s major cities." Science 384.6693 (2024): 301-306.

By the nature of the analysis: Yes, the use of statistics is appropriate, and includes formal uncertainty treatments. The text and figures display the uncertainties. The discussion of the results makes relatively little use of these uncertainties, to underpin interpretations/argumentations.
I regard conclusions robust, valid and reliable for the late Holocene and modern times, but have some concerns regarding the tradeoffs between GMSL, GIAg GIAGRD and VLM for the early and middle Holocene. See below for the major remark including suggested improvements. See below for further minor remarks.
References: no remarks.
Clarity and Content: overall fine. Minor queries.

Major remark: early and middle Holocene attribution performance

I feel the text on early and middle Holocene, and with that on GMSL, GIAg and GIAGRD respective attributions need more nuance: i.e. better textual appreciation of statistical, modeling and physical reality tradeoffs embedded in our schematizations, concepts and data collection. This is important for realism claims on the statistically-structured attribution for the early to middle Holocene (method allows good effort, but results not yet validated), as it does for the late Holocene and the modern time frames (method+data allows for very good effort, results indeed validated).

We thank the reviewer for this helpful suggestion, our response to each major remark can be found below.

A) Detailed coverage of the early to middle Holocene results starts at line 169-175. This discusses the span of

early-middle Holocene GMSL: relatively less (e.g. Creel et al. 2024 and Peltier et al. 2015: some 40 odd meters) or relatively more (Bradley et al. 2016 and Lambeck et al. 2014: some 50 odd meters; Fig. 2b). The latter position is in-line with the statistical attribution outcome of this study (also shown in Fig 2b), although it is noted that resolving the GMSL span is not the scope of the study. If there is an upper and a lower estimate, and a new study presents a third result that is close to one and less close to the other, on itself confirmation of causal correctness of the GMSL fitting. I feel more causal argument should be added to this part of the text, and just the current statement in line 182-184 'underscores its reliability for delineating GMSL trend for China' is not enough (and challengeable, as conceptually there cannot be a derived 'GMSL trend for China' that is in mismatch with that for the rest of the globe).

We thank the reviewer for this important clarification. The reviewer is correct that our phrasing about 'GMSL trend for China' is conceptually problematic—GMSL should be globally consistent and cannot have regional variations. What we intended to convey is that our statistical approach, informed by Chinese RSL data, provides reasonable fit to other data outside China, so the reconstruction should provide a decent boundary condition for China to investigate other processes. However, we acknowledge this was poorly articulated.

Regarding early-middle Holocene GMSL estimates, our results align more closely with ANU and Bradley et al. studies, which predominantly use far-field data from Asia, Australia, Pacific, and Indian Ocean regions. These far-field locations are generally considered optimal for GMSL reconstruction as they experience minor ice-water-loading GRD effects. In contrast, ICE6G_C and Creel et al. rely more heavily on North American and European datasets (i.e., near or/and intermediate field that subject to larger ice-loading GRD effects), which are not only more sensitive to mantle viscosity uncertainties but also depend critically on assumptions about ice sheet geometry, melting chronology since the glacial period, and spatial distribution of deglaciation (especially on early Holocene). While Creel et al. attempted to address some of these issues through pseudo-lateral varying Earth models (i.e., calibrating 1D Earth model to each smaller region), substantial uncertainties remain regarding the ice models, especially for North American and Euraisan Ice Sheets, most of which were developed assuming a single1D Earth rheology.

However, a detailed discussion of these paleo ice sheet and GIA modeling complexities would be overly technical for many readers without specialized paleoclimate backgrounds, who we anticipate comprise the majority of the readers for this paper. More importantly, resolving early Holocene GMSL is not the primary focus of our study, and significant uncertainties remain across all approaches in this time period. We therefore removed the statement about 'reliability for delineating GMSL trend for China' and instead acknowledged that early Holocene GMSL remains an open research question (tracked change pdf lines 198-208).

Creel, R. C., et al. "Global mean sea level likely higher than present during the holocene." Nature Communications 15.1 (2024): 10731.

B) The data density for the middle to late Holocene is considerably larger than that for the early to middle Holocene (understandably). In the statistical fitting and cross-validation test of the Gaussian Process model (inspected through Figure S4a in the supplementary material), I suspect that it optimizes performance for younger time, especially in the process described in line 128-133. It appears that the 'other GPs' compensate for underfitting of the GIA GP(s) in this domain. I wonder if some weighted optimizing could have been considered that would change the relative importance of GIA and other processes in the early to middle Holocene in Fig. S4a.

We thank the reviewer for this suggestion. The original manuscript lacked clarity regarding the use of sea-level data outside China during early to middle Holocene. These data were not used to optimize our GIA model (our model is tuned solely to the China database). Instead, they served as an independent test to

demonstrate that our China-tuned GIA model ensemble can reasonably approximate unseen sea-level data outside China. We have modified the manuscript to clarify it (tracked change pdf lines 859-861).

For our China sea-level database (Fig. 1c), data density is quite comparable across early (99 data), middle (131 data), and late (80 data) Holocene, with higher density in early to middle Holocene mostly due to extensive inland archaeological drilling. To quantify this slight data density variations in our results, we applied weighting factors of 0.33 (early), 0.25 (middle) and 0.41 (late), accordingly. This results in minimal changes to the posterior GMSL estimate (maximum difference of 0.17 m at 9000 BP). Similarly, posterior estimates for lithosphere thickness merely shifted from N(85.4, 12.4) to N(86.2, 12.2) km, upper mantle viscosity from N(0.19, 0.22) to N(0.16, 0.2) × 10^{21} Pa s, and lower mantle viscosity from N(1.69, 0.95) to N(1.59, 0.91) × 10^{21} Pa s. These suggest that temporal data density does not compromise the robustness of our results.

C) The text and Fig 2b are used first to discuss the early-middle Holocene (GMSL result unlike Creel et al. 2024), and then the inset to discuss the late Holocene (GMSL result similar to Creel et al. 2024; GMSL high stand just above 0). For the early and middle Holocene, the statistically attributed GMSL result better matches the relatively larger GMSL span. It could be, that a larger long-term superregional subsidence signal (not well captured in global GIA modelling because of uniform rheology choices in e.g. the Peltier modelling) is misattributed to be a part of GMSL signal. I note that the Lambeck et al. 2014 study, explicitly dropped potential datapoints from e.g. the Yangtze delta (fairly dominant in early-middle Holocene domain of your dataset) as for their analysis at the time, the hard to specify extra subsidence in play in the megadelta setting. As I understand it, in case your GMSL would have been higher positioned (because… different rheology preferred, different numeric scheme preferred), this would be a tradeoff between GIAg(t) (selected to plot in Fig. 2b; a bit smaller) and GIAGRD(x,t) (a bit larger where subsiding, a bit smaller too, where uplifting), and/or the r(x,t) term modeled later on in the analysis could gain further importance in the early and middle Holocene (i.e. bigger piece of the pie in Figure 3 time series charts). Because the budget attribution chain starts with the GMSL inference from GIA modelling fitting on raw RSL data, it appears it does not get that chance. It could be, that the GIAGRD(x,t) term (continental levering, and complications in regions receiving big volumes of sediment in addition to hydrostatical loading) in physical reality is not as smooth (your statement in line 188-189) as sampled from GIA models (that have coarse spatial grids, and no consideration of crustal make up differences and exact locations of flexure) + there is potential bias in GIAGRD values surrounding megadeltas because of the land-sea masking in GIA models being based on modern topo/bathymetry (not corrected for Holocene burial thickness).

We thank the reviewer for these insightful comments. We discuss the attribution and GIAGRD(x,t) and GIAg(t) trade-off here, with sediment-induced landscape evolution impacts discussed in response to major remark D.

The misattribution concern is valid; however, our GIA model ensemble incorporates data from multiple sources that should prevent systematic bias toward superregional subsidence regions. Specifically, our ensemble uses middle-late Holocene data evenly spread across China and utilizes early Holocene data from outside the Yangtze delta as well (comprising ~30% of early Holocene data points).

Several lines of evidence support our attribution approach:

- Our GMSL reconstruction aligns well with ANU and Bradley et al. (2016) reconstructions. The former explicitly excludes megadelta samples while the latter included South Asian data, providing independent verification of our early Holocene GMSL estimates. If we misattribute the GMSL to

superregional subsidence regions, our GMSL results should be substantially lower than those two studies.

- Our China-tuned GIA ensemble fits data outside China well (as noted in major remark B), suggesting our approach captures broader regional patterns rather than local biases.
- If GMSL were positioned higher in the early Holocene (as suggested by ICE6G_C or Creel et al., 2024), the slight uplift signals in Guangdong and Hainan would shift to subsidence (Figure 2a). However, independent geological evidence—including geological surveys and Marine Isotope Stage 5 marine terraces—indicates uplift in most of coastal Fujian, Guangdong and Hainan, supporting our current reconstruction.

Our statistical formulation explicitly incorporates GIAGRD and GIAg(t) trade-offs through the GP mean function's covariance structure. By testing a broad range of plausible ice and earth models, RSL covariance from these ensembles (i.e., GIAGRD and GIAg(t) trade offs) is embedded in our covariance function. The interpretation of r(x,t) terms before 5000 BP remains challenging due to superimposed signals with similar spatiotemporal scales (regional tidal evolution, sedimentation changes during fluvial-to-marine transitions, and land-ocean mask misclassification; see more details in next remark), which is acknowledged in tracked change file lines 278-279.

Regarding sediment-induced sea-level change that may not be as smooth as GIA models predict, there are two signals for sediment-induced sea-level change that are expected in the Yangtze delta. Firstly, sediment-related isostatic adjustment (i.e., sediment isostatic adjustment; SIA) shares the same physics as GIA (see, for example, Lin et al., 2023, for reconstructed SIA signal for the Great Barrier Reef) to deform the underlying lithosphere and mantle. As we show in our model calibration, China's lithospheric thickness has a posterior distribution of N(85.4, 12.4) km, and our GIA model spatial resolution is 70-80 km, which should be sufficient to capture the flexural signal caused by sediment/hydro-isostatic loading. Secondly, sediments could induce localized subsidence through compaction, which our locally varying linear term is designed to capture.

Lin, Y., et al. "Relative sea level response to mixed carbonate-siliciclastic sediment loading along the Great Barrier Reef margin." *Earth and Planetary Science Letters* 607 (2023): 118066.

D) The results for the Yangtze megadelta, I suspect to be affected by my early-middle Holocene bias concerns. The Results section for the Yangtze delta summarizes: GIAGRD predicts uplift, RSL data points lay deep, local to regional subsidence term (VLM) is to be regarded large, and of opposite sign as GIAGRD (Taizhou, Fig. 2c), but it may be that the GIAGRD-uplift prediction is misplaced in the GIA model, as it masks land and sea with the modern delta topography. But with some reconsideration of bathymetry at the time of postglacial transgression, GIAGRD at Taizhou could be near-zero, and the predicted subsidence term smaller. GIA-modelling groups are working on that in next generation GIA modelling, but GIA runs over bathymetries undone of Holocene megadeltaic sedimentation are not yet available. The authors used current generation GIA modelling, that have 'bias' or 'artefact' imprints in megadelta areas. This is not a method flaw, but a method-input caveat, that I think needs to be more openly addressed in the presentation of results and discussion.

We thank the reviewer for raising this important point regarding land masking effects in megadeltas sea-level calculation. The Yangtze region exemplifies this challenge, as assuming modern land masks can overestimate GIAGRD signals at sites like Taizhou that would have been submerged during transgression prior to middle-late Holocene sediment accumulation.

While developing a new GIA model with time-evolving topography is beyond our current scope (as reviewer notes), we conducted a demonstrative test to conservatively quantify misfit from ocean-land mask misclassification (details provided in tracked change file line 935-964). Using the sediment drilling age-depth

database from Wang et al. (2018), we reconstructed Yangtze delta sediment accumulation depths at 8500 BP (when most YRD sea-level data formed) through a similar spatiotemporal Gaussian Process method used in this study. We then subtracted this thickness from present-day topography to approximate paleo-topography and reran our best-fitting GIA model (with 96 km lithosphere, 0.05 and 1 x 10^{21} Pa s upper and lower mantle) with this reconstructed topography (see Extended Data Fig. 3). This approach provides conservative bounds on GIAGRD error, with true values likely falling between modern and paleo-topography scenarios (earlier land formation results in GIAGRD signals more similar to those using modern topography, and vice versa).

Results indicate that:

- Paleo-topography reduces GIAGRD signals at Taizhou by approximately 20% during early Holocene (from ~10 to ~8 m in ~11,000 BP). And this difference decreased to near-zero between 6000-5000 BP (Extended Data Fig. 3i).
- The misclassification effect becomes minimal between 6000 and 5000 BP, leaving late Holocene interpretations unaffected, largely because regional GRD in the Yangtze delta is governed by broader-scale uplift signals related to continental shelf inundation, which produce nearly identical signals in both experiments. The spatiotemporal pattern of this correction resembles other signals captured in our r(x,t) term, therefore r(x,t) could reflect part of this signal in early-middle Holocene.
- We can further quantify the conservative rate of ice-ocean mask misclassification impact on VLM calculation through (GIAGRD_modern_topo(x,t) – GIAGRD_paleo_topo(x,t))/t, where t is the RSL sample age and x is sample location. Applying this correction term (mostly <0.2 mm/yr) to our VLM reconstruction indicates the overall subsidence pattern in the YRD remains robust (Extended Data Fig. 3l).

We have expanded the GIA modeling section to openly discuss these considerations and acknowledge this in our main text (tracked change file lines 247-251).

Wang, Z., et al. "Three-dimensional evolution of the Yangtze River mouth, China during the Holocene: impacts of sea level, climate and human activity." *Earth-Science Reviews* 185 (2018): 938-955.

Minor remarks (part redactional, part scientific)
Line 52: Holocene --> The Holocene
Adjusted.

Line 55: early to mid Holocene --> early to middle Holocene
Similarly: Line 56, 119 (Fig. 1; Caption), 170, 175, 189, 199, 201, 232, 246, 330, 359 (Fig. 4)
(mid Holocene is short speak, not official period naming, middle Holocene is better)
(Nature tends not to capitalize early/middle/late in front of geological period names)
We thank the reviewer for this insightful suggestion. We have changed mid Holocene to middle Holocene throughout our manuscript.

Line 70-72: I agree with the statement, but wonder: would the recent North Sea study of Hijma et al. (2025, Nature) categorize as a long-term sea-level budget study outside North America + Caribbean? It has aspects of it, although it focuses on early and middle Holocene macro constituents and is not explicit on the further constituents in play in late Holocene and post-1900.

We thank the reviewer for highlighting the relevant study by Hijma et al. (2025), which was not available during our submission. This paper mainly focused on providing new sea-level data for the North Sea, focusing on constraining global mean sea level and GIA modeling with limited discussion of other budget components, it does not systematically quantify spatiotemporal signals beyond GMSL and GRD. Therefore, it should not affect our statement here.

Line 146-147 gives a general statement, where the authors say that by specifying the GIA terms from the output of geophysical modelling, physical interpretation credit transfers to the statistical method. This is somewhat true, but I would stress that this only goes for the physics considered in the models. I see this statement as rhetoric, not full scientific justification. What is kept out of GIA models for reasons, for good parts is also physical. GIA modelling focuses on physical processes dominant in late glacial, early and middle Holocene, that level out or become background in the late Holocene (where your main use case is). In fact, you specified further processes to capture the physical reality of the Earth better, to add to the GIA-modelling selected physical processes, that become dominant in late Holocene and modern times. So it is for the inclusion of those processes that I would like to see arguments.

We thank the reviewer this this valuable feedback. In response, we revised the manuscript to clarify that the GIA ensemble enhances physical interpretability, particularly for early Holocene dynamics, compared to purely statistical models. We also added an argument highlighting that our GP model captures physically meaningful late Holocene signals, like GMSL fluctuations and regional climate variability (tracked change file lines 861-887).

Line 151-153: the sentence is written/can be read as if it is a surprise outcome, but this was the expectation/established insight: soon as you cover the full Holocene, of course melting of remnant ice-sheets of Last Glacial is your prime driver. I suggest to rewrite, incorporating the word ‘as expected’.

We have added a statement ‘as suggested by previous study’ to clarify this (tracked change file line 172).

Line 160: ‘ By 6000 BP, the demise of the North American and Scandinavian Ice Sheets’ --> remove ‘ and Scandinavian’ (those were gone by 10000 BP already, 6000 BP relates to North America (minus Greenland) predominantly, if not solely).

Modified accordingly.

Line 198: ‘ suggests a continuous sea-level fall […] during the mid- to late-Holocene’ --> ‘suggests a continuous sea-level fall […] during the late Holocene’ . The exact onset/turn over moment from rise to fall: perhaps 5.5 or 5 ka at the very end of the middle Holocene, but onset timing is trivial here. Your sentence is about the down going trend in the late Holocene primarily.

We thank the reviewer for this suggestion. We noticed that this sentence mentioned mid Holocene highstand a common term that some readers might be interested, so we suggest keeping middle Holocene here.

Line 201: ‘However, the sea-level database reveals more complex patterns.’ --> ‘ However, the sea-level database reveals more complex patterns, attributable to land movement’ (redactionally, I advice to give reader the cause beforehand, then exemplify). Also: for the big delta’s, this is an established expectation, see my note on Lambeck et al. (2014) mentioning it as a reason for exclusion there).

We have modified this sentence accordingly. Our aim here is to present the spatial variability revealed by the sea-level data in a neutral manner, without implying whether it is expected or unexpected.

Line 209-215: for clarity repeat at the begin of this results, that the model attributes this in statistically-optimized tradeoff with the GIAGRD(x,t) term.

We have added the following clarifications (tracked change file lines 249-251):

“In megadelta systems like the YRD, Holocene sediment discharge significantly altered the land-ocean boundary, a process not captured in our GRD calculation. While this may cause overestimate in reconstructed subsidence rates by up to ~0.2 mm/yr, the overall subsidence pattern in the YRD remain robust (see Methods)”

Line 219-218: perhaps rephrase: “The GP model thus is capable to identify the areas where there is extra subsidence due to tectonics and sedimentary loading.’

We have changed this sentence accordingly (tracked change file lines 245-246):

“These findings align with our long-term subsidence signals, confirming the model ability to identify to regions where there is extra subsidence due to sedimentary processes. In such regions, high subsidence rates can surpass GRD-induced uplift, explaining the observed continuous rising sea-level trend (Fig. 2 c, d, and f).”

Line 274: “paleo-megafloods” --> “geologically-identified sizeable palaeoflood events”. I recommend to avoid periodically calling recurring, very large floods ‘megafloods’. Reserve that term for really crazy floods owing to glacial (mega)lake , (mega)outburst). I think in paleoflood science, there is some inflation in the use of the prefix ‘mega’. I do agree with the notion of ‘dipole pattern’ and the summary of the literature on this.

We have replaced all instances of “paleo-megaflood” with “paleo flood” throughout the manuscript. We want to avoid introducing overly specialized terminology, as the intended audience may not have the background necessary for such distinctions.

Line 280-281: double check if you are citing radiocarbon years BP or calibrated radiocarbon years BP in this particular instance.

We have double checked the original publication and can confirm the reporting age here is calibrated radiocarbon age instead of conventional radiocarbon age.

Line 295-297: You cite three neolithic references (probably from neolithic inhabitance in the drainage basin of the rivers), but not the papers of Wang et al. 2012 and Wang et al. 2018, that you drew the sea-level data from in the supplement in the deltaic/coastal regions proper. I suggest to add these here, and use it in a way connecting back to the data explicitly used to start this. Furthermore: this Neolithic period was essentially before 5000 years ago. The period from about 5000 years ago, that you were discussing above, would match the Bronze Age (and younger archaeological periods) (the transition of Neolithic to Bronze Age is around 5000 years ago).

We thank the reviewer for this suggestion. We have cited Wang et al., 2012 and Wang et al., 2018 now and added Bronze age in our statement.

An interesting point is also raised about the transition between Neolithic and Bronze age transition period. Although transition of Neolithic to Bronze Age is generally around 5000 BP in Western Asia and part of Europe, Chinese archaeologists generally use Bronze age from the Erlitou Culture (3800 -3500 BP; see, for example, Campbell, 2014), marked by large-scale bronze use. Therefore, one of the most famous hydrological systems built by the Liangzhu culture (~5500-4200 BP) is generally referred as a Neolithic culture in China.

Campbell, R. B. (2014). Archaeology of the Chinese bronze age: From Erlitou to Anyang. The Cotsen Institute of Archaeology Press.

Line 297-299: The sentence reads: "However, due to significant noise in RSL proxies and the difficulty of modeling abrupt events (e.g., seismic) with GP, distinguishing meaningful local signals from current China RSL database remains challenging.". I think this statement can be bend into something stronger: the whole premise of attributing and sea-level budgeting is that the full sum of terms is to be attributed, and each term as precise as possible. In principle, no noise should remain (the practical formulation of Equation 2 does not mention it; other more theoretical formulations of spatiotemporal attribution do: there is then a closing term that is a noise term – as you do in Eq. 3/Eq. 6 with the omega term). Can't you extract from the analysis results, how much unexplained noise remains, and what greater part is model-explained? It appears you do so with the Eq.8-10 in method section, but the aspect of uncertainty in attribution did not make the Results and Discussion section.

We thank the reviewer for this comment. To clarify our approach, Eq. 2 describes the latent RSL process (i.e., true RSL process without noise), while Eq. 3 represents our data level model that comprehensively accounts for multiple uncertainty sources in RSL proxy including RSL reconstruction uncertainty (ε), additional white noise for unmodeled variability (ω), site-specific datum offsets (γ_0), and potential compaction uncertainty for intertidal data (γ). This separation allows us to distinguish the process level (modeling uncertainty) from data level uncertainties. The process, data and parameter levels uncertainties were fully propagated through Bayesian inference and included in all reporting results (including probability and uncertainty estimates).

Regarding the statement local signal attribution: our analysis shows that local signals (up to ~0.3 m magnitude) are not statistically meaningful because these signals are substantially smaller than the reconstructed uncertainty for locally non-linear signal (most of reconstructed uncertainty is ten times larger than the reconstructed mean value). This represents a data limitation due to noisy nature of geological proxy rather than a methodological one.

Line 220-222: surpass the GIAGRD uplift --> this remark triggered me. For the large delta plains that are sinking, it may well be that 'because GIA modelling uses modern day land-sea boundary for the land-sea masking' it considers deltaic areas land through out the Holocene, and misplaces the continental-levering hinge location. It may be that the GIAGRD term is off (hinge position: what is small uplift in coastward most cells could even be small subsidence), and that the subsidence term compensates. This then leads to overattributing subsidence to tectonic and sedimentary loading causes. Especially for the Yangtze delta I would be cautious. For the other deltas, because of the topography surrounding their inner reaches, the potential for GIAGRD mis-prediction and VLM overcompensation may be less worrisome.

We have discussed this concern in our response to remark D above. Although the GIAGRD term can be overestimated due to land-ocean mask misclassification, the overall subsidence pattern in the YRD remains robust.

Line 318-319: "China's modern sea-level budget compared to earlier Holocene stages."
--> "China's modern sea-level budget compared to that of the late Holocene." I recommend to mainly contrast late Holocene to post-1900. Rates of GMSL change in the early to middle Holocene are comparable to post-1900, only late Holocene is different.

Adjusted as recommended.

Line 340-354: For the VLM-quantified uplifting areas, I agree that GNSS helps validating.
For the VLM-quantified subsiding areas, I suspect overestimation of the subsidence rates (because of GIAGRD and tradeoff issues), and perhaps misattribution of the GNSS station rates as fully natural instead of part natural/part anthropogenic. The argument 'the consistency underscores' does not hold when the results are 'right for the wrong reasons'.

We thank the reviewer for this important point. Our analysis shows that natural subsidence contributes minimally to observed GNSS subsidence rates, which are substantially higher (typically order-of-magnitude) than our natural subsidence estimates. In this case, misclassification of the land-ocean mask should not impact this result. Also, as discussed in major remark D above, misclassification of the land-ocean mask should not significantly alter the spatial pattern of our VLM reconstruction, and the overall consistency between the spatial patterns of our VLM reconstruction and the modern product by Oelsmann et al. (2024) should support the validity of our VLM reconstruction.

Line 408-410: for the late Holocene and modern times, I agree that there has been validated. For the early and middle Holocene, I see the results mainly as documenting GIA-modelling performance against RSL-data, and as a spin up to allow careful analysis of the Late Holocene. The budget attributions proposed here (specifically for GIAg, GIAGRD and VLM, starting from GMSL), are regionally optimized but remain yet to be validated i.m.o.

We have modified in the main text to highlight the potential problem of GIAg(t), GIAGRD and VLM in early Holocene. Line in tracked change file lines 247-251. Therefore, readers should be aware that our major focus should be middle-late Holocene and modern time periods where our results are validated.

Line 456, 462: "γ is multiplied by y so a deeper sample is prone to have larger compaction uncertainty

(Engelhart et al., 2015)."
There may be a technical misspecification of this property: It is not perse so that deepest intercalated samples are the strongest vertically displaced: samples in the middle of the Holocene column, have the largest potential lowering owing to 'autocompaction' processes. Towards the base the compaction is stronger, but the vertical lowering of a sample is not solely the compaction of the sample itself, but also of all material between incompressible substrate and sample.

We thank the reviewer to raise this valid point. We acknowledge that this formulization is a highly simplified term, which is now stated in our manuscript. It should be noted that, our γ term is only adding additional uncertainty to deeper sample, instead of attributing more compaction magnitude for deeper sample, which should be appropriate in first order.

Line 442-515: My overall compliments on the readability of technical section 4.2.

We thank the reviewer for this compliment for our efforts to make this research more approachable.

Line 516-537: You may want to mention the land-sea mask used and typical spatial resolution (tessellation, harmonics) of the GIA models.

We have now added this information in our manuscript (tracked change file lines 917 and 926).

Referee #3 (Remarks on code availability):

I have downloaded and reviewed the data files. They are clear.
I have downloaded but at time of submission not the reviewed code,
and for logistic reasons at my side cannot do so before passing of review deadline.

There may be follow up, in communication with the editor. From the contents of the repository, I expect to be able to install and open and inspect the notebooks, but more as an occassional code user than as a regular scientific coder myself.

We thank the reviewer for recognizing our efforts to make our model, data and results more accessible.

Referee #4 (Remarks to the Author):

Summary

-In the manuscript titled " Profound modern sea-level evolution against geological backdrop in southeastern China", Lin et al. compile and document the sea-level budget for southeastern China since the Holocene. This is achieved through the compilation of a Holocene sea level indicator database, use of a spatiotemporal hierarchical model that combines several modeling approaches from previously published sources and glacial isostatic adjustment. Results were then placed in a modern context through calculating the modern sea-level budget and associated contributions to vertical land motion. The authors found that, up until the Pre-Industrial, sea-level rise in southeastern China was dominated by global mean sea level rise. Following a period of stability, sea level rise accelerated more than ten-fold by the 19th century, primarily driven by anthropogenic subsidence. This paper and accompanying model will compliment existing Holocene-to-modern regional sea level analyses as well as provide useful frameworks for highlighting modern sea-level rise related hazards. The work is of interest to the the broader sea level community and I recommend publication after minor revisions.

We thank the reviewer for the recognition of this study's importance. Our response to each remark can be found below, highlighted in blue.

Main Comments (Manuscript)

-This manuscript takes a comprehensive approach in order to disentangle contributors to China's holocene and modern relative sea-level rise. However this same comprehensive nature, often times becomes pedantic, leading to a rather cumbersome reading experience. My suggestion would be to streamline the results and discussion section, keeping the explanation of methods to an absolute minimum in order to focus on results and implications therein. Furthermore, the reference to coastal hazards related to sea level rise—the reason this study is important—is limited and often reads as an after-thought. I believe after streamlining the "Results and Discussion" section and addressing occurrences of needlessly long sentences, this manuscript is ready for publication.

We thank the reviewer for this helpful suggestion; we have modified our manuscript and moved all methodological details into the method section to avoid overly cumbersome reading experience. Accordingly, we have modified our manuscript to add some information to help readers better understand the concepts in our budget explanation sections.

We have also rewritten the final paragraph of the modern sea-level budget section. Drawing on the major findings from analyzing both geological and instrumental data, we conclude that southeastern China now faces systematic challenges from sea-level rise from global to local scales, which should be considered in risk management planning—a key message this paper aims to convey for modern and future coastal hazards (see track change pdf lines 432-445).

Referee #4 (Remarks on code availability):

-The code is easily accessed and installed thanks to a clearly written README file. The code repository consists of a main python based code that employs the PaleoSTeHM framework to calculate RSL along

China’s southeastern coast. Also included are 5 Juypter Notbooks, which are all easy to follow and reproduce the figures included in the manuscript.

We thank the reviewer for recognizing our efforts to make our model, data and results more accessible.

Referee #5 (Remarks to the Author):

Dear Editor,

I have read with interest manuscript "Profound modern sea-level evolution against geological backdrop in southeastern China" by Lin et al., submitted to Nature. The paper reports evidence for recent, significant modification in the Holocene geologic regime of sea-level change in China, which points to an acceleration that is exacerbated by anthropogenic activities. The work is based on a robust statistical methodology and it is well presented. Although the results appear to be significant, it is difficult to put this work in the perspective of previous studies, since its novelty (if any) is not sufficiently highlighted, in my opinion.

We thank the reviewer for recognizing the robustness of our method and results. We have now added a statement about southeastern China's immense socioeconomic importance to our summary paragraph, highlighting that this important region has not been systematically analyzed before. Another key advantage of this study is that we use a large amount of paleo-environmental data to validate our budget reconstruction, which is also comparable to modern measurements (e.g., ocean dynamic patterns and GNSS), providing critical and robust information to consider in future projections.

We have also rewritten the final paragraph of the modern sea-level budget section. Drawing on the major findings from analyzing both geological and instrumental data, we conclude that southeastern China now faces systematic challenges from sea-level rise from global to local scales, which should be considered in risk management planning—a key message this paper aims to convey, which should further highlighting the significance of this study (see track change pdf lines 432-445).

Furthermore, some of the sea-level "components" considered in this work suffer from significant limitations. GIA is modeled through traditional 1D models, which could be OK also in view of the significant uncertainties existing, but this would al least require some discussion. Also, the set of models chosen to construct the GIA ensemble appears to be quite ad-hoc.

We thank the reviewer for noting the limitations of 1D GIA models, which were not sufficiently discussed in our original manuscript. We have revised the manuscript (tracked change file lines 926-934) to justify using an ensemble of 1D GIA models over a 3D model for this study, emphasizing their suitability for our study:

1. Prior seismic tomography-based models (e.g., Austermann et al., 2021) indicate minimal lateral variations in lithospheric thickness and upper mantle viscosity in southeastern China, with notable variations only below ~500 km, which minimally affect sea-level changes in far-field regions like China.
2. Calibrating our GIA model ensemble to southern (15–25°N) and northern (25–35°N) China sea-level data favors similar Earth parameters
3. The lower computational cost of 1D GIA models enables comprehensive sampling of ice and solid Earth model parameter uncertainties, the primary uncertainty source for China.

We also clarified the GIA model ensemble selection. The earth model selection guided by prior China-based GIA modeling (Bradley et al., 2016) and seismic tomography reconstructions (Austermann et al., 2021). The selected ice models aim to bridge the uncertainty gap between two most commonly-used ice models: ANU and ICE6G_C. ICE6G_C provides a reasonable upper bound, melting 1–2 ka earlier than observed in Asia, Australia, and Egypt, while ANU offers a lower bound, assuming a larger Antarctic Ice Sheet (25–30 m sea-level equivalent above present during the Last Glacial Maximum), which recent field-based and dynamical ice modeling studies suggest is likely overestimated (tracked change file lines 921-923).

Bradley, S. L., et al. "Modelling sea level data from China and Malay-Thailand to estimate Holocene ice-volume equivalent sea level change." *Quaternary Science Reviews* 137 (2016): 54-68.

Austermann, Jacqueline, et al. "The effect of lateral variations in Earth structure on Last Interglacial sea level." *Geophysical Journal International* 227.3 (2021): 1938-1960.

Overall, this is a good work and promises to be one of high impact, especially if the two above points are addressed appropriately.

We thank the reviewer for recognizing the importance of this study, and we hope our revisions should address the issues raised by the reviewer.

Summary of the key results: see paragraph above.
Originality and significance: Unsure about originality, see paragraph above.
Data & methodology: Data of high quality, the approach is valid but some aspects are not considered, see paragraph above. The presentation is of high level.
Appropriate use of statistics and treatment of uncertainties: fully appropriate.
Conclusions: robust, valid, reliable. See paragraph above.
Suggested improvements: Experiments with more sophisticated GIA models if possible.
References: Appropriate credit is given to previous work.
Clarity and context: The abstract/summary is lucid and appropriate. I am not sure that the introduction is enlightening the novelty of this work.

Referees' comments:

Referee #1 (Remarks to the Author):

In my previous review, I raised three major concerns about this manuscript. The authors responded to my comments about using the method of citing references.

We thank the reviewer for helpful suggestions. Our response to each remark can be found below, highlighted in blue.

1.In my first concern, I mentioned that the sea level data between the geological-past and the instrumental post-1900 are not comparable in high resolution scale, because many sea level indices used in this work have very big uncertainties in indicating past sea level positions, with some up to 2 meters, but the recent instrumental sea level data is in millimeter-level resolution which are much more accurate. The authors emphasize that their method has been used to analyze instrumental together with geological data, and can transfer tidal gauge data from monthly/yearly to decadal resolution. While transferring data from monthly/yearly to decadal or centennial resolution is understandable, transfering the meter-scale resolution of the past sea level to millimeter-level resolution of the recent cannot be accurate. Since the core conclusion is that the GMSL rise rate since 1900 CE has exceeded any centuary over the past 4000 years, more comparable data from the geological-past will be needed.

We thank the reviewer for raising this point regarding the geological data resolution. We would like to clarify that our approach does not transfer geological data to millimeter-level resolution. Specifically, we employ three distinct data sources with different purposes:

(1) Modern tide gauge data: Transferred to decadal resolution with centimeter-to-decimeter uncertainty during the smoothing process.

(2) High-resolution geological data (last 3000 years): This is an updated database from Walker et al., 2022 (as stated in our Methods). Many with sub-decadal temporal and sub-decimeter vertical resolution, which are directly comparable to processed tide gauge data.

(3) Lower-resolution China geological data: With relatively low spatiotemporal resolution but covers sea-level change during the last 11,700 years.

We primarily use data source (3) to calibrate our GIA model's GMSL component, capturing millennial-scale evolution over the last 11,700 years. In contrast, sources (1) and (2), with comparable resolutions and uncertainties, are integrated to train our Gaussian Process model for capturing centennial-scale GMSL variability that the GIA model cannot resolve. Therefore, our major conclusion about rapid GMSL rise derives from the combination of all three data sources within a statistical framework that explicitly accounts for each dataset's uncertainty structure, ensuring appropriate data fusion without artificially enhancing resolution.

2.My second concern is that, many C14 ages used in this manuscript have too big uncertainties (some up to 200 years) and therefore it is hard to get a reliable centennial-resolution age profile of the last 4000 years. The authors admit that 48/88% of their data exhibit 1 sigma chronological uncertainty of <100/200 years. Although the authors emphasize their data quality is better than the published data by Hijma et al. (Nature, 2025, 652-657, Global sea-level rise in the early Holocene revealed from North Sea peats), but hijma et al’s

paper is about the early Holocene sea level, and that topic does not need a very high resolution of age profile. For comparing with recent instrumental records which have very accurate ages, the ages for the past should be more precise. I am glad to know that the authors are proposing a new project to carry on additional fieldwork to improved their data's resolution, and I believe which will be more convincing.

We thank the reviewer for this insight regarding chronological uncertainties. As stated in point 1, our China sea-level database serves a same purpose to Hijma et al. (2025) in constraining millennial-scale early and middle Holocene trends. Therefore, we suggest it is reasonable to compare our China sea-level database uncertainty distributions to those reported in Hijma et al., 2025.

For our centennial-scale GMSL analysis underlying the main conclusion, we employ a high-resolution global geological database (updated from Walker et al., 2022) with temporal and vertical uncertainties almost comparable to smoothed tide gauge data. This global dataset specifically targets the last 3000 years and provides the high-resolution chronological constraint needed for quantifying centennial-scale GMSL variability and enabling robust century-scale comparisons.

Therefore, our comparison of post-1900 rates to geological GMSL rate relies on comparable high-resolution data, while the lower-resolution China data serves its intended purpose of constraining long-term millennial-scale GMSL change.

3. My third suggestion is to remove the parts discussing about data earlier than 4000 years, since the main conclusion is mainly from the data of the last 4000 years. (Your reason?) If the authors insist to keep this part, I respect the authors' idea.

We thank the reviewer for this understanding. We suggest retaining the early Holocene content for several reasons: First, it demonstrates that modern GMSL-dominated sea-level changes differ from the last 4000 years of stable GMSL but resemble the early-middle Holocene when GMSL was the primary driver, showing readers that such GMSL-dominated budgets have occurred previously in the Holocene. Second, the longer record extracts geological controls on natural vertical land motion, establishing baseline conditions before anthropogenic impacts. Finally, this comprehensive Holocene framework provides essential context for understanding the magnitude of recent changes.

Walker, Jennifer S., et al. (2022) "Timing of emergence of modern rates of sea-level rise by 1863." Nature communications 13.1, 966.

Hijma, M.P., et al. (2025) “Global sea-level rise in the early Holocene revealed from North Sea peats”. Nature, 639(8055), pp.652-657.

Referee #3 (Remarks to the Author):

I have now reviewed your revised manuscript, as well as the letter addressing reviewer comments.

The revised manuscript has original sections 1 and 2.1 substantially edited (sea-level budget decomposition equations moved to Methods section) and this has improved the manuscript. I regard the attention given to (i) full-holocene sea-level budget, (ii) late holocene sea-level budget and geographic differences therein, (iii) modern time accelerated sea-level rise and subsidence now well balanced: clarity of results is much improved.

We thank the reviewer recognizing our efforts to improve the readability of this manuscript. Our response to each remark can be found below, highlighted in blue.

The quality of data (via amending the supplement) and the presentation of data and results (via the revision) is improved: the text communicates the findings well and addresses aspects in a logical order. I no longer uphold my previous concerns whether the modern day subsidence analysis was fitting to include: the balance found in the revised manuscript allows to keep it in, and I think that these are indeed meaningful statements to make for the chain of Chinese coastal (mega)cities, adding to paper impact.

We thank the reviewer for agreeing with our intention to include modern-day subsidence in the main text, as it represents one of our major conclusions.

My previous technical concerns regarding trade-offs between GMSL and GIA_GRD terms were well addressed in the letter, and through the textual revisions it triggered. The same goes for the remark on the decomposed subsidence signal in the big deltas, the land-ocean mask, and further minor comments. I am satisfied with the response. I have no remaining or new technical concerns.

We thank the reviewer for recognizing our efforts to address the technical concerns regarding GMSL and GIA_GRD trade-offs, subsidence signal decomposition in deltaic regions, and land-ocean mask considerations. We appreciate the reviewer's acknowledgment that our revisions and responses have satisfactorily resolved these complex methodological issues.

A minor remark concerns lines 538-541 and 547 and 554 (revised manuscript, Methods subsection 5.4) and the word 'overestimate' in there, implemented in response to my previous review. Because of the sign difference between uplift and subsidence, it is unclear what is meant exactly with 'overestimate'. Does one overestimate subsidence when one predicts uplift instead of subsidence?? I think you mean to say that using the modern land mask (main run) and using a paleo land mask (alternate run described in line 542-551) produce two bounding results for the GRD values, that sought for truth should be within. The 'GRD overestimate' that is discussed from line 554 onwards: it is not fully explicit, if that concerns the number produced in the main run (dubbed 'overestimate' as it is compared to a paleo-land-mask run) or the land movement according to the paleo-land mask run. Line 561 say it is an overestimation of the subsidence rates (not inland GRD uplift rate), which then adds to my confusion. The main text lines 157-161 and the response in the letter addressing comments is more clear than this part of the revised text. I think with some editing of section 5.4 this can be resolved/clarified. I am very happy that this caveat of doing Holocene sea-level budget analysis for large deltas, is explicit part of your treatment of the topic: I think it does matter for urban geology

usage of the findings of the paper, and further natural vs. human-induced subsidence attribution in mega deltas.

We thank the reviewer for highlighting the confusing use of overestimate in Methods section. We have clarified that our original GIA model with modern topography overestimates GRD signals compared to the paleo-topography scenario, consequently overestimating the compensatory linear subsidence term. We fully agree that large delta analysis is critical for Holocene sea-level budget studies and requires continued research for better urban geology applications.

Regarding the title "Profound modern sea-level evolution against geological backdrop in southeastern China": you might want to make it e.g. ".. modern sea-level _and_land_movement_ evolution against geological backdrop ..." - since you analyse the latter extensively.

We thank the reviewer for this helpful suggestion. However, Nature's 75-character title limit prevents including additional detailed information. We have therefore revised our title to "Sea level in southeastern China now rising faster than over past 4000 years" to better reflect our main finding while staying within the character constraint.

+++ ADDENDUM

The editor requested to append an advice regarding remaining points raised by reviewer1.
This considers fundamental differences in temporal and vertical resolution and accuracy between instrumental era and Holocene geological data. I agree with reviewer1 and authors that this requires specific handling (e.g. transforming instrumental data to decadal means) and that the benefits of combining these is emphasized in the paper. Unlike reviewer1, I think this is appropriately handled and addressed sufficiently explicit in the study. Some specific reflection:

- It is true that the geological input data is from various background (compiled from a few decades of dispersed regional studies) and has variable age-depth accuracies, but the statistical setup (multiple ‘kernels’ in the phrasing of the authors) addresses that there is ‘noise’/‘error’/’signal to be attributed to data collection nature’ (‘local factors’).

- I do not share the reviewer1 concerns regarding detection of the present GMSL acceleration (exceeding the Late Holocene background rate), as the acceleration does not occur at the break from geological to instrumental data, but gradually within the instrumental data covered period. The call of reviewer1 to have geological data for the pre-instrumental age as accurate as instrumental data, I reckon is impossible to meet. I don’t think it is needed to make the claims the paper does either. In the end, the paper provides a budget analysis for China’s _relative_ sea-level record, synthesizing Holocene geological data (the word ‘backdrop’ in the paper title), so that analysis of instrumental data -- at their original high temporal and vertical resolution -- is supplied by meaningful, reproducible natural rates. This thus lifts up geological data and overcomes resolution caveats, for engineering and other societal use. The scientific goal is not to achieve the same resolution in the past as we have in the present.

- I found the treatment of variation in sea-level in the last 4000 years quite developed. These trends (statistically unlocked by kernels of centennial time length and few 100 km spatial range) were statistically detected, despite the lower age-depth accuracy.

- I agree that with sea-level data and its statistical processing alone (geologically observation in isolation), there are different trade offs imaginable: with the current numbers of individual observations, there is still risk of misreading sampling bias as geological signals. But the statements on the last 4000 year were not solely the geological sea-level that defines the state of research: for the last 4000 years, the authors cross-validate their inference referencing extensive hydro-climatological literature and providing mechanistic calculus providing causal supportive explanation. There is similar trade-off care in the treatment of natural subsidence and the validation of that against instrumental era data (which in critical areas records human accelerated subsidence).

We thank the reviewer for this thoughtful assessment and for recognizing that our statistical framework appropriately addresses the differences between geological and instrumental data resolutions.

Referee #3 (Remarks on code availability):

no comments

Referee #5 (Remarks to the Author):

Dear Editor
I am satisfied with the authors' response.
Best
G

We thank the reviewer recognizing our efforts to improve this manuscript.
